# Subtyping based on immune cell fractions reveal heterogeneity of cardiac fibrosis in end-stage heart failure

**DOI:** 10.3389/fimmu.2023.1053793

**Published:** 2023-02-15

**Authors:** Shangjie Zou, Bee Luan Khoo

**Affiliations:** ^1^ Department of Biomedical Engineering, City University of Hong Kong, Hong Kong, Hong Kong SAR, China; ^2^ Hong Kong Center for Cerebro-Cardiovascular Health Engineering (COCHE), Hong Kong, Hong Kong SAR, China; ^3^ Department of Precision Diagnostic and Therapeutic Technology, City University of Hong Kong-Shenzhen Futian Research Institute, Shenzhen, China

**Keywords:** heart failure, cardiac fibrosis, immune cell fractions, subtyping, functional gene sets

## Abstract

**Background:**

A central issue hindering the development of effective anti-fibrosis drugs for heart failure is the unclear interrelationship between fibrosis and the immune cells. This study aims at providing precise subtyping of heart failure based on immune cell fractions, elaborating their differences in fibrotic mechanisms, and proposing a biomarker panel for evaluating intrinsic features of patients’ physiological statuses through subtype classification, thereby promoting the precision medicine for cardiac fibrosis.

**Methods:**

We inferred immune cell type abundance of the ventricular samples by a computational method (CIBERSORTx) based on ventricular tissue samples from 103 patients with heart failure, and applied K-means clustering to divide patients into two subtypes based on their immune cell type abundance. We also designed a novel analytic strategy: Large-Scale Functional Score and Association Analysis (LAFSAA), to study fibrotic mechanisms in the two subtypes.

**Results:**

Two subtypes of immune cell fractions: pro-inflammatory and pro-remodeling subtypes, were identified. LAFSAA identified 11 subtype-specific pro-fibrotic functional gene sets as the basis for personalised targeted treatments. Based on feature selection, a 30-gene biomarker panel (ImmunCard30) established for diagnosing patient subtypes achieved high classification performance, with the area under the receiver operator characteristic curve corresponding to 0.954 and 0.803 for the discovery and validation sets, respectively.

**Conclusion:**

Patients with the two subtypes of cardiac immune cell fractions were likely having different fibrotic mechanisms. Patients’ subtypes can be predicted based on the ImmunCard30 biomarker panel. We envision that our unique stratification strategy revealed in this study will unravel advance diagnostic techniques for personalised anti-fibrotic therapy.

## Introduction

Heart failure results from the progression of cardiovascular diseases, such as coronary artery diseases and cardiomyopathy (1). There are two types of heart failure: ischemic heart failure and non-ischemic heart failure. Ischemic heart failure is typically caused by blood vessel diseases that result in myocardial infarction and cardiomyocyte death (1), whereas non-ischemic heart failure is caused by genetic mutations, infections, or exposure to stimuli that result in myocardium dysfunction (2). Early-stage heart failure is typically accompanied by loss of functional cardiomyocytes, hypertension, and increased ventricular wall stress, all of which contribute to inflammation and pro-fibrotic signaling (3). As a result, fibrosis is a common pathological process in the progression of heart failure caused by both etiologies (2, 3), and it is commonly regarded as a biomarker for poor prognosis (4–6) as well as the hallmark of end-stage heart failure (7).

Although fibrosis was initiated as a means of healing, it would eventually lead to decreased ventricular compliance, abnormal cardiac rhythm, and cardiomyocyte death, worsening the symptoms of heart failure (8). Conventional heart failure therapies, which focuses on symptom relief rather than maladaptive remodeling processes such as cardiac fibrosis (9, 10), would lead to varied outcomes among patients (11). Current clinical research on anti-fibrosis treatments has focused on directly reversing the activated cardiac fibroblast phenotype (11, 12). However, most of these treatments remain unsatisfactory.

Previous research has shown that, while activation of immune responses and immune cells is required for cardiac repair, excessive activation is a major cause of fibrosis (3, 13). Nonetheless, the lack of understanding of the interplay between cardiac fibroblasts and the immune cell fractions prevents therapeutic agents from targeting the pro-fibrotic regulatory networks underlying fibroblast activation (12, 14–18). For example, endothelin inhibitors, anti-TGFβ, and anti-inflammatory drugs target fibrotic pathways but had a high degree of inconsistency in treatment efficacy within patients (19, 20), and were even reported to increase the risk of adverse immune responses (21). These unsatisfactory results can partially due to our limited understanding of the complex relationships between cardiac fibrosis and immune cells. For example, the overlapping impact of M1 and M2 macrophages on promoting fibrosis through different approaches is attributed to the dual-pronged roles of immune activation, despite having opposite regulatory functions on inflammation (13). Neutrophils, monocytes, T cells, and B cells were thought to regulate fibrosis, but crosstalk and fibrosis regulation (promoting or suppressing) are related to pathological statuses and immune cell populations (13, 22). CD4^+^ T cells mainly promote fibrosis through secreting cytokines like IFN-γ, IL-4, IL-13, and IL-17 (23). B cells can secret immunoglobulins to directly induce fibrosis (24). Monocyte promotes inflammation and immune cell infiltration (13). M2 Macrophage reduce inflammation, but promotes fibrosis through the process of tissue healing and cardiac remodeling (13). These evidences in former reports indicates that immune cell fraction is an important causal factor for fibrotic mechanisms in heart failure.

As a result, finding a therapy targeting immune response that can achieve consistent results in all patients would be difficult without considering their local conditions of immunity on a more systematic scale.

Understanding the interrelationship between cardiac fibroblasts and their immune cell fractions holds promise for breakthroughs in effective anti-fibrosis therapy (11). Furthermore, heart failure and cardiac fibrosis are also present with complex symptoms and multiple etiologies, which brings a tremendous amount of challenge to achieving individualised treatment strategies (25, 26). There is an urgent need for a better understanding of the heterogeneity in immune cell fractions and its relationship to fibrosis as an important prerequisite for effective anti-fibrosis strategies.

Here, we report for the first time the establishment of a matrix of cardiac immune cell fractions for heart failure patients and validated the subtyping analysis based on that. Based on cardiac immune cell fractions, two distinct patient subtypes were identified: [1] pro-inflammatory (with elevated levels of biomarkers related to adaptive immune responses) and [2] pro-remodeling (with high M2 macrophage). We demonstrated that epithelial-mesenchymal transition (EMT) was highly correlated with fibrosis in both subtypes. A novel analytical method based on Large-Scale Functional Score and Association Analysis (LAFSAA) identified functional gene sets related to subtype-specific mechanisms of EMT and fibrosis. Fibrosis of the pro-inflammatory subtype was specifically correlated with chronic inflammatory responses, JAK activities, and hemidesmosome. In contrast, fibrosis of the pro-remodeling subtype was specifically correlated with IL-6 receptor binding, glycoside metabolic process, leukocyte aggregation, and blood vessel development. We then present a unique biomarker panel, termed ImmunCard30, based on 30 genes encoding secretory proteins for patient stratification. The signature score of ImmunCard30 achieved high subtype classification efficiency, with the area under the receiver operator characteristic curve (AUC) equal to 0.958 in the discovery set (n = 103) and 0.79 in the validating set (n = 177). ImmunCard30 could potentially serve as the biomarker panel for diagnosing patients’ subtypes and guide therapeutic treatments targeting the subtype-specific pro-fibrotic mechanisms. Overall, this study provides new insights into the heterogeneous cardiac immune cell fractions and its interactions with fibrosis, thereby providing an important basis for anti-fibrotic drug screening and disease management strategies. The novel patient stratifying strategy and the ImmunCard30 biomarker panel provide the basis for an important step towards precise anti-fibrotic therapies that surpass the current treatments.

## Materials and methods

### Data installation and preparation

The transcriptomic datasets were obtained from the Gene Expression Omnibus (GEO) database. Five datasets of ventricular tissue samples were involved, among which three of them were used to form the discovery set: GSE116250 (27), GSE135055 (28), and GSE46224 (29). Independent dataset GSE57338 (30) was used as the validating set. In total, 103 samples were included in the discovery set, and 177 samples were included in the validating sets. Two datasets from lung tissues were involved in validating signature scores of fibrosis: GSE124685 (31) and GSE184316. Raw data of RNA-sequencing was transformed into reading counts using Genome Reference Consortium Human Build 38 as the reference genome. The read counts of RNA-sequencing were further normalised to Reads Per Kilobase of transcript per Million reads mapped (RPKM) by R package *edgeR* (R 4.1.0) (32).

Apart from the transcriptomic datasets, this study also involved information from public biomedical databases. Annotated gene sets of EMT and angiogenesis were retrieved from hallmark gene sets of MSigDB collections (33, 34). The list of secretory proteins was obtained from The Human Protein Atlas (https://www.proteinatlas.org/) (35).

### Quantification and clustering of the immune cell fractions

CIBERSORTx calculated the matrix of samples’ immune cell fractions based on the 22-leukocyte signature matrix (LM22) (36, 37). The calculated matrix of immune cell fractions serves as the input of clustering to identify subtypes of the immune cell fractions. The average silhouette width was applied to evaluate optimal numbers of clusters (38), while k-means clustering was conducted based on R package *factoextra* (R 4.1.0) (39). Visualisation of k-means clustering was achieved by dimensional reduction based on t-distributed stochastic neighbor embedding (tSNE) implemented by R package *RtSNE* (R 4.1.0) (40). Recursive feature elimination for ranking immune cell types on their importance in determining subtype affiliations was achieved by R package *caret* (41).

### Transcriptomic analysis and statistical analysis

Differential gene expression analysis was conducted based on R package *DESeq2* (R 4.1.0) based on read counts of RNA-sequencing normalised by datasets (to remove potential batch effects) (42). Functional enrichment analysis was conducted by the online tool *Metascape* (https://metascape.org/) to study enriched gene sets in Gene Ontology Biological Process, Kyoto Encyclopedia of Genes and Genomes, and Hallmark gene sets (43). Correlations between variables were evaluated by Pearson correlation with R scripts (4.1.0). Signature scores were calculated based on R package *singscore* (R 4.1.0) (44). Scores of fibrosis, scores of EMT, and scores of functional gene sets were calculated by *singscore* using a uni-directional mode.

In contrast, the score of the predictive gene panel for subtype classification was calculated based on the bi-directional *singscore* method (“bi-directional” means considering both positively- and negatively-correlated genes in the scoring). Feature extraction based on least absolute shrinkage and selection operator (LASSO) regression was performed by R package *glmnet* (R 4.1.0), using the variables corresponding to the highest ln (λ) value that resulted in a misclassification error within 1 standard error of the minimum cross-validated error as the optimal variable combination (45). ROC curves and AUC were plotted and calculated by R package *pROC* (R 4.1.0) (46).

### Implementation of LAFSAA

By applying *singscore (* 44) method, we calculated signature scores of the 10402 ontology gene sets and 50 HALLMARK gene sets retrieved from MSigDB (33) based on the expression matrix of the 103 samples from the discovery set. The matrix containing samples on the rows, functional gene sets on the columns, and signature scores as values was named the quantitative functional matrix. Spearman correlation analysis was applied to screen the gene sets in the quantitative functional matrix. Those gene sets that were significantly and specifically correlated with the target gene set (TGS, which was the HALLMARK gene set of EMT in this study) in samples from one subtype would be identified as pro-inflammatory or pro-remodeling subtype-specific gene sets. The identified gene sets would be further screened based on their correlations with EMT in their corresponding subtypes in the validating set GSE57338.

In the meantime, to make the screening more stringent, we applied concept signature enrichment analysis (CSEA) (47) based on the genes that were differentially expressed between the high and low fibrosis groups in each subtype. Only gene sets with *p*-value< 0.05 in CSEA analysis of their corresponding subtypes were regarded as subtype-specific EMT-related gene sets.

### Calculation of intra-cluster correlation scores

Intra-cluster correlation scores were used to select representative gene sets for clusters identified based on the correlation matrix. It denotes the average coefficients of a gene set’s correlations with other gene sets in its corresponding cluster. This score was calculated by the equation below:


(1) $$ICS_{\left(GS_{i}\right)}=\frac{\sum_{j=1}^{n}SpearmanCor(GS_{i},GS_{j})}{n}$$

Where *ICS_(GSi)_* is the intra-cluster correlation score of the *i^th^* gene set. The *n* denotes the number of gene sets in a cluster. *SpearmanCor(GS_i_, GS_j_)* denotes the spearman correlation of the *i^th^* and the *j^th^* gene set.

## Results

### Clustering of immune cell fractions for subtyping

Samples from patients with end-stage heart failure (n = 103) and healthy samples (n = 31) from three datasets were retrieved (**Table 1**). Since infiltrated immune cells are the major components and central regulators of cardiac immune responses, we applied CIBERSORTx to calculate immune cell fractions for all the involved patient samples (36) (**Supplementary Table 1**), and conducted subtyping based on the matrix of immune cell fractions.

**Table 1:** Clinical characteristics of samples from discovery set.

Characteristics	NICM (n = 74)	ICM (n = 29)
Age (years)	46.26 ± 13.29	59.6 ± 4.55
Gender	53 males; 21 females	26 males; 3 females
Race		
White/Caucasian, n (%)	43 (58.11)	25 (86.21)
Black/African, n (%)	7 (9.46)	4 (13.79)
Yellow/Asian, n (%)	21 (28.38)	–
Unknown, n (%)	3 (4.05)	–
NYHA functional score or class		
GSE116250	3.3 ± 0.6 (n = 37)	3.3 ± 1 (n = 13)
GSE46224	stage III or above (n = 16)	stage III or above (n = 16)
GSE135055	stage II (n = 2); stage III (n = 7); stage IV (n = 12)	–
LVEF (%)*	20.62 ± 8.36	16.16 ± 6.05
Comorbidities		
Coronary artery disease, n (%)	4 (5.41)	29 (100)
Smoking history, n (%)	30 (40.54)	20 (68.97)
Diabetes mellitus, n (%)	14 (18.92)	16 (55.17)
Medications and treatments		
Beta Blockers, n (%)	47 (63.51)	20 (68.97)
Inotropes, n (%)	17 (22.97)	7 (24.14)
Aspirin, n (%)	31 (41.89)	24 (82.76)
Amiodarone, n (%)	27 (36.49)	13 (44.83)
ACE inhibitor/Angiotensin receptor blocker, n (%)	34 (45.95)	14 (48.28)
Statins, n (%)	18 (24.32)	28 (96.55)
LVAD/BiVAD, n (%)	24 (32.43)	12 (41.38)

*LVEF data from GSE46224 was not precise. Therefore, the LVEF data shown in this table only serves as a reference. The samples were retrieved from three datasets on the Gene Expression Omnibus database: GSE116250, GSE135055, and GSE46224. Plus-minus values are means ± one standard deviation. *NYHA* New York Heart Association. *GSE* Gene Expression Omnibus series. *LVEF* left ventricular ejection fraction. *ACE* Angiotensin-Converting Enzyme. *LVAD/BiVAD* left/biventricular assist device.

**Figure 1:**
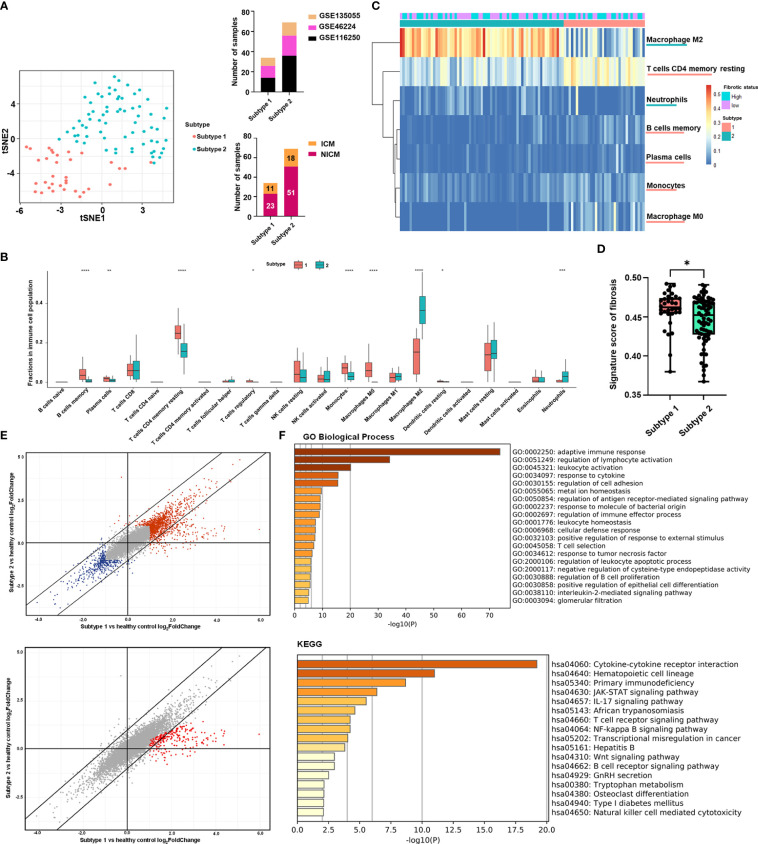
Clustering of heart failure samples for subtyping stratification. **(A)** Left panel: clustering of the two subtypes was visualized by t-distributed stochastic neighbor embedding (t-SNE). Red dots represented subtype 1 samples, while turquoise dots represented subtype 2 samples. Right panel: distribution of heart failure samples from different datasets (upper panel) and with different diagnoses (lower panel) in the two subtypes. Datasets were displayed with their respective Gene Expression Omnibus Series (GSE) identities. The samples’ diagnosis was categorized into ischemic cardiomyopathy (ICM) and non-ischemic cardiomyopathy (NICM). **(B)** Boxplot showing the immune cell fractions of the 22 cell types in the two subtypes. Red and cyan boxes, respectively, represented subtypes 1 and 2. The significance of the difference in immune cell fractions between the two subtypes calculated by the two-tailed Student’s t-test was shown on the top of each cell type’s column. **(C)** Heatmap showing the matrix of significantly different immune cell fractions between the two subtypes. Rows represented the immune cell types annotated on the right side of the heatmap. Each column represented one sample, while column annotations on the top of the heatmap described the corresponding samples’ subtype belongings and fibrotic status. **(D)** Boxplot showing the signature score of fibrosis in the two subtypes. Red and cyan boxes, respectively, represented subtypes 1 and 2. Each black point represented one sample in its corresponding subtype. The significance of the difference in score between the subtype 1 (n = 34) and the subtype 2 (n = 69) calculated by the two-tailed Student’s t-test (t = 2.187, df = 101) was shown at the top of the plot. For each boxplot: the center line represents the median, the box represents the interquartile range, and the whisker displays minimum to maximum. (P-value< 0.05: *< 0.01: **< 0.001: ***< 0.0001: ****). **(E)** Scatter plots showing log2(fold-change) of genes in the two subtypes compared with healthy controls. Each dot represented one gene. In the upper panel, genes with log2(fold-change) higher than 1 or lower than -1 in one of the subtypes were highlighted in red or blue, respectively. In the lower panel, genes with log2(fold-change) in subtype 1 that were two times higher than in subtype 2 were highlighted in red. **(F)** Functional enrichment analysis of the genes highlighted in red in **(E)** lower panel. The bar plots were -log10 (P-value) of enrichments of genes in signatures of Gene Ontology (GO) Biological Process and Kyoto Encyclopedia of Genes and Genomes (KEGG) of the Molecular Signatures Database.

We identified two potential clusters based on the matrix of immune cell fractions, which was the average silhouette width method (**Supplementary Figure 1**). We demonstrated that k-means clustering classified the tissue samples (n = 103) into two subtypes, termed Subtype 1 and 2 (**Figure 1A**). Samples were assigned to Subtype 1 (n = 34) and Subtype 2 (n = 69), respectively (**Figure 1A**). Checking on samples’ etiologies and source datasets indicated that clustering results were not influenced by dataset attribution or underlying diagnosis (**Figure 1A**; **Supplementary Table 2**).

Based on boxplots showing comparisons of immune cell proportions, type 2 subtypes were higher in neutrophils and M2 macrophages, whereas type 1 was higher in B cells, T cells, and monocytes (**Figure 1B**). **Figure 1C** shows a heat map showing significantly different immune cell levels between the two subtypes.

To quantify the fibrotic status of the samples, we need to select marker genes that can represent fibrosis. *POSTN*, *COL1A1*, *TIMP1*, *MMP2*, and *ACTA2*. *POSTN* and *COL1A1* are genes that express the extracellular matrix proteins periostin and type 1 collagen, both of which are significantly up-regulated in heart failure with cardiac fibrosis (48). *TIMP1* and *MMP2* are enzymes that aid in the progression of cardiac fibrosis (49). *ACTA2* is a fibroblast activation biomarker (50). In previous studies, these direct fibrosis marker genes were used to assess pro-fibrotic activity (51). The same five-marker combination has been used to indicate fibrotic levels in patients (50). Fibrosis signature scores based on these five biomarkers were validated on multiple organ fibrosis datasets corresponding to heart and lung tissue, showing significant differences between normal and fibrotic samples (**Supplementary Figure 2**). We applied signature scoring to the 103 tissue samples and divided the samples into high and low fibrosis groups based on the median of fibrotic signature scores. Overall, Subtype 1 (n = 34) had a slightly higher fibrosis score than Subtype 2 (n = 69) (**Figure 1D**), but both high and low fibrosis samples were seen in both subtypes (**Figure 1B**).

To further evaluate the differences in fibrosis-related factors between the two subtypes at the molecular level, we explored the differentially expressed genes (DEG) separately in the two subtypes relative to healthy controls. DEGs in both subtypes had similar fold changes to healthy controls (**Figure 1E**). However, 286 genes showed enhanced heart failure-related up-regulation in Subtype 1 (2 times higher in log2 fold change) than Subtype 2 (**Supplementary Table 3**; **Figure 1E**).

Among the genes with the highest levels of upregulation in subtype 1: The functional enrichment analysis ranked the Gene Ontology gene set “adaptive immune response” first, with multiple other gene sets related to adaptive immune responses (for example, regulation of lymphocyte activation, T cell selection, T cell receptor signaling, and regulation of B cell proliferation) identified as significantly enriched as well (**Figure 1F**). Other inflammatory signaling pathways involved in adaptive immune responses, including those related to IL-2, JAK-STAT signaling, and IL-17 signaling, were also more activated in the subtype 1 (**Figure 1F**).

As a further demonstration, we selected a list of genes as typical biomarkers of adaptive immune response, which included multiple surface antigens of T cells (such as CD8A, CD8B, CD3G), NK cells (such as CXCR3, CD7), or B cells (such as CXCR5, CD79A), and genes involved in signaling of chemokines and cytokines that regulates adaptive immune responses. According to the differential gene expression analysis, these genes showed higher up-regulation compared to healthy controls in the subtype 1 than in the subtype 2 (**Supplementary Figure 3A**).

In the meantime, the subtype 1 was higher in proportion of CD4^+^ T cells, regulatory T cells, memory B cells, and plasma cells (**Figure 1B**). The high percentage of T and B cells indicated an active adaptive immune response. Furthermore, to directly compare the differences in activation of T cells and B cells between the two subtypes, we calculated signature scores of multiple functional gene sets related to B cell activation, T cell proliferation, and T cell extravasation (all of which are crucial processes in adaptive immune responses), and demonstrated that subtype 1 was higher in all of these gene sets (**Supplementary Figure 3B**). Meanwhile, so as to evaluate the activity of B cells, we have also calculated the expressional score of immunoglobulins based on expression level of 188 genes that express immunoglobulins (calculated by “*singscore*” method). The obtained expressional score of immunoglobulins was significantly higher in the subtype 1, indicating a higher activity of antibodies in the subtype 1 (**Supplementary Figure 3B**).

In conclusion, biomarker and functional gene set analyses revealed that the two immune cell subtypes were associated with different levels of biomarkers related to adaptive immune responses. Further research on patient samples using comprehensive clinical recordings or rationally designed disease models is expected to provide more evidence to validate this correlation in the future.

### Distinct immune cell types related to subtype stratification and fibrosis

**Figure 2:**
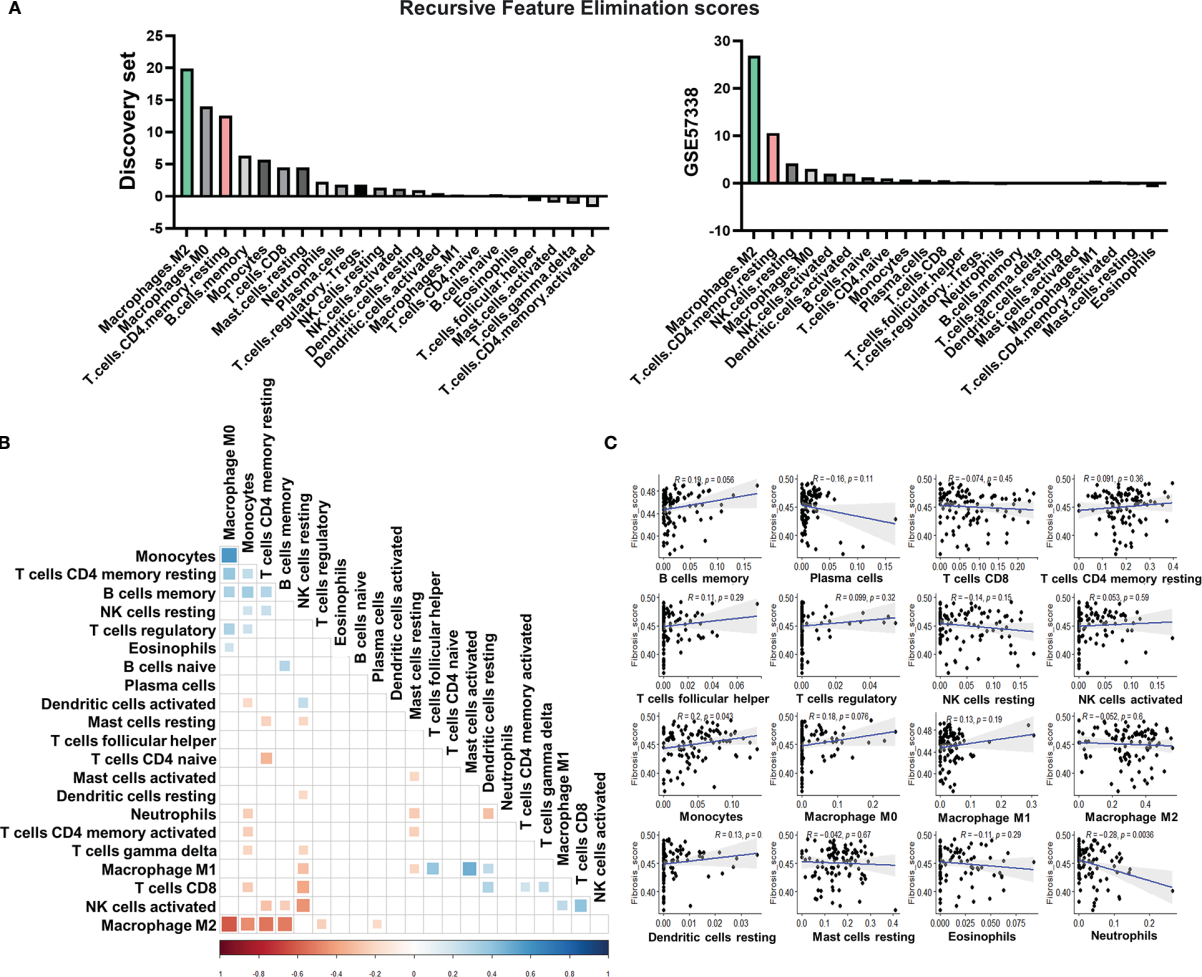
Immune cell types related to subtype stratification and fibrosis. **(A)** Rank of immune cell types based on their scores calculated by recursive feature elimination. **(B)** Pearson correlations between immune cell types. The colored square in each rectangular cell displayed the correlation coefficient between immune cell types on its corresponding row and column. Red squares indicated negative coefficients, while blue squares indicated positive coefficients. Darker shades and larger square sizes indicated a higher absolute value of coefficients. Only correlations with P-value less than 0.05 were displayed with correlation coefficients. **(C)** Scatter plots with Pearson correlation coefficients between fractions of each immune cell type and the signature score of fibrosis. Blue light in each subgraph represented the linear regression line. Each black dot represented a heart failure sample. The light gray area surrounding each blue regression line represented a level 0.95 confidence interval.

We scored and ranked 22 leukocyte cell types based on recursive feature elimination according to their importance in determining subtype affiliations (**Figure 2A**). To validate the ranking, we applied CIBERSORTx to an independent dataset with a large sample size (n = 177): GSE57338 (30) and assigned samples to two subtypes by calculating their distance from the centroids of the two subtypes in the discovery set (**Supplementary Figure 4**).

According to the result of recursive feature elimination, the proportion of M2 macrophages, which was significantly higher in the subtype 2, was robustly the most important feature that discriminated the two subtypes (**Figure 2A**). The proportion of resting CD4^+^ T cells was identified as the most important feature for the subtype 1 (**Figure 2A**). With the distinct phenotype of elevation in resting CD4^+^ T cell proportions, leading to high immune response activation, Subtype 1 was subsequently referred to as the pro-inflammatory subtype. The high level of M2 macrophages, which plays a pivotal role in anti-inflammation and pro-remodeling in fibrotic tissues (52), is the distinct phenotype of Subtype 2, termed the pro-remodeling subtype.

**Figure 2B** depicts a broad overview of immune cell type correlations. CD4+ T cell levels were found to be positively correlated with monocytes, M0 macrophages, and memory B cells, indicating that these cell types have close regulatory relationships in the pro-inflammatory subtype. M2 macrophage levels, on the other hand, were significantly inversely correlated with the proportions of monocytes, T cells, and B cells (**Figure 2B**), confirming the role of macrophage M2 in inflammation suppression (53) and its potentially key role in determining the immunity of the pro-remodeling subtype, which was distinct from the pro-inflammatory subtype.

We calculated the Pearson correlation coefficient between the fractions of various immune cells and fibrosis to assess the potential contribution of immune cells to fibrosis (**Figure 2C**). The immune cells chosen were either important for classifying the two subtypes (for example, M2 macrophages, CD4+ resting T cells, memory B cells, and M0 macrophages) or had high proportions in the immune cell population (such as NK cells, mast cells). The proportion of monocytes was found to be positively correlated with fibrosis. The proportion of neutrophils, on the other hand, was negatively correlated. Despite being the most distinguishing features of the two subtypes, the proportions of M2 macrophages and CD4+ T cells were not significantly associated with fibrosis in our discovery set (**Figure 2C**).

### EMT was potentially a major contributor to fibrosis in both subtypes

In the previous analyses, we demonstrated that the major features of the two subtypes: the proportion of M2 macrophages and CD4+ T cells, were not significantly related to fibrosis (**Figure 2C**). As a result, subtype affiliation may not be a direct predictor of fibrotic levels. To better understand the fibrotic mechanisms in these two subtypes, we evaluated if there was a universal hub pro-fibrotic process induced in both subtypes.

**Figure 3:**
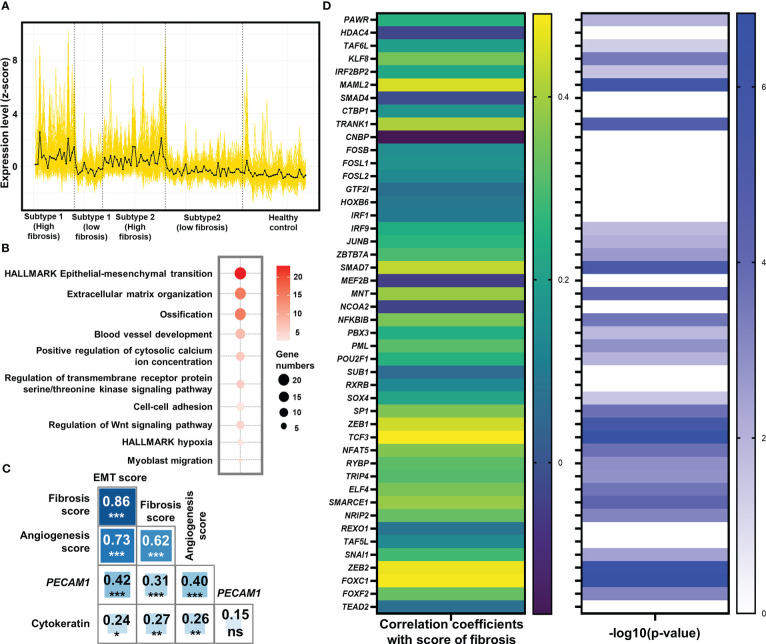
Identification of epithelial-mesenchymal transition (EMT) as the potentially pivotal pro-fibrotic process. **(A)** Expression of 166 common genes that were up-regulated in high fibrosis samples in both subtypes. The expression level was displayed by z-score. Each yellow line represented the line plot of a gene’s expression level across the 103 heart failure samples and 31 healthy samples. The black line represented the average of z-scores of the 166 genes among the samples. Vertical dash lines separated the samples from different groups, with their belongings shown below x-axis. **(B)** Bubble plot for functional analysis of the 166 up-regulated genes in high fibrosis samples. The deeper red color of the bubbles represented a higher -log10(P-value), while the larger circles indicated the higher number of genes identified in the corresponding gene set. **(C)** Pearson correlation coefficients between signature scores of fibrosis, EMT, and angiogenesis. Numbers and asterisks in rectangular cells represented correlation coefficients and P-values (ns, nonsignificance; Pvalue >0.05: *> 0.01: **> 0.001: ***> 0.0001: ****). **(D)** Pearson correlation coefficients (left panel) and -log10(P-value) of the correlations (right panel) between expression levels of the 46 key EMT-related transcription factors and score of fibrosis. Levels of correlation coefficients and -log10(P-value) were displayed with colors according to the legends on the right side of their respective panel. Only those genes with -log10 (P-value) > 1.30103 (P-value< 0.05) were displayed with color in the right panel.

By dividing the tissue samples (n = 103) using the median fibrosis score as a cutoff, we obtained high and low fibrosis groups in both the pro-inflammatory subtype (High: n = 20, 19.42%; Low: n = 14, 13.59%) and the pro-remodeling subtype (High: n = 31, 30.10%; Low: n = 38; 36.89%). The distribution of high and low fibrosis samples in the two subtypes can be seen in the column annotation of the heatmap **Figure 1C**. Differential gene expression analysis between the high and low fibrosis groups identified 166 commonly up-regulated genes in the high-fibrosis group of both subtypes (**Supplementary Table 4**; **Figure 3A**).

Functional enrichment analysis identified that a large number of commonly up-regulated genes in the high fibrosis group were associated with EMT (**Figure 3B**). Signature scores calculated based on the HALLMARK gene set of EMT demonstrated that EMT was highly up-regulated in heart failure patients, and the high fibrosis groups had significantly higher scores than low fibrosis groups (**Supplementary Figure 5**). There are normally two types of EMT in adult ventricular tissues: epicardial EMT and endothelial-mesenchymal transition (EndMT) (54). EndMT is commonly regarded as a subset of EMT due to shared pathways and transcriptional factors (55). The signature scores of EMT and angiogenic hallmark signatures were highly correlated with each other (coefficient = 0.73), as was the signature score of fibrosis (coefficient equals 0.86 for correlation between EMT and fibrosis, and equals 0.62 for correlation between angiogenesis and fibrosis) (**Figure 3C**), indicating potential relationships between vascular endothelial cell activities and EMT in the discovery set.

We studied the correlations of cytokeratin (expressional score calculated by “singscore” method based on 55 genes expressing cytokeratin) and *PECAM1* (CD31) with EMT and fibrosis to determine which type of EMT was the major contributor to fibrosis. Previously, cytokeratin was thought to be an epithelial cell feature, while *PECAM1* was thought to be an endothelial cell feature (56, 57). The correlation coefficient between *PECAM1* and EMT was 0.42, with a high significance (P 0.001), while the correlation between cytokeratin and EMT was much lower (coefficient = 0.24, P 0.05) (**Figure 3C**). These results showed that EMT in the discovery set was closely related to endothelial cells.

We discovered that endothelial biomarkers were highly correlated with EMT and fibrosis in patients with end-stage heart failure. Previous research has suggested that EndMT may contribute to cardiac fibrosis (58). As a result, more clinical cohort studies may be needed to confirm whether the identified EMT in end-stage heart failure patients, which was highly correlated with fibrosis, was related to EndMT.

At the level of transcriptional regulation, we screened 46 essential transcriptional factors associated with EMT based on previously reported regulatory landscapes (**Supplementary Table 5**) (59), of which 29 were significantly associated with fibrosis scores in the discovery set (all significant correlation coefficients were positive) (**Figure 3D**). These significant correlations served as strong evidences to prove the associations between EMT-related transcription factors and fibrosis, validating the pivotal role of EMT as a central process in fibrosis.

### Mechanistic study of EMT in the two subtypes based on LAFSAA

The stratification of heart failure patients into pro-inflammatory and pro-remodeling subtypes would unravel unique biomarkers related to their fibrotic mechanisms. Here we designed a novel approach based on LAFSAA to identify the functional gene sets that may be associated with distinct EMT mechanisms in the two subtypes (**Supplementary Figure 6**). We identified 17 gene sets specifically associated with EMT in the pro-inflammatory subtype and 87 gene sets specifically associated with EMT in the pro-remodeling subtype.

**Figure 4:**
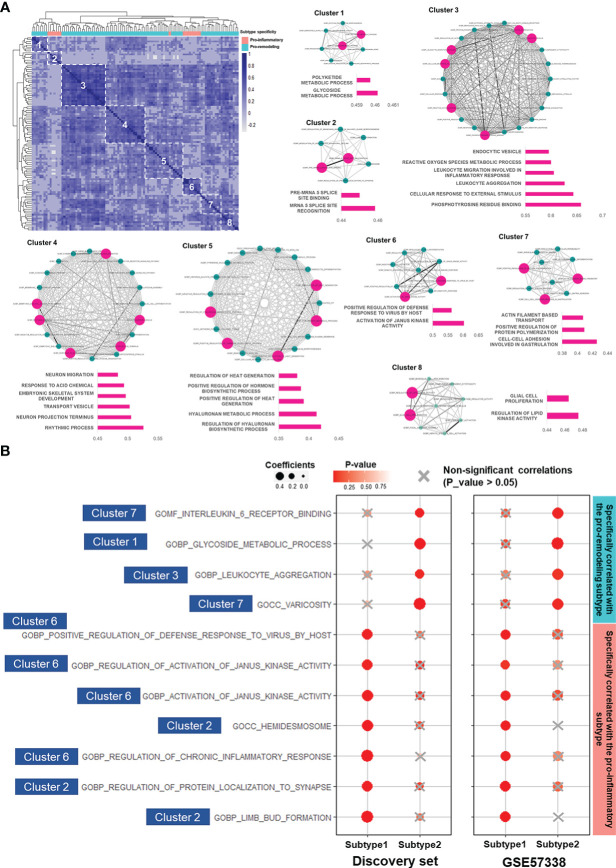
LAFSAA-identified subtype-specific pro-EMT and pro-fibrotic gene sets. **(A)** Heatmap on the top left shows the correlation matrix of LAFSAA-identified subtype-specific pro-EMT gene sets. Column annotation of the heatmap indicates the subtype-specificity of these pro-EMT gene sets. The gene sets were grouped into 8 clusters based on hierarchical clustering. Intra-cluster correlations were displayed in the form of a correlation network, with edges denoting the correlation coefficients between each pair of functional gene sets. Gene sets that were represent the functions of their corresponding clusters were marked in magenta, with their intra-cluster correlation scores displayed in their corresponding bar charts. **(B)** Subtype-specific pro-fibrotic gene sets and their Spearman correlation coefficients with fibrosis in the two subtypes in the discovery set and the validating set GSE57338. The gene sets were marked with their belonged LAFSAA-identified clusters.

To study the potential functional relationships between these 104 gene sets, we calculated their correlation matrix based on Spearman correlation coefficients in the discovery set. According to the correlation matrix, hierarchical clustering divided the 104 gene sets into 8 clusters with high intra-cluster correlations (**Figure 4A**). Gene sets assigned to the same cluster were expected to be functionally relevant. Gene sets with intra-cluster correlation scores (Calculated by Eq. 1) ranked in the top 30% of their corresponding clusters were especially noted to interpret functions of their corresponding clusters (**Figure 4A**).

Among the LAFSAA-identified functional clusters, clusters 1 (related to glycoside metabolic process), 3 (related to the immune response to environmental stresses), 4 (related to rhythmic process and neural development), 5 (hyaluronan biosynthesis and metabolism), and 7 (related to cell adhesion, cytoskeletal organisation, and vasculature development) were specifically correlated with EMT in the pro-remodeling subtype. In contrast, cluster 2 (related to translational regulation, neural system development, and cell adhesion) and 6 (related to JAK activities, inflammation and adaptive immune response) were specifically correlated with EMT in the pro-inflammatory subtype (**Figure 4A**).

Among the subtype-specific EMT-correlated gene sets from these 8 clusters, 11 of them (from clusters 1, 2, 3, 6, and 7 of LAFSAA-identified functional clusters) were also subtype-specific fibrosis-correlated gene sets, as shown in **Figure 4B**. Meanwhile, these 11 functional gene sets were mainly having similar signature scores between the two subtypes (**Supplementary Figure 7**). These results indicated that the 11 functional gene sets were likely represented the heterogeneous fibrotic mechanisms in different subtypes of immune cell fractions.

Among these 11 gene sets, 7 were specifically correlated with fibrosis in the pro-inflammatory subtype (**Figure 4B**). These 7 gene sets were mainly related to immune responses, including “GOBP: regulation of chronic inflammatory response” and “GOBP: activation of Janus kinase activity”. This result demonstrated that in the pro-inflammatory subtype, fibrosis was potentially promoted by EMT induced by inflammatory responses. Meanwhile, hemidesmosome, regulation of protein localization to synapse, and limb bud formation (related to cell morphogenesis) were also specifically correlated with EMT and fibrosis in the pro-inflammatory subtype, which might reflect the reorganization of extracellular matrix and morphological changes of cardiac cells.

In contrast, gene sets specifically correlated with fibrosis and EMT in the pro-remodeling subtype were related to IL-6 receptor binding, glycoside metabolism, leukocyte aggregation, and varicosity. IL-6 was identified as a key factor regulating vascular remodeling in former studies (60). In this study, IL-6 receptor binding and varicosity were assigned to the same cluster in the similarity matrix of LAFSAA (**Figure 4A**), which indicated that IL-6 and vascular remodeling were at least statistically relevant in the samples of the discovery set, and potentially being functionally relevant in the process of EMT and progression of fibrosis. In former studies, M2 macrophage was believed to strongly promote vascular development (61). By combining the analytical results in this study and the physiological relationships between M2 macrophage and vascular development revealed in former studies, the high correlation between vascular remodeling and fibrosis in the pro-remodeling subtype is potentially due to the high proportion of M2 macrophage in immune cell population.

We proposed functional gene sets with subtype-specific correlations with EMT and fibrosis *via* LAFSAA. These findings suggested that the two subtypes had distinct fibrotic mechanisms. More research using larger clinical datasets or disease models representing the two subtypes of immune cell fractions are needed for further validating these correlations. More importantly, the different fibrotic mechanisms of the two subtypes heralds that patient stratification based on subtypes of immune cell fractions could potentially benefit anti-fibrotic therapies. Inhibiting the activities of subtype-specific fibrosis-related functional gene sets based on patients’ subtype affiliations could be a promising approach to improving therapeutic outcomes compared to standard therapeutic strategies, which should be investigated further with *in-vivo* and *in-vitro* tests.

### Patient stratification using immune subtyping for personalised therapy

The key benefit of precise stratification of patients is the ability to target fibrotic mechanisms associated with the immune cell fractions for effective treatment. The design of a strategy to predict subtypes of cardiac immune cell fractions in patients and assist in the development of treatment strategies is pivotal. However, current subtyping based on RNA profiling data from ventricular tissue samples cannot be directly applied in clinical settings. Therefore, we aimed to identify a panel of biomarkers correlated with subtypes of cardiac immune cell fractions that could be broadly applied for practical applications in personalized medical diagnosis.

We selected genes encoding secretory proteins for subtyping as potential biomarkers for assay-based screening. The Student’s t-test identified genes encoding secretory proteins that were expressed significantly different between the two subtypes (**Supplementary Figure 8**). The differentially expressed genes were screened with the LASSO regression (**Supplementary Figure 9**). We identified a unique panel of 32 genes with high performance in predicting patients’ subtypes of cardiac immune cell fractions using LASSO screening (**Supplementary Figure 10A**). To improve the robustness of the biomarker panel, we applied receiver operating characteristic (ROC) analysis to evaluate the classifying efficiency of genes that were not highly significantly different between the two subtypes according to the heatmap shown in **Supplementary Figure 10A**. We demonstrated that excluding HLA-DPA1 or KRT9 from the panel led to an increase in classifying efficiency in the validating set GSE57338, but not to a significant decrease in AUC in the discovery set, by successively excluding the selected genes from the panel and recording the changes in classifying efficiency quantified by AUC (**Supplementary Figure 10B**).

**Figure 5:**
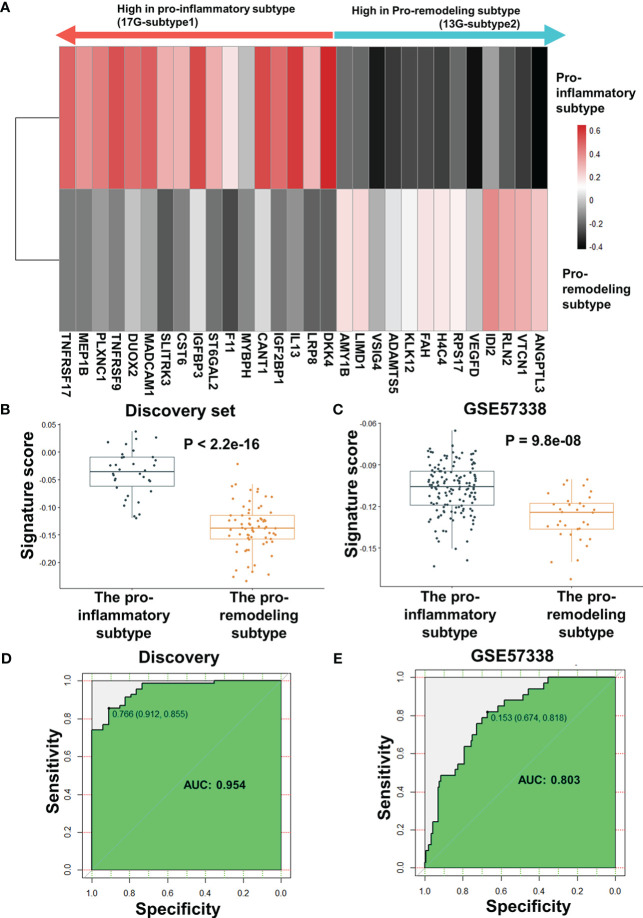
Performance evaluation for ImmunCard30 in predicting patients’ subtypes. **(A)** The average expression level of the 30 genes for subtype classification. The average z-score of each gene in the corresponding subtype was used to represent the expression level. **(B)** Signature scores of the two subtypes in the discovery set. **(C)** Signature scores of the two subtypes in the validating set GSE57338 were compared. **(D)** Receiver operating characteristic curves of the 30-gene biomarker panel was used to classify the two subtypes from the discovery set. **(E)** Receiver operating characteristic curves of the 30-gene biomarker panel was used to classify the two subtypes from the validating set GSE57338. A two-tailed Student’s t-test was used to determine significance. AUC, area under the curve.

After excluding HLA-DPA1 and KRT9, the AUC of the biomarker panel in validating set GSE57338 subtype classification increased from 0.79 to 0.802, while the AUC in the discovery set decreased from 0.958 to 0.954. (**Supplementary Figure 10B**). As a result of this finding, HLA-DPA1 and KRT9 were removed from the biomarker panel. Therefore, the optimal combination of biomarker panel consisted of 30 genes, termed ImmunCard30. In the ImmunCard30 panel, 17 genes were expressed significantly higher in the pro-inflammatory subtype (hereafter termed as 17G-subtype1), and 13 genes were expressed significantly higher in the pro-remodeling subtype (hereafter termed as 13G-subtype2) (**Figure 5A**)

To validate the performance of ImmunCard30, we applied the bi-directional *singscore* method to calculate signature scores for the clinical samples, where samples with a higher expression level of the 17G-subtype1 would be given higher scores, and a higher expression level of the 13G-subtype2 would lead to lower scores. We demonstrated that the samples from the pro-inflammatory subtype had significantly higher signature scores of the ImmunCard30 predictive panel than those from the pro-remodeling subtype, as confirmed in both the discovery and the validating set GSE57338 (**Figures 5B, C**). Signature scores calculated by the ImmunCard30 predictive panel achieved a high AUC of 0.954 in the discovery set (n = 103) and a relatively good predicting result in the validating set’s 177 samples (AUC = 0.803) (**Figures 5D, E**).

**Figure 6:**
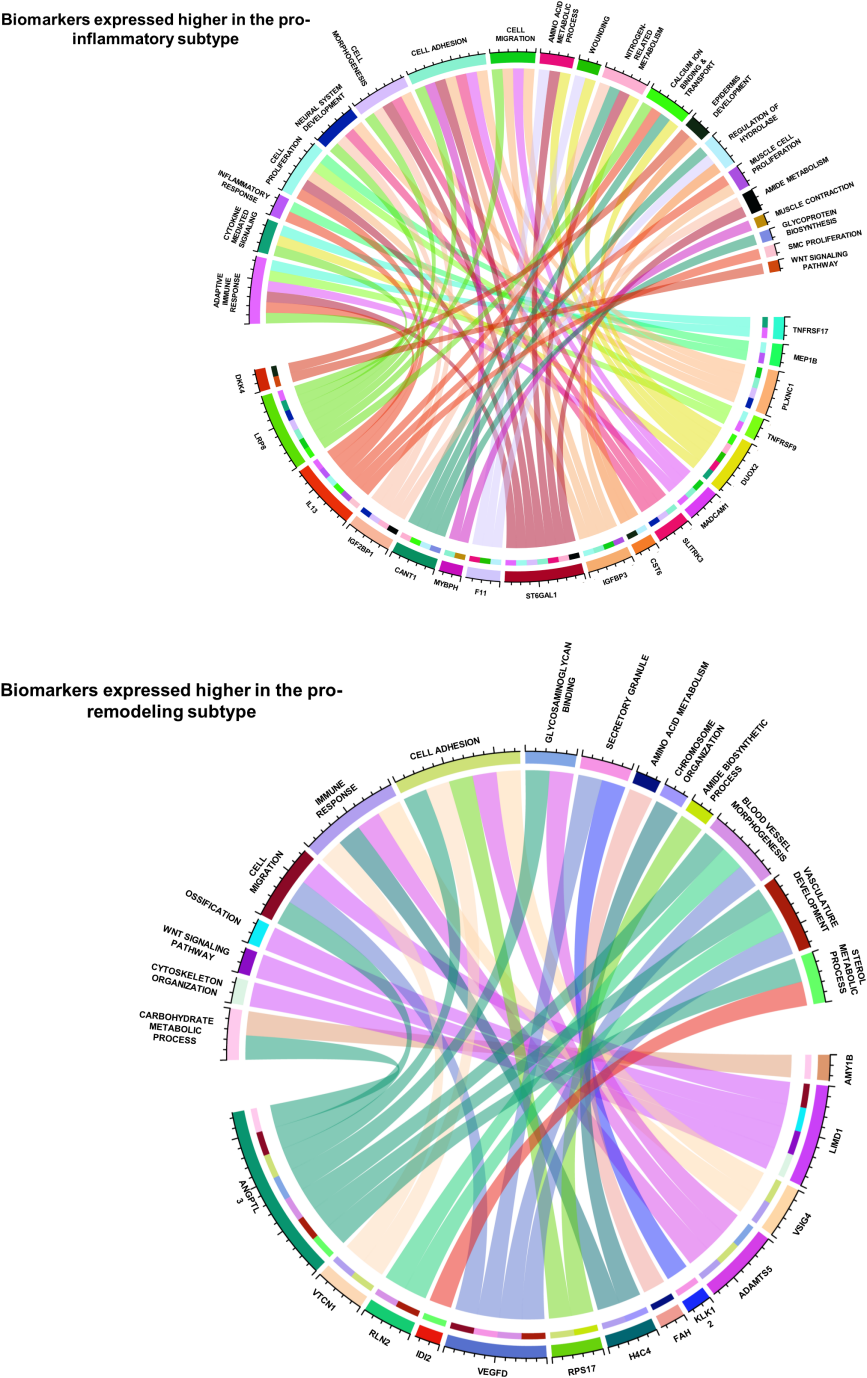
Functional annotations of the 30 biomarkers for predicting patients’ subtypes. The two circos plots demonstrated how the 30 biomarkers involved in pro-EMT and pro-fibrotic processes. Genes (bottom half of each circle) were linked to the functional annotations (upper half of each circle) that they were involved in.

The functional correlations between ImmunCard30 and fibrotic mechanisms could possibly be an approach to better understand the different fibrotic mechanisms in the two subtypes. MSigDB database contains over 10000 functional gene sets, with clear recordings of their functional annotations and gene lists. We screened the functional gene sets in the MSigDB database and identified those fibrosis-correlated functional gene sets (with signature scores significantly correlated with fibrosis, with a p-value of Spearman’s correlation< 0.05) that contained genes within the ImmunCard30. Functional annotations of these fibrosis-correlated functional gene sets from MSigDB were used to interpret the functions of genes from the ImmunCard30. According to the results, we found that genes within the ImmunCard30 panel were related to multiple pro-fibrosis processes or molecular components, such as adaptive immune response, cell migration and adhesion, neural system development, and vasculature development (**Figure 6**). In general, biomarkers in the 17G-subtype1 (ImmunCard30 genes that expressed significantly higher in the pro-inflammatory subtype) were more related to adaptive immune response, inflammation, and neural system development. In contrast, biomarkers in the 13G-subtype2 (ImmunCard30 genes that expressed significantly higher in the pro-remodeling subtype) were specifically related to vasculature development (**Figure 6**). Biomarkers from both subtypes were related to immune responses, cell migration, and cell adhesion (**Figure 6**).

By doing a literature review on functional records of genes in ImmunCard30, we found that most of the genes were having clear promoting or inhibiting effects on heart failure validated by *in-vitro*, *in-vivo* or clinical studies (**Supplementary Table 6**), and the remaining were found to statistically correlated with onset or progression of heart failure (**Supplementary Table 6**). Literature review also indicated that functions of 17G-subtype1 and 13G-subtype2 were related to features of their corresponding subtypes. Most genes from 17G-subtype1 directly participated in [for example, TNFRSF17, MEP1B, TNFRSF9, CST6, CANT1, IGF2BP1, IL13, LRP8 (62–71)] or at least were relevant to [for example, PLXNC1, DUOX2, SLITRK3, ST6GAL2, F11 (72–77)] inflammation and adaptive immune responses (**Supplementary Table 6**), which were major physiological features of the pro-inflammatory subtype. Meanwhile, genes from 13G-subtype2 were related to down-regulation of inflammatory responses [for example, LIMD1, VSIG4, ADAMTS5, VTCN1 (78–81)], blood vessel development [for example, VEGFD, RLN2, KLK12, and ANGPTL3 (82–86)], and maladaptive responses to reduced cardiac functions [for example, VEGFD, ANGPTL3, LIMD1, VSIG4, IDI2 (79, 83–88) (**Supplementary Table 6**), which were physiological features related to M2 macrophage (13). These records from *in-vitro, in-vivo* and clinical studies provide further evidence to prove the close relationship between ImmunCard30 and subtypes of immune cell fractions.

In order to evaluate the possibility of applying the ImmunCard30 panel in liquid biopsy, we went through the former literatures related to detection of ImmunCard30 proteins or genes’ expression in blood. According to the result, 27 of the members in ImmunCard30 have clear records indicating correlations between their levels in plasma/blood cells and cardiovascular healthy, while the remaining also have proven correlations with heart failure and being detectable in blood (**Supplementary Table 6**). Therefore, liquid biopsy based on blood samples would be a potential approach for detecting ImmunCard30. Further studies correlating ventricular immune cell fractions and blood levels of ImmunCard30 would be worth doing to validate the panel.

Overall, we presented a unique perspective based on immune subtyping using immune cell fractions calculated by CIBERSORTx. Subtype affiliation of immune cell fractions was potentially linked to diverse disease progression mechanisms, according to the analyses performed in this study. The accurate stratification of patient subtypes will greatly benefit the development and application of novel personalised therapeutic strategies. The ImmunCard30, which performed well on our discovery and validating sets (n = 280), is a promising biomarker panel that merits further investigation to determine its validity in predicting subtypes for patients and guiding individualised drug therapies.

## Discussion

Heart failure is a significant challenge to longevity with complex etiologies, and its progression is regulated by a heterogeneous immune cell fractions and complex feedbacks (1, 18, 26). The maladaptive structural changes in the heart during the progression of heart failure are generalized as cardiac remodeling. Cardiovascular fibrosis is a critical process in cardiac remodeling. Fibrosis was activated as a response to cardiac stress, such as cardiomyocyte death, pressure overload, and myocardial inflammation, to heal dead tissues and improve the physical strength of the myocardium (8). However, in chronic heart failure, fibrotic responses are constantly activated, resulting in excessive extracellular matrix accumulation and pathological fibrosis. The fibrotic tissues would stiffen the ventricle, reducing its compliance and diastolic function. Meanwhile, fibrotic tissues disrupt cardiac electrophysiology and limit nutrient supplies to cardiomyocytes, resulting in abnormal cardiac rhythm and cell death (8).

Because of the central role of fibrosis in the progression of cardiac remodeling, clinical studies have found that fibrosis is strongly associated with the prognosis of heart failure, regardless of the etiology of heart failure (4–6). Therefore, for the treatment of heart failure, it is pivotal to focus on central pathological processes such as cardiac fibrosis (89, 90). However, recent studies of anti-fibrosis therapies targeting fibrotic pathways yielded inconsistent or even worsened outcomes (19, 20), in part attributed to the highly heterogeneous immune cell fractions of fibrotic hearts. Although significant advancements in anti-fibrosis therapies were achieved, the heterogeneity of cell fractions in the cardiac tissue often led to varied treatment responses (13, 91). Precise stratification of patients and individualising therapies considering patients’ heterogeneous would be the future direction (91, 92). Numerous studies have demonstrated that the cardiac immune cell fractions is associated with the mechanism of fibrosis in patients (93, 94). Here, we developed a novel patient stratifying method based on the immune cell fractions and demonstrated the potential of this stratifying method in predicting patients’ fibrotic mechanisms and assisting the design of drug treatments.

We identified two subtypes of immune cell fractions for end-stage heart failure (pro-inflammatory and pro-remodeling subtypes) using ventricular tissue samples from 103 patients with heart failure, with significantly different levels of biomarkers related to adaptive immune responses. In both subtypes, EMT was identified as a potentially central fibrotic process. Endothelial biomarker analyses revealed strong correlations between endothelial cells and EMT. EndMT has been shown in previous studies to be a pro-fibrotic process in disease models (58). Further research on clinical cohorts could help determine whether EndMT was occurring and infer the potential contributions of EndMT to fibrosis in patients with end-stage heart failure. LAFSAA-based evaluation further identified 104 subtype-specific pro-EMT gene sets categorised into 8 functional clusters. We demonstrated that the 11 subtype-specific pro-fibrotic gene sets derived from the 8 functional clusters were related to inflammation, cell-cell adhesion, vasculature development, and responses to environmental stresses. A unique 30-gene biomarker panel was established to accurately stratify patient subtypes in both the discovery set (AUC = 0.954) and the validating set GSE57338 (AUC = 0.803).

Furthermore, subtype-specific pro-fibrotic mechanisms were highly correlated with representative immune cells of both subtypes, indicating casual relationships between the heterogeneous immune cell fractions and fibrotic mechanisms. We demonstrated that multiple processes related to regeneration, blood vessel development, and morphogenesis were correlated with fibrosis in the pro-remodeling subtype [for example, varicosity, smooth muscle cell matrix adhesion (**Figures 4A, B**)], while M2 macrophages (a distinct phenotype of the pro-remodeling subtype) was highly related to these processes (13, 61). In contrast, inflammation and adaptive immune responses, which were primarily modulated by T cells, B cells, and monocytes, were specifically correlated with fibrosis in the pro-inflammatory subtype. For example, defense response to virus, a typical adaptive immune response, was a subtype-specific fibrotic process in the pro-inflammatory subtype (**Figure 4B**). IL-13 and IL-4 secreted by CD4^+^ T cells (a distinct phenotype of the pro-inflammatory subtype) activate Janus kinase activity (95), and activation of Janus kinase was identified as a subtype-specific fibrotic process in the pro-inflammatory subtype (**Figure 4B**). These interplays between fibrosis and immune cells were also observed in early-stage heart failure (96–98). Therefore, although our current results were obtained based on end-stage patients, the panel should also be applicable to early-stage patients.

We envision that the 30-gene biomarker panel derived from ventricular tissue samples could provide a key reference for patient stratification, facilitating personalised therapeutic strategies based on drugs targeting fibrotic pathways (especially those related to subtype-specific pro-fibrotic gene sets, such as endothelin inhibitors, anti-TGFβ drugs, and anti-inflammation drugs), and reduce the adverse symptoms caused by drug treatments (such as inflammatory response (21)). Although ventricular tissue samples can be obtained with endomyocardial biopsy (99), the procedure increases mortality risk, cost and inconveniences the patient. Routine screening and assessment of patient subtypes would be more feasible through minimally invasive methods such as cardiovascular magnetic resonance (CMR), which is non-invasive and is the primary technology for stratifying patients with cardiac fibrosis (19, 100). However, CMR cannot provide information related to the intrinsic immunity of fibrotic lesions and is targeted to monitor fibrosis rather than guide individualised drug treatments targeting the fibrotic pathways. Here, our novel strategy for assessing subtypes of immune cell fractions is highly relevant to disease progression mechanisms. The ImmunCard30 is the first diagnostic biomarker panel reported to potentially reflect the heart’s immune cell fractions, assisting in assessing patients’ fibrotic mechanisms in the heart. The ImmunCard30 biomarker panel’s correlation with patients’ pathological conditions is expected to have significant implications for personalised medicine and, as such, warrants further clinical studies to validate, particularly validation using minimally invasive assessment methods such as liquid biopsy.

Clinical sampling of ventricular tissues is challenging due to the high risk of sampling operation. Immuncard30 is based on data from failing heart samples (n = 208) from various sources. However, there could still be bias in evaluating subtype-association of fibrotic mechanisms and the ImmunCard30 biomarker panel’s classifying efficiency. Our study provided new insights into the pathological correlations of cardiac immune cell fractions, which will be of great interest in developing personalised medicine. Further studies focusing on the *in vitro* and *in vivo* validation of the biomarker panel to validate drug responses and diagnostic efficiency are in progress to promote actual clinical utility. The progress made by this study support effort for the development of anti-fibrosis precision medicine for heart failure.

## Data availability statement

The original contributions presented in the study are included in the article/**Supplementary Material**. Further inquiries can be directed to the corresponding author.

## Author contributions

SZ conceived and designed the study, performed the study, analysed the data, and wrote the paper. BK conceived and designed the study, and wrote the paper. All authors contributed to the article and approved the submitted version.
