# Supplementary material for: Subtyping based on immune cell fractions reveal heterogeneity of cardiac fibrosis in end-stage heart failure
Source: Front Immunol. 2023 Feb 15;14:1053793. doi: 10.3389/fimmu.2023.1053793 (PMC9975711; doi:10.3389/fimmu.2023.1053793)
Supplement: Supplementary file 1 [file DataSheet_1.docx]

**Supplementary Figures**


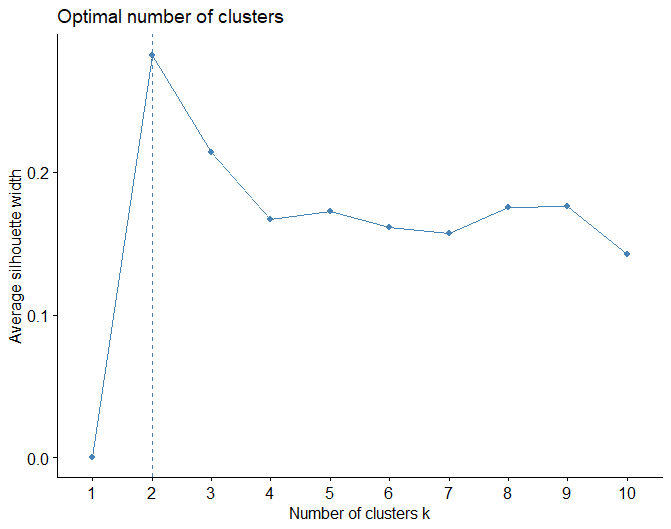


**Supplementary Figure 1 Predicting the optimal number of clusters.** The line graph shows the relationships between the number of clusters and average silhouette width. The optimal number of clusters should be the one that resulted in the highest average contour width, marked with a vertical dashed line.


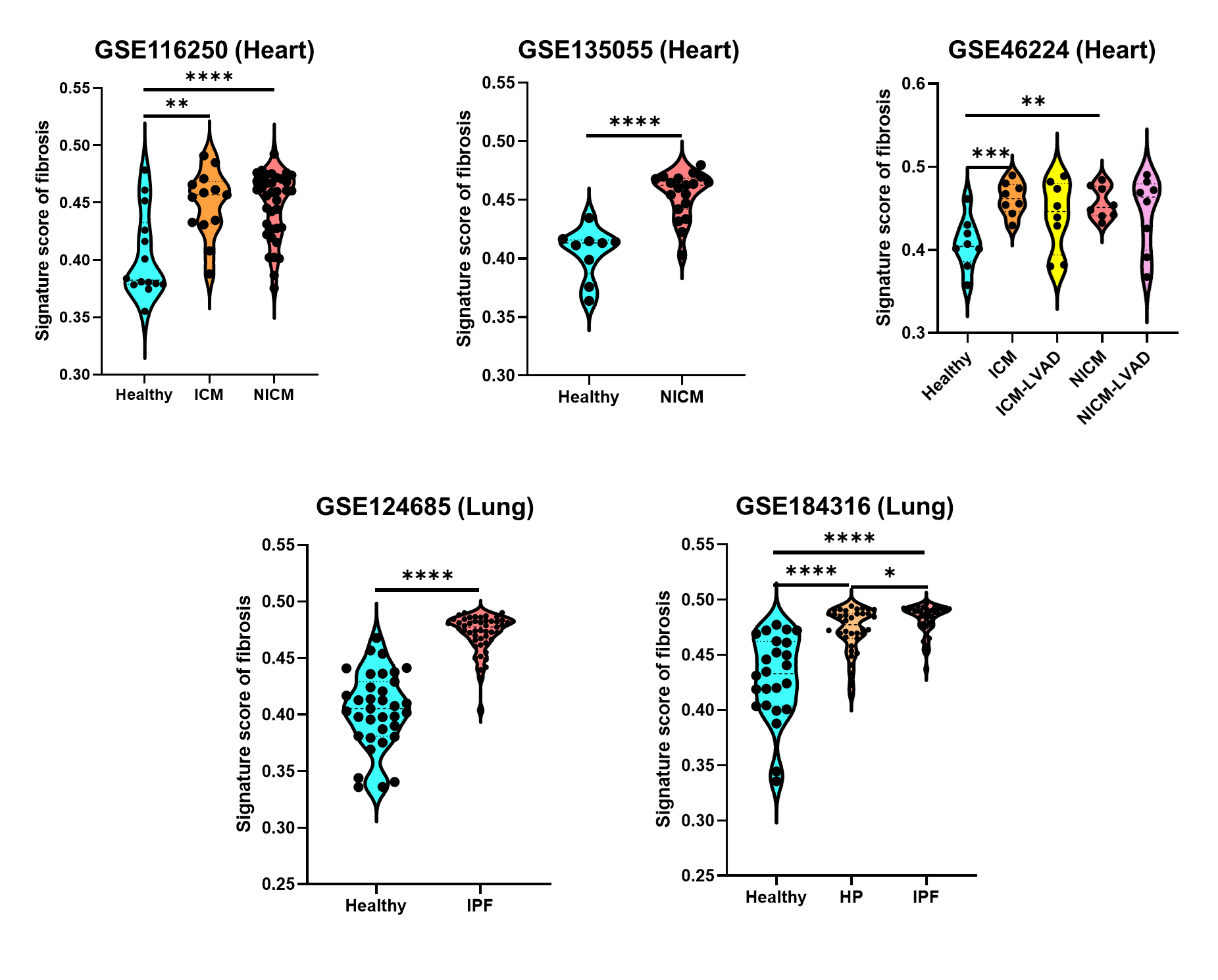


**Supplementary Figure 2 Validating fibrosis scores in multiple datasets from different organs.** The violin plots showed fibrosis scores in different fibrotic heart and lung clinical datasets. Ventricular samples from healthy hearts were compared to samples from ischemic cardiomyopathy (ICM), non-ischemic cardiomyopathy (NICM), and ICM or NICM patients treated with a left ventricular assist device (LVAD). Lung tissue from the healthy lungs was compared with tissue samples from patients with idiopathic pulmonary fibrosis (IPF) and hypersensitivity pneumonitis (HP). Dots in violin plots represent samples, while horizontal dash lines inside violin plots represent the median of the group. Statistical analysis was based on Student's t-test (P-value < 0.05: *; < 0.01: **; < 0.001: ***; < 0.0001: ****).


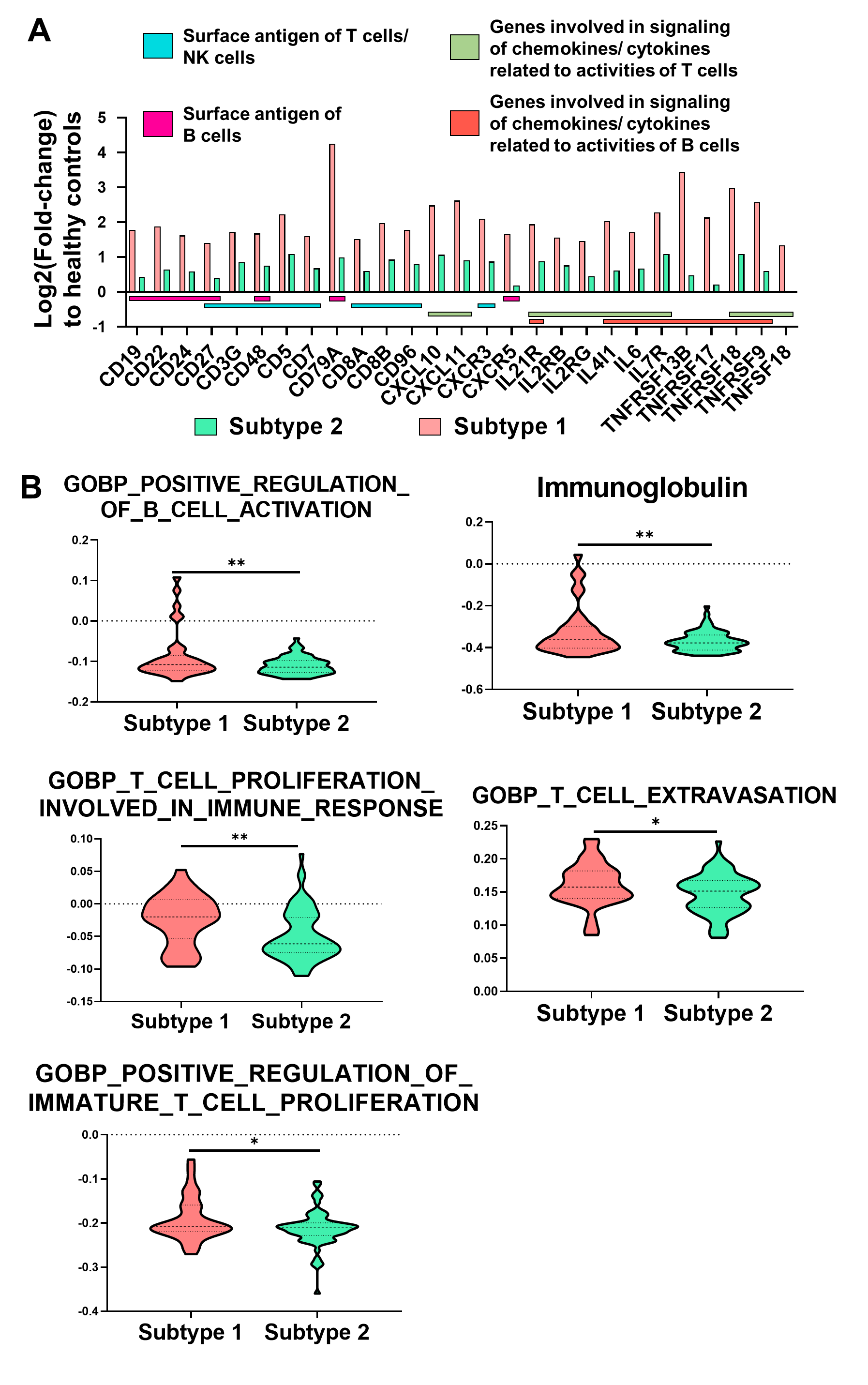


**Supplementary Figure 3 Quantitative analysis of the adaptive immune response between the two subtypes on the molecular level. (A)** Log2(fold-change) of selected genes in subtypes 1 and 2 versus healthy controls. Surface antigens of T cells and B cells, as well as genes involved in the signaling of chemokines and cytokines, are among the genes chosen. **(B)** The Y-axis of each subplot represents scores calculated using the "singscore" method. The Student's t-test was used for statistical analysis. (P-value < 0.05: *; < 0.01: **; < 0.001: ***; < 0.0001: ****).


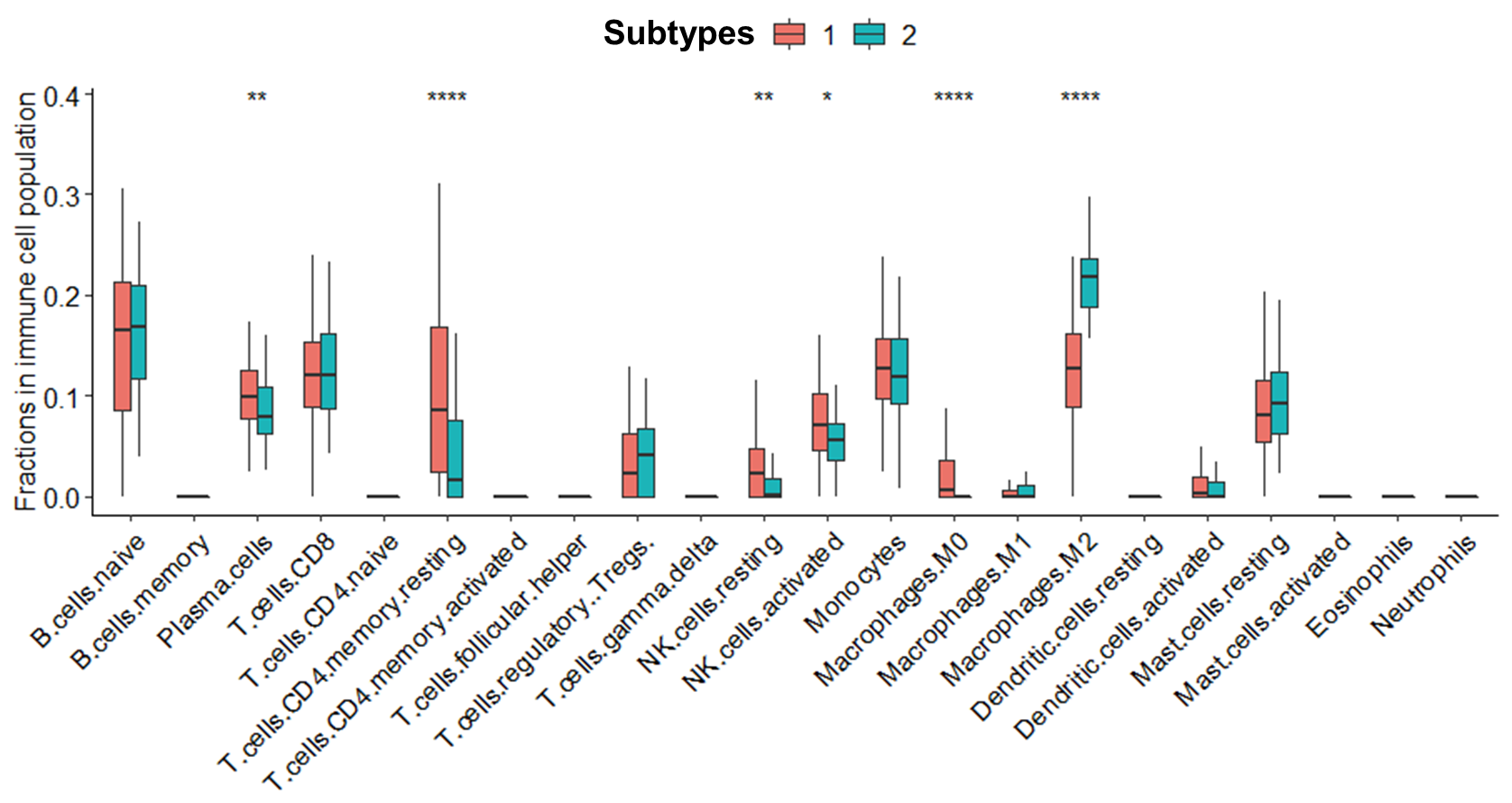


**Supplementary Figure 4 Immune cell fractions of the two subtypes in validating set GSE57338.** Boxplot showing the signature score of fibrosis in the two subtypes. Pink and cyan boxes, respectively, represented subtypes 1 and 2. The significance of the difference in score between the subtype 1 (the pro-inflammatory subtype) and the subtype 2 (the pro-remodeling subtype) was calculated by the two-tailed Student's t-test (t = 2.187, df = 101). For each boxplot: the center line represents the median, the box represents the interquartile range, and the whisker displays minimum to maximum. (P-value < 0.05: *; < 0.01: **; < 0.001: ***; < 0.0001: ****).

*
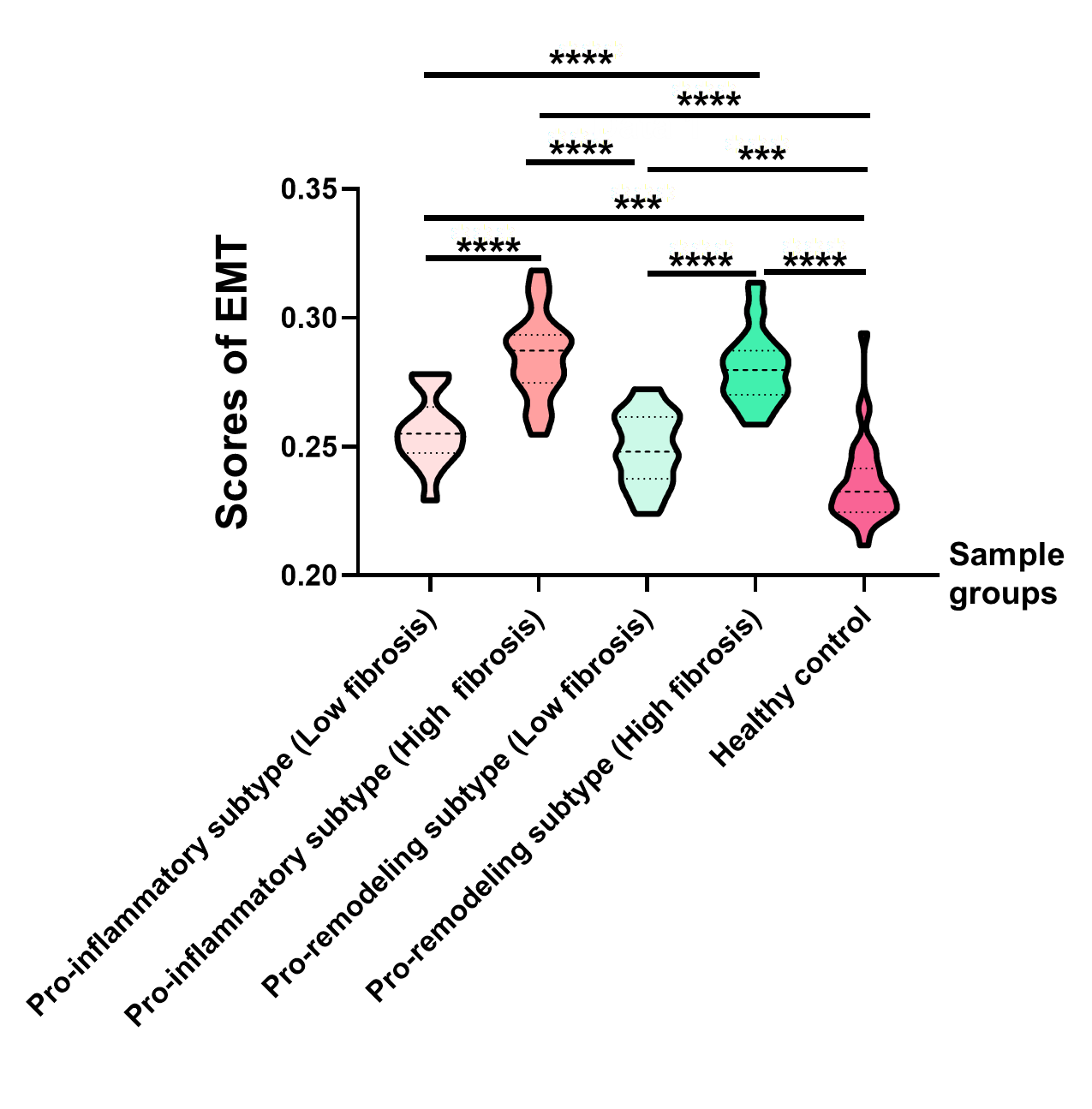
*

**Supplementary Figure 5 Comparison of EMT scores between different samples.** Groups included were high or low fibrosis groups from the two subtypes, and the healthy controls. Statistical analysis was based on Student's t-test (P-value < 0.05: *; < 0.01: **; < 0.001: ***; < 0.0001: ****).


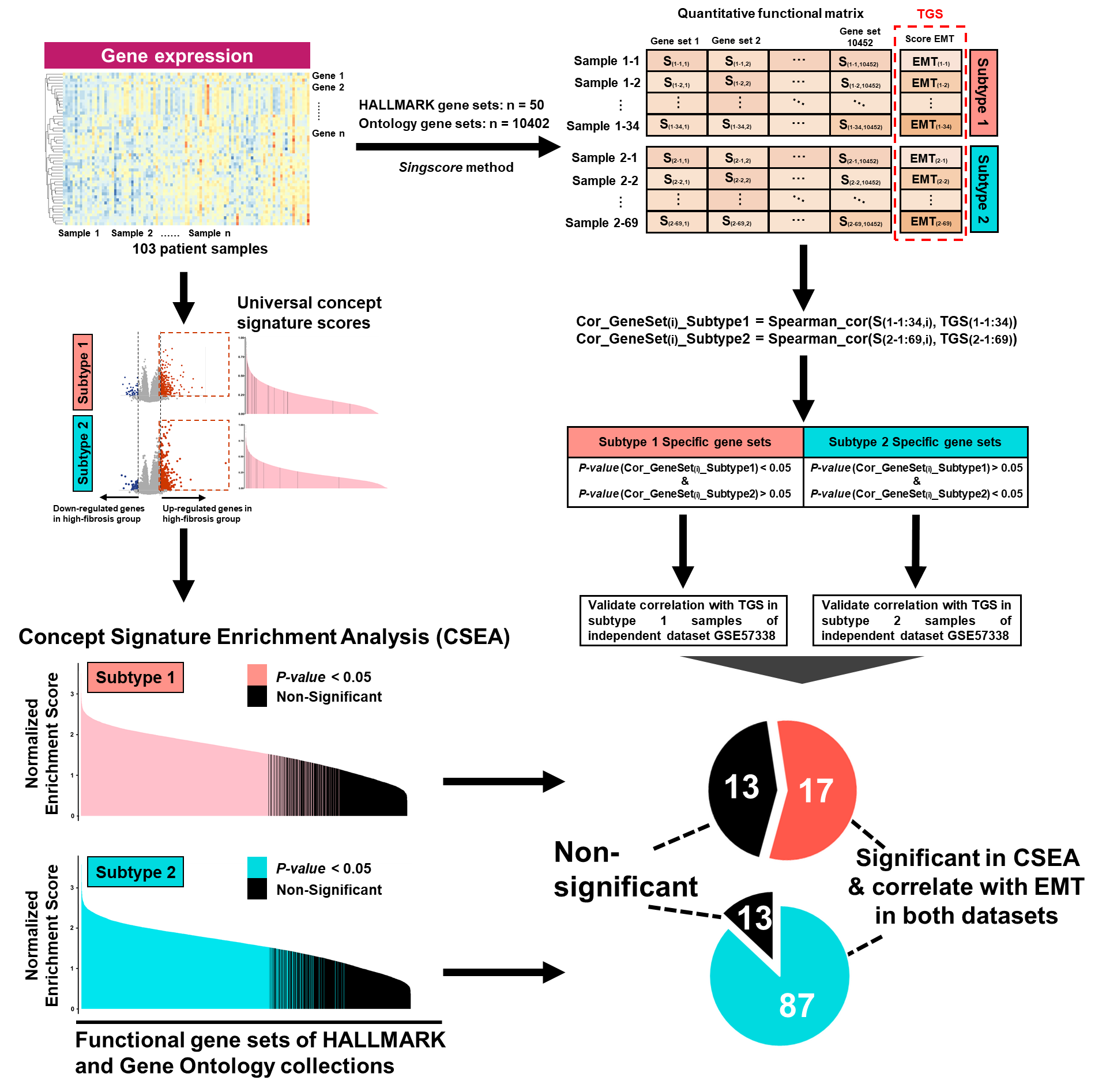


**Supplementary Figure 6 Workflow of the large-scale functional scoring and correlation analysis (LAFSAA).** Gene expression matrix of the discovery set (n = 103) was used to calculate signature scores of 10402 ontology gene sets and 50 HALLMARK gene sets retrieved from MSigDB. The epithelial-mesenchymal transition (EMT) score was used as the target gene set (TGS). Gene sets with Spearman correlation coefficients with EMT being significant (p-value < 0.05) in one subtype, meanwhile non-significant in the other subtype, were selected as subtype-specific pro-EMT gene sets. These gene sets were further validated in validating set GSE57338 to see whether these subtype-specific pro-EMT gene sets were significantly correlated with EMT in their corresponding subtypes in GSE57338. On the other hand, universal concept signature scores and concept signature enrichment analysis (CSEA) were applied to identify the functional gene sets enriched in the high-fibrosis group of each subtype. Those gene sets that passed both screening approaches were selected as subtype-specific pro-EMT gene sets.


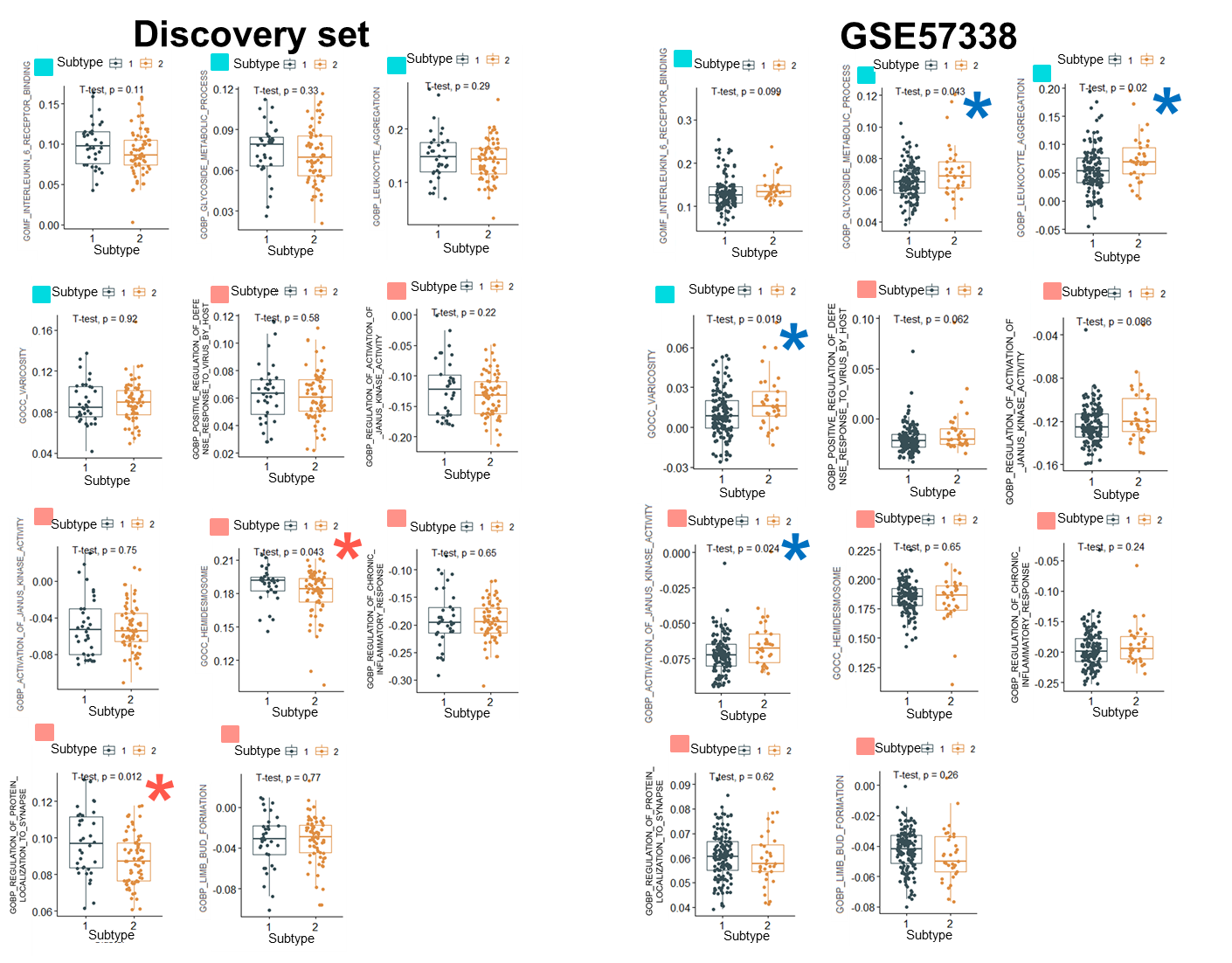


**Supplementary Figure 7 Signature scores of the subtype-specific pro-fibrotic gene sets in the two subtypes of the discovery set and the validating set GSE57338**. Gene sets specifically correlated with fibrosis in the pro-inflammatory subtype (Subtype 1) were marked with pink rectangles. In contrast, those gene sets specifically correlated with fibrosis in the pro-remodeling subtype (Subtype 2) were marked with turquoise rectangles.


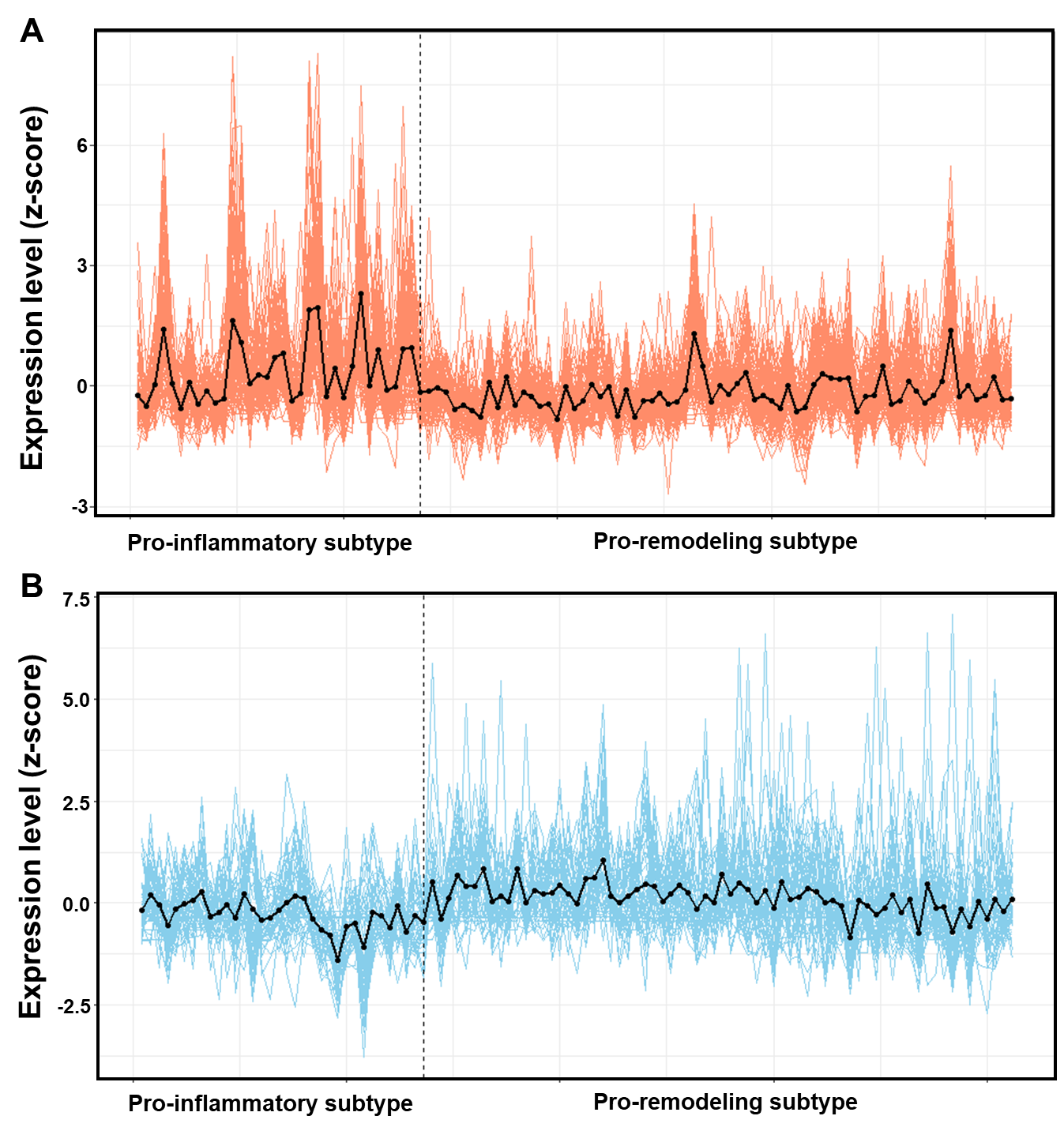


**Supplementary Figure 8 Genes expressed significantly different between the two subtypes. (A)** Line plot of genes expressed significantly higher in the pro-inflammatory subtype. **(B)** Line plot of genes expressed significantly higher in the pro-remodeling subtype.


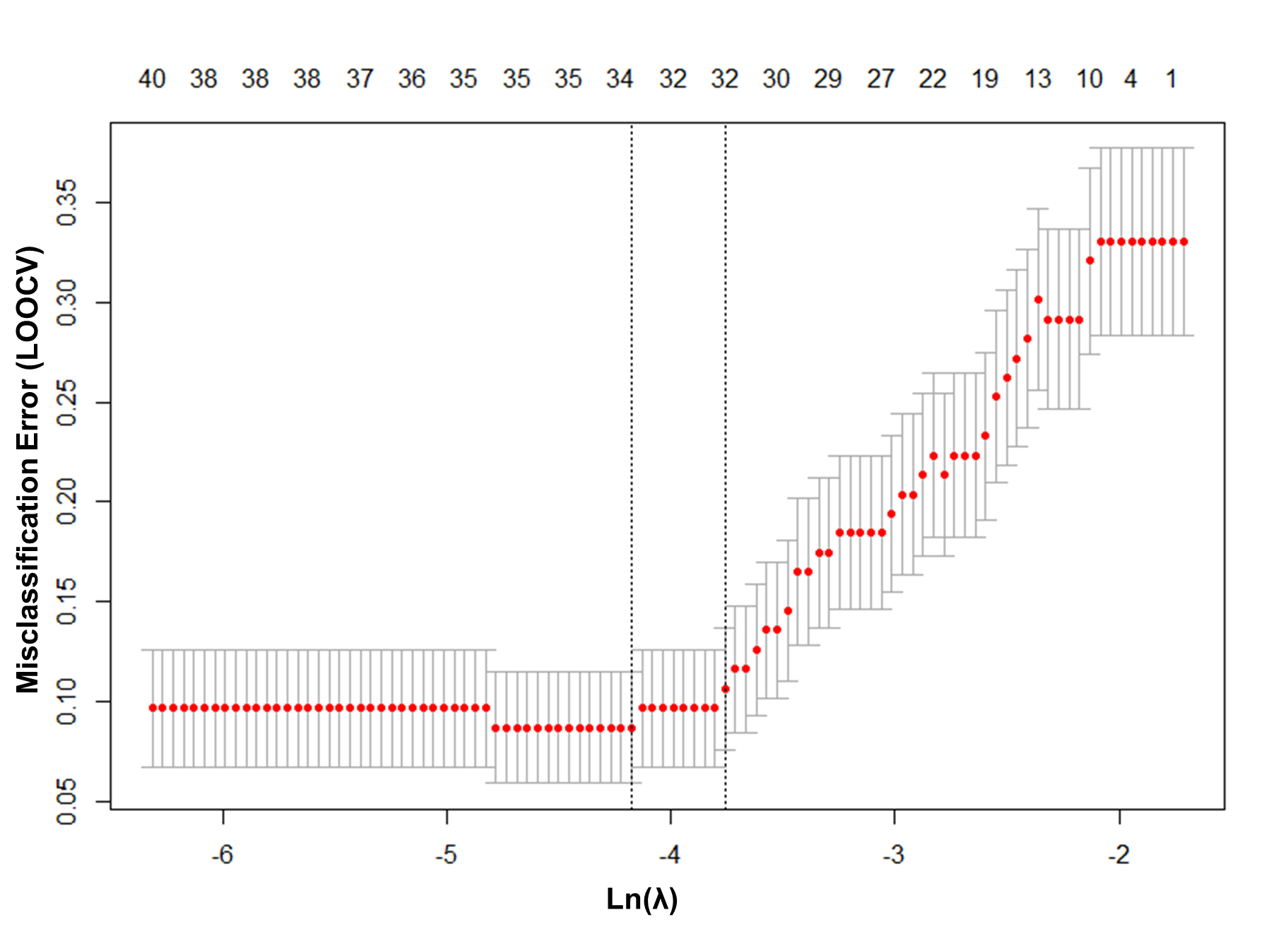


**Supplementary Figure 9 Screening differentially expressed genes between subtypes with least absolute shrinkage and selection operator (LASSO).** The plots show the feature selection based on LASSO regression. The Y-axis depicted the misclassification error of the model under leave-one-out cross-validation with different values ​​of λ. The two vertical dash lines denoted the ln (λ) value that resulted in the minimum misclassification error and the highest ln (λ) value that resulted in a misclassification error within 1 standard error of the minimum cross-validated error.


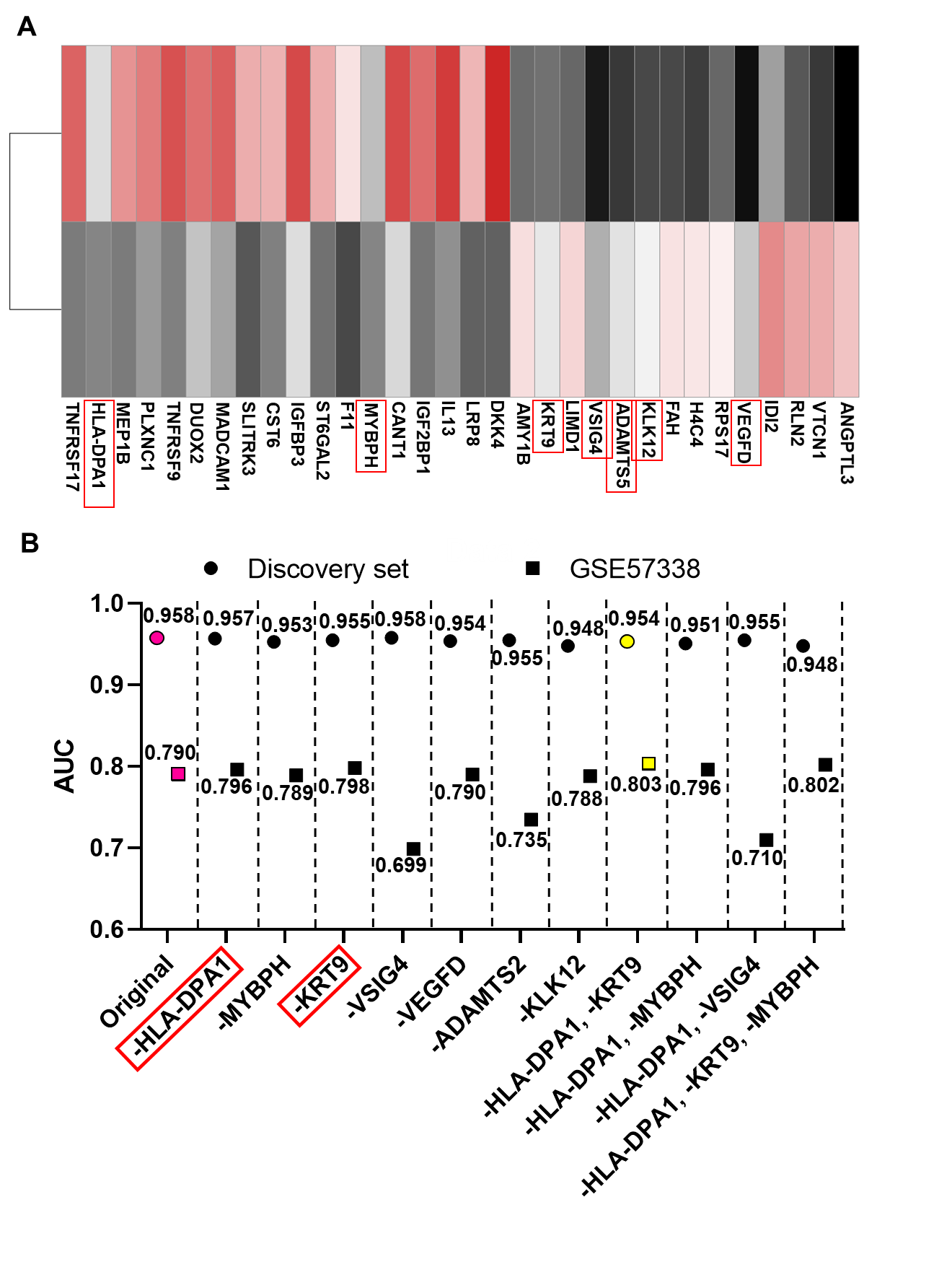


**Supplementary Figure 10 Receiver operating characteristic (ROC) analysis for evaluating classifying efficiency of genes. (A)** Heatmap showing the expression level (z-score) of the subtype-classifying biomarkers in the two subtypes. Genes marked by red rectangles were selected for ROC analysis . **(B)** Area under the ROC curve (AUC) of subtype classification in the discovery set and the GSE57338 validating set after excluding the selected genes from the panel one at a time. Each dot was labeled with the corresponding AUC value. In the two datasets, dots representing the AUC values of the original 32-gene panel were colored pink, while dots representing the AUC values of the gene panel after excluding HLA-DPA1 and KRT9 were colored yellow.
