# Supplementary material for: Subtyping based on immune cell fractions reveal heterogeneity of cardiac fibrosis in end-stage heart failure
Source: Front Immunol. 2023 Feb 15;14:1053793. doi: 10.3389/fimmu.2023.1053793 (PMC9975711; doi:10.3389/fimmu.2023.1053793)
Supplement: Supplementary file 2 [file DataSheet_2.docx]

**Supplementary Tables**

**Supplementary Table 1 Matrix of immune cell fractions in discovery set**

| Mixture | B cells naive | B cells memory | Plasma cells | T cells CD8 | T cells CD4 naive | T cells CD4 memory resting | T cells CD4 memory activated | T cells follicular helper | T cells regulatory (Tregs) | T cells gamma delta | NK cells resting | NK cells activated | Monocytes | Macrophages M0 | Macrophages M1 | Macrophages M2 | Dendritic cells resting | Dendritic cells activated | Mast cells resting | Mast cells activated | Eosinophils | Neutrophils |
| --- | --- | --- | --- | --- | --- | --- | --- | --- | --- | --- | --- | --- | --- | --- | --- | --- | --- | --- | --- | --- | --- | --- |
| DCM2 | 0 | 0.00086 | 0.002629 | 0.024222 | 0 | 0.177624 | 0 | 0 | 0 | 0.008929 | 0 | 0.00959 | 0.044591 | 0 | 0.031716 | 0.560613 | 0.027006 | 0 | 0.068861 | 0 | 0.030329 | 0.013028 |
| DCM3 | 0 | 0.003232 | 0.003386 | 0.056313 | 0 | 0.106209 | 0 | 0 | 0 | 0 | 0.058773 | 0.051286 | 0 | 0 | 0.020266 | 0.526797 | 0 | 0 | 0.135507 | 0 | 0.004475 | 0.033758 |
| DCM4 | 0 | 0.00196 | 0 | 0.078083 | 0 | 0.302554 | 0 | 0 | 0 | 0 | 0.027981 | 0.016544 | 0.115799 | 0.084474 | 0.011251 | 0.20226 | 0.005397 | 0 | 0.120503 | 0 | 0.033194 | 0 |
| DCM6 | 0 | 0.003256 | 0 | 0.012982 | 0 | 0.165719 | 0 | 0.002133 | 0.006325 | 0 | 0.077281 | 0 | 0.089906 | 0 | 0.03703 | 0.346586 | 0 | 0 | 0.258782 | 0 | 0 | 0 |
| DCM8 | 0 | 0.02118 | 0.005674 | 0.047444 | 0 | 0.145383 | 0 | 0 | 0 | 0 | 0.131577 | 0.046406 | 0.037195 | 0 | 0 | 0.299023 | 0 | 0 | 0.266118 | 0 | 0 | 0 |
| DCM11 | 0 | 0.025851 | 0.00635 | 0.005504 | 0 | 0.077652 | 0 | 0.025771 | 0 | 0 | 0.022251 | 0.014913 | 0.039333 | 0.053328 | 0.010607 | 0.36464 | 0.00981 | 0 | 0.277345 | 0 | 0.066642 | 0 |
| DCM12 | 0 | 0.009954 | 0 | 0.034015 | 0 | 0.142052 | 0 | 0.001659 | 0 | 0 | 0.059551 | 0 | 0.022969 | 0 | 0 | 0.467456 | 0 | 0 | 0.262344 | 0 | 0 | 0 |
| DCM13 | 0 | 0.005963 | 0.009472 | 0.122428 | 0 | 0.220414 | 0 | 0 | 0.003386 | 0 | 0.028212 | 0.090565 | 0.01041 | 0 | 0 | 0.395367 | 0 | 0 | 0.064873 | 0 | 0 | 0.04891 |
| DCM14 | 0 | 0 | 0.047869 | 0.019181 | 0 | 0.169404 | 0 | 0 | 0 | 0.016765 | 0 | 0.059397 | 0.067914 | 0 | 0.040038 | 0.437458 | 0 | 0 | 0.118269 | 0 | 0 | 0.023706 |
| DCM16 | 0 | 0.071632 | 0.024704 | 0.018281 | 0 | 0.275898 | 0 | 0 | 0 | 0 | 0.076111 | 0.052498 | 0.091877 | 0.095955 | 0.028177 | 0.040311 | 0.003748 | 0 | 0.211167 | 0 | 0.009641 | 0 |
| DCM17 | 0 | 0.016341 | 0.002791 | 0.026815 | 0 | 0.253693 | 0 | 0 | 0 | 0 | 0.133206 | 0 | 0.069024 | 0.004053 | 0 | 0.221872 | 0 | 0 | 0.151844 | 0 | 0 | 0.120362 |
| DCM18 | 0.058108 | 0 | 0.00022 | 0 | 0 | 0.182405 | 0.004445 | 0 | 0 | 0 | 0.015229 | 0.004833 | 0.007164 | 0 | 0.002082 | 0.449118 | 0 | 0.030347 | 0.120065 | 0 | 0.018505 | 0.107479 |
| DCM19 | 0 | 0.065121 | 0.018273 | 0.031838 | 0 | 0.394911 | 0 | 0.013928 | 7.50E-06 | 0 | 0.132417 | 0 | 0.097716 | 0.194417 | 0.011145 | 0 | 0.002099 | 0 | 0.023974 | 0 | 0.014153 | 0 |
| DCM20 | 0 | 0 | 0.004188 | 0.241133 | 0 | 0.127112 | 0 | 0 | 0 | 0 | 0.052537 | 0.071059 | 0.025813 | 0 | 0.033569 | 0.28503 | 0.007361 | 0 | 0.149099 | 0 | 0 | 0.003099 |
| DCM21 | 0 | 0.020646 | 0.02512 | 0.072243 | 0 | 0.159753 | 0 | 0 | 0 | 0 | 0.049727 | 0 | 0.073253 | 0 | 0.031514 | 0.331328 | 0.010307 | 0 | 0.166118 | 0 | 0.016075 | 0.043916 |
| DCM22 | 0 | 0.030475 | 0.011811 | 0.024204 | 0 | 0.36362 | 0 | 0 | 0 | 0 | 0.108703 | 0 | 0.076846 | 0 | 0 | 0.223272 | 0.000199 | 0 | 0.144548 | 0 | 0.016322 | 0 |
| DCM24 | 0 | 0.001587 | 0.018601 | 0.048911 | 0 | 0.089365 | 0 | 0.004634 | 0 | 0 | 0.020638 | 0.084733 | 0.103031 | 0 | 0.032932 | 0.38033 | 0 | 0 | 0.141519 | 0 | 0 | 0.07372 |
| DCM26 | 0 | 0 | 0.00666 | 0.046892 | 0 | 0.14329 | 0.032913 | 0 | 0 | 0 | 0.063387 | 0 | 0.029139 | 0 | 0.034604 | 0.423607 | 0.000284 | 0 | 0.089088 | 0 | 0.092564 | 0.037572 |
| DCM28 | 0 | 0.024326 | 0.006618 | 0.018362 | 0 | 0.232844 | 0 | 0.013111 | 0 | 0 | 0.046858 | 0.019467 | 0.05565 | 0.002254 | 0.006305 | 0.301449 | 0.01384 | 0 | 0.24639 | 0 | 0.012526 | 0 |
| DCM29 | 0 | 0 | 0.000117 | 0.095317 | 0 | 0.087354 | 0 | 0.030539 | 0 | 0 | 0.030001 | 0.178351 | 0.049984 | 0 | 0.04918 | 0.271777 | 0.000419 | 0.000586 | 0.206376 | 0 | 0 | 0 |
| DCM31 | 0 | 0.005328 | 0.001882 | 0.016047 | 0 | 0.151089 | 0 | 0 | 0.000585 | 0 | 0.139853 | 0 | 0.045851 | 0 | 0 | 0.51403 | 0 | 0.002443 | 0.071338 | 0 | 0 | 0.051555 |
| DCM32 | 0 | 0.019064 | 0.001966 | 0.034361 | 0 | 0.285522 | 0.054598 | 0 | 0 | 0 | 0.175113 | 0 | 0.02404 | 0.026821 | 0 | 0.018971 | 0 | 0.011241 | 0.08386 | 0 | 0 | 0.264444 |
| DCM33 | 0 | 0.039182 | 0.008638 | 0.053854 | 0 | 0.246218 | 0 | 0 | 0.050764 | 0 | 0.121501 | 0 | 0.062142 | 0.034714 | 0 | 0.182289 | 0.00665 | 0 | 0.194049 | 0 | 0 | 0 |
| DCM34 | 0 | 0 | 0.01974 | 0.108456 | 0 | 0.13953 | 0 | 0 | 0 | 0 | 0.035187 | 0 | 0.012281 | 0 | 0.039837 | 0.435939 | 0.002665 | 0 | 0.187011 | 0 | 0 | 0.019354 |
| DCM35 | 0 | 0.005569 | 0.001654 | 0.084248 | 0 | 0.263425 | 0 | 0 | 0 | 0 | 0.003179 | 0.004887 | 0.013279 | 0 | 0.009346 | 0.418571 | 0 | 0 | 0.18017 | 0 | 0 | 0.015673 |
| DCM38 | 0 | 0.001043 | 0.002778 | 0.119219 | 0 | 0.256017 | 0.004025 | 0.014474 | 0 | 0.011406 | 0.013244 | 0 | 0.036119 | 0 | 0.071239 | 0.38781 | 0.017402 | 0 | 0.046598 | 0 | 0 | 0.018627 |
| DCM39 | 0 | 0.039266 | 0.007901 | 0.085633 | 0 | 0.18125 | 0 | 0.003992 | 0.009908 | 0 | 0 | 0.022392 | 0.125223 | 0.142446 | 0 | 0.043772 | 0.004722 | 0 | 0.27301 | 0 | 0.060485 | 0 |
| DCM43 | 0.002855 | 0 | 0 | 0 | 0 | 0.251844 | 0 | 0 | 0 | 0 | 0.151027 | 0 | 0 | 0 | 0 | 0.399188 | 0 | 0.047892 | 0.087622 | 0 | 0.019998 | 0.039574 |
| DCM44 | 0 | 0.0189 | 0.007464 | 0 | 0 | 0.185486 | 0 | 0.011626 | 0 | 0 | 0.043287 | 0 | 0.056894 | 0.06238 | 0 | 0.256397 | 0 | 0 | 0.278677 | 0 | 0 | 0.078889 |
| DCM46 | 0.001472 | 0 | 0 | 0.132276 | 0 | 0.066161 | 0 | 0.004333 | 0.003516 | 0 | 0.012262 | 0.061727 | 0.049402 | 0 | 0.055715 | 0.342156 | 0.00015 | 0 | 0.155087 | 0 | 0 | 0.115742 |
| DCM72 | 0 | 0.051639 | 0.022532 | 0.045823 | 0 | 0.259685 | 0 | 0.003919 | 0 | 0 | 0.092361 | 0 | 0.066986 | 0.054514 | 0 | 0.273794 | 0 | 0 | 0.121577 | 0 | 0.007168 | 0 |
| DCM73 | 0 | 0.061668 | 0.021756 | 0.094048 | 0 | 0.215693 | 0 | 0 | 0.015884 | 0 | 0.099049 | 0.050014 | 0.135951 | 0.100419 | 0 | 0.032903 | 0.002848 | 0 | 0.129015 | 0 | 0.040752 | 0 |
| DCM74 | 0 | 0.006965 | 0.005667 | 0.123359 | 0 | 0.074024 | 0.043028 | 0 | 0 | 0 | 0.132082 | 0 | 0.007541 | 0.001598 | 0.025737 | 0.361475 | 0 | 0 | 0.135373 | 0 | 0 | 0.083151 |
| DCM75 | 0 | 0.005454 | 0.003273 | 0.057898 | 0 | 0.057856 | 0 | 0 | 0 | 0 | 0 | 0.080366 | 0.061125 | 0 | 0.020594 | 0.462543 | 0.015452 | 0 | 0.222063 | 0 | 0 | 0.013376 |
| DCM76 | 0 | 0.003508 | 0.00317 | 0.076678 | 0 | 0.216263 | 0.018439 | 0 | 0 | 0.056606 | 0 | 0.032826 | 0.033697 | 0 | 0.050044 | 0.303574 | 0 | 0.007749 | 0.131221 | 0 | 0.005459 | 0.060764 |
| DCM77 | 0 | 0.00074 | 0 | 0.146866 | 0 | 0.059248 | 0.008298 | 0.005503 | 0 | 0.058251 | 0.003977 | 0 | 0 | 0 | 0.029374 | 0.427485 | 0 | 0 | 0.15874 | 0 | 0.000764 | 0.100753 |
| DCM78 | 0 | 0.013074 | 0 | 0.070045 | 0 | 0.244826 | 0.002898 | 0.021746 | 0.000524 | 0 | 0 | 0 | 0.060691 | 0.097685 | 0.006208 | 0.214668 | 0 | 0 | 0.218291 | 0 | 0.049345 | 0 |
| ICM47 | 0.00173 | 0 | 0.005608 | 0.067598 | 0 | 0.231929 | 0 | 0 | 0 | 0.055896 | 0 | 0.034907 | 0.041397 | 0 | 0.099583 | 0.29066 | 0.003349 | 0 | 0.05849 | 0 | 0 | 0.108853 |
| ICM49 | 0 | 0 | 0.001806 | 0 | 0 | 0.169103 | 0 | 0 | 0.05504 | 0 | 0.020479 | 0 | 0.039216 | 0.086743 | 0.049554 | 0.314204 | 0 | 0 | 0.234987 | 0 | 0 | 0.028869 |
| ICM50 | 0 | 0 | 0.015775 | 0.042654 | 0 | 0.205867 | 0.038518 | 0 | 0 | 0 | 0 | 0.023935 | 0.009566 | 0.094335 | 0.306402 | 0.166354 | 0 | 0 | 0 | 0.086409 | 0.010186 | 0 |
| ICM52 | 0 | 0 | 0.069809 | 0.170042 | 0 | 0.172865 | 0 | 0 | 0 | 0.007277 | 0.027696 | 0 | 0 | 0 | 0.004377 | 0.317966 | 0 | 0.008942 | 0.057584 | 0 | 0.048694 | 0.114749 |
| ICM53 | 0 | 0.038485 | 0.010955 | 0.060185 | 0 | 0.150386 | 0 | 0.002339 | 0.016526 | 0 | 0.114877 | 0 | 0.026426 | 0 | 0 | 0.34273 | 0 | 0 | 0.208245 | 0 | 0 | 0.028846 |
| ICM54 | 0 | 0.018158 | 0.002581 | 0.062951 | 0 | 0.320228 | 0.004003 | 0.021733 | 0.016179 | 0 | 0 | 0.026468 | 0.003907 | 0.043645 | 0.160008 | 0.25804 | 0.028222 | 0 | 0.033877 | 0 | 0 | 0 |
| ICM55 | 0 | 0.004369 | 0 | 0 | 0 | 0.1172 | 0 | 0 | 0.013331 | 0 | 0.089247 | 0 | 0.065724 | 0 | 0.005145 | 0.398886 | 0.000485 | 0 | 0.305524 | 0 | 8.97E-05 | 0 |
| ICM56 | 0 | 0.020587 | 0.010008 | 0 | 0 | 0.29615 | 0 | 0 | 0.000911 | 0 | 0 | 0 | 0.044392 | 0 | 0 | 0.389961 | 0.00134 | 0 | 0.13892 | 0 | 0.050859 | 0.046874 |
| ICM58 | 0 | 0.005442 | 0.022548 | 0.047603 | 0 | 0.167411 | 0 | 0 | 0 | 0 | 0.010837 | 0.017672 | 0.01419 | 0 | 0.031971 | 0.462945 | 0 | 0 | 0.136194 | 0 | 0.030461 | 0.052727 |
| ICM59 | 0 | 0.008413 | 0.005226 | 0.032181 | 0 | 0.228938 | 0 | 0 | 0 | 0 | 0.037038 | 0 | 0.035258 | 0 | 0.00742 | 0.436958 | 0 | 0 | 0.134532 | 0 | 0.03147 | 0.042566 |
| ICM61 | 0 | 0.006748 | 0.000742 | 0 | 0 | 0.186965 | 0 | 0.043557 | 0 | 0 | 0.003742 | 0.007398 | 0.01139 | 0 | 0.108519 | 0.298617 | 0 | 0 | 0.130985 | 0 | 0.058888 | 0.142447 |
| ICM63 | 0 | 0.003048 | 0.000863 | 0.031589 | 0 | 0.139666 | 0.033624 | 0.009961 | 0.034436 | 0 | 0.111283 | 0 | 0.08671 | 0.232341 | 0 | 0.261229 | 0 | 0 | 0.013551 | 0 | 0 | 0.041698 |
| ICM64 | 0 | 0.011389 | 0 | 0.003665 | 0 | 0.245737 | 0 | 0 | 0 | 0 | 0.149418 | 0.004521 | 0.012694 | 0 | 0 | 0.552179 | 0 | 0.008746 | 0 | 0 | 0.011652 | 0 |
| SRR9856187 | 0 | 0.01296 | 0.025119 | 0.065553 | 0 | 0.377556 | 0 | 0 | 0 | 0 | 0 | 0.088126 | 0.041955 | 0.026346 | 0.04097 | 0.085882 | 0 | 0 | 0.235533 | 0 | 0 | 0 |
| SRR9856188 | 0 | 0.001037 | 0.016712 | 0.192444 | 0 | 0.202716 | 0 | 0 | 0 | 0 | 0 | 0.116916 | 0.035988 | 0 | 0.04341 | 0.268264 | 0.000978 | 0 | 0.121471 | 0 | 6.45E-05 | 0 |
| SRR9856189 | 0 | 0.013172 | 0.045085 | 0.141329 | 0 | 0.039258 | 0 | 0.03146 | 0 | 0.03967 | 0 | 0.08404 | 0 | 0 | 0.072337 | 0.335869 | 0.021518 | 0 | 0.159299 | 0 | 0.016963 | 0 |
| SRR9856190 | 0 | 0.00321 | 0.010532 | 0.169708 | 0 | 0.163866 | 0.02712 | 0 | 0 | 0 | 0 | 0.101213 | 0.022715 | 0 | 0.031346 | 0.242969 | 0 | 0 | 0.138582 | 0 | 0.03661 | 0.052129 |
| SRR9856191 | 0 | 0.064601 | 0.025755 | 0.137488 | 0 | 0.265657 | 0 | 0.00764 | 0 | 0 | 0.04995 | 0.030974 | 0.081553 | 0.058688 | 0.040814 | 0.022462 | 0.013833 | 0 | 0.181477 | 0 | 0.019107 | 0 |
| SRR9856192 | 0 | 0 | 0.006415 | 0.071616 | 0 | 0.202201 | 0 | 0 | 0 | 0.009079 | 0.123434 | 0.012348 | 0.021971 | 0 | 0.052787 | 0.277885 | 0 | 0.008657 | 0.213605 | 0 | 0 | 0 |
| SRR9856193 | 0 | 0.029106 | 0 | 0.165424 | 0 | 0.127037 | 0.037683 | 0.012108 | 0 | 0 | 0.077827 | 0.050896 | 0.006981 | 0 | 0.023742 | 0.294703 | 0 | 0 | 0.11163 | 0 | 0.024602 | 0.03826 |
| SRR9856194 | 0 | 0.019026 | 0.058539 | 0.204031 | 0 | 0.225627 | 0.020719 | 0 | 0 | 0.000567 | 0 | 0.035897 | 0.010688 | 0 | 0.049735 | 0.223391 | 0.008797 | 0.023621 | 0.117401 | 0 | 0 | 0.00196 |
| SRR9856195 | 0 | 0.001384 | 0 | 0.172217 | 0 | 0.151869 | 0 | 0.014166 | 0 | 0.015689 | 0 | 0.127243 | 0.005432 | 0 | 0.028981 | 0.252619 | 0 | 0.001219 | 0.16299 | 0 | 0.062388 | 0.003803 |
| SRR9856196 | 0 | 0.026533 | 0 | 0.169214 | 0 | 0.147 | 0.014086 | 0.000463 | 0 | 0 | 0.010487 | 0.067553 | 0.013438 | 0 | 0.059226 | 0.321147 | 0.036143 | 0 | 0.128245 | 0 | 0 | 0.006464 |
| SRR9856197 | 0 | 0.008739 | 0.002359 | 0.095744 | 0 | 0.197705 | 0 | 0 | 0 | 0 | 0 | 0.013088 | 0 | 0 | 0.031661 | 0.363452 | 0.00382 | 0 | 0.259575 | 0 | 0.020644 | 0.003213 |
| SRR9856198 | 0 | 0.128812 | 0.033344 | 0.07404 | 0 | 0.295647 | 0 | 0.006984 | 0 | 0 | 0 | 0.0329 | 0.104227 | 0.066826 | 0.01959 | 0 | 0.006405 | 0 | 0.231226 | 0 | 0 | 0 |
| SRR9856199 | 0 | 0.059718 | 0.0046 | 0.020522 | 0 | 0.353119 | 0 | 0.029892 | 0 | 0 | 0.104713 | 0.000755 | 0.08123 | 0.064642 | 0.021705 | 0.045575 | 0.003152 | 0 | 0.183633 | 0 | 0.026742 | 0 |
| SRR9856200 | 0 | 0.087406 | 0.027852 | 0.017295 | 0 | 0.21177 | 0 | 0 | 0 | 0 | 0.12949 | 0.005879 | 0.104931 | 0.090768 | 0.054463 | 0.039549 | 0.022636 | 0.014126 | 0.163189 | 0 | 0.030647 | 0 |
| SRR9856201 | 0 | 0 | 0.002602 | 0.226189 | 0 | 0.261837 | 0.008881 | 0.002999 | 0 | 0.013547 | 0 | 0.082981 | 0.000537 | 0 | 0.043942 | 0.279803 | 0 | 0.001352 | 0.074521 | 0 | 0.000808 | 0 |
| SRR9856202 | 0 | 0.009735 | 0.009379 | 0.159565 | 0 | 0.156293 | 0.017886 | 0 | 0 | 0 | 0 | 0.045344 | 0.011261 | 0 | 0.04456 | 0.381235 | 0 | 0.002034 | 0.134268 | 0 | 0 | 0.02844 |
| SRR9856203 | 0 | 0 | 0.022397 | 0.09592 | 0 | 0.142636 | 0 | 0 | 0.011553 | 0 | 0 | 0.103894 | 0.027573 | 0 | 0.012291 | 0.367568 | 0.005434 | 0.00025 | 0.199272 | 0 | 0 | 0.011213 |
| SRR9856204 | 0.003794 | 0 | 0 | 0.104398 | 0 | 0.136676 | 0 | 0 | 0 | 0 | 0 | 0.056737 | 0.05009 | 0 | 0.043579 | 0.452515 | 0.014174 | 0 | 0.138037 | 0 | 0 | 0 |
| SRR9856205 | 0.015071 | 0 | 0.014532 | 0.229405 | 0 | 0.161907 | 0 | 0.01185 | 0 | 0.007152 | 0 | 0.089148 | 0.014913 | 0 | 0.078068 | 0.222315 | 0.014918 | 0 | 0.140722 | 0 | 0 | 0 |
| SRR9856206 | 0 | 0.073092 | 0.026604 | 0.222888 | 0 | 0.247108 | 0 | 0 | 0 | 0.099685 | 0 | 0.022816 | 0 | 0.052462 | 0.01298 | 0.094816 | 0.006407 | 0 | 0.141141 | 0 | 0 | 0 |
| SRR9856207 | 0.000151 | 0.002896 | 0.023734 | 0.190353 | 0 | 0.180652 | 0 | 0.000572 | 0.035813 | 0.018604 | 0 | 0.024485 | 0 | 0.263423 | 0.071049 | 0.16431 | 0.005524 | 0 | 0.018433 | 0 | 0 | 0 |
| ICM.LVAD1 | 0 | 0.069306 | 0.168384 | 0.187768 | 0 | 0.282351 | 0 | 0 | 0 | 0 | 0 | 0 | 0.043586 | 0.058517 | 0.060004 | 0.069044 | 0 | 0 | 0.05531 | 0 | 0.00573 | 0 |
| ICM.LVAD2 | 0 | 0.084785 | 0.031053 | 0 | 0 | 0.208894 | 0 | 0 | 0 | 0 | 0.058198 | 0 | 0.021751 | 0.01137 | 0.014017 | 0.295797 | 0 | 0 | 0.142607 | 0 | 0.045324 | 0.086206 |
| ICM.LVAD3 | 0.006854 | 0 | 0.011322 | 0.066736 | 0 | 0.198833 | 0 | 0 | 0 | 0 | 0.011567 | 0 | 0 | 0 | 0.009412 | 0.410485 | 0 | 0 | 0.251965 | 0 | 0.032825 | 0 |
| ICM.LVAD4 | 0 | 0.012138 | 0.014089 | 0.025677 | 0.049542 | 0 | 0 | 0.017691 | 0 | 0 | 0.013796 | 0.019402 | 0 | 0 | 0.045502 | 0.481151 | 0 | 0 | 0.185866 | 0 | 0.020433 | 0.114714 |
| ICM.LVAD5 | 0 | 0.035563 | 0.008601 | 0.052619 | 0 | 0.077426 | 0 | 0.076509 | 0 | 0.012513 | 0 | 0.077506 | 0.071606 | 0 | 0.289134 | 0.154024 | 0.008366 | 0 | 0.123379 | 0 | 0 | 0.012754 |
| ICM.LVAD6 | 0 | 0.004257 | 0.001524 | 0.072507 | 0 | 0.127862 | 0 | 0.039884 | 0 | 0 | 0.009962 | 0.063553 | 0.05166 | 0 | 0.033232 | 0.243097 | 0 | 0.003253 | 0.106691 | 0.059854 | 0.037718 | 0.144945 |
| ICM.LVAD7 | 0 | 0.03401 | 0.10935 | 0.043468 | 0 | 0.221786 | 0 | 0 | 0 | 0 | 0.14492 | 0 | 0.027546 | 0 | 0.046536 | 0.194271 | 0 | 0 | 0.134351 | 0 | 0 | 0.043763 |
| ICM.LVAD8 | 0 | 0.01258 | 0.022943 | 0.01715 | 0 | 0.212425 | 0 | 0.003648 | 0 | 0 | 0 | 0.111177 | 0.078785 | 0.040528 | 0.046886 | 0.236169 | 0.004955 | 0 | 0.200892 | 0 | 0 | 0.011863 |
| NICM.LVAD1 | 0.077492 | 0.173476 | 0 | 0.126565 | 0 | 0.280717 | 0 | 0 | 0 | 0 | 0.024321 | 0.015397 | 0.015417 | 0.00723 | 0.029949 | 0.151705 | 0 | 0 | 0.055451 | 0 | 0 | 0.04228 |
| NICM.LVAD2 | 0 | 0 | 0.020697 | 0.003814 | 0 | 0.210632 | 0 | 0 | 0 | 0 | 0.072895 | 0 | 0.032514 | 0 | 0.062324 | 0.361361 | 0 | 0 | 0.189158 | 0 | 0.011195 | 0.035411 |
| NICM.LVAD3 | 0 | 0.011196 | 0.073873 | 0 | 0 | 0.155499 | 0 | 0 | 0 | 0 | 0.014681 | 0.016756 | 0.00159 | 0 | 0.041588 | 0.234311 | 0 | 0 | 0.410308 | 0 | 0 | 0.0402 |
| NICM.LVAD4 | 0 | 0.012305 | 0.007876 | 0.037756 | 0 | 0.118994 | 0 | 0 | 0 | 0 | 0 | 0.0422 | 0.007293 | 0 | 0.024049 | 0.438597 | 0 | 0.011803 | 0.218025 | 0 | 0.030665 | 0.050439 |
| NICM.LVAD5 | 0 | 0.066224 | 0.02947 | 0.045108 | 0 | 0.195426 | 0 | 0 | 0.01747 | 0 | 0.045837 | 0.005053 | 0.05488 | 0 | 0.013947 | 0.374702 | 0 | 0 | 0.088426 | 0 | 0 | 0.063458 |
| NICM.LVAD6 | 0 | 0.038516 | 0.031934 | 0.058544 | 0 | 0.177613 | 0 | 0.018598 | 0 | 0 | 0.007388 | 0 | 0.027736 | 0 | 0.013003 | 0.353299 | 0.015442 | 0.000409 | 0.240383 | 0 | 0.014251 | 0.002883 |
| NICM.LVAD7 | 0 | 0.012211 | 0.002336 | 0.077969 | 0 | 0.371059 | 0 | 0.021281 | 0 | 0 | 0.074683 | 0 | 0.060918 | 0.003219 | 0.015661 | 0.250353 | 0 | 0 | 0.014544 | 0 | 0.0606 | 0.035165 |
| NICM.LVAD8 | 0 | 0.077166 | 0 | 0.004227 | 0 | 0.149704 | 0 | 0 | 0 | 0 | 0.071249 | 0 | 0.020402 | 0 | 0.016706 | 0.43895 | 0 | 0.005293 | 0.084284 | 0 | 0.018264 | 0.113755 |
| ICM1 | 0 | 0.026879 | 0.026869 | 0.009608 | 0 | 0.240392 | 0 | 0 | 0.001067 | 0 | 0.124927 | 0.036528 | 0.098048 | 0.115893 | 0.025513 | 0.267659 | 0.003668 | 0 | 0 | 0 | 0.022947 | 0 |
| ICM2 | 0 | 0.012554 | 0.01099 | 0.013495 | 0 | 0.288551 | 0 | 0.005406 | 0.002625 | 0 | 0.052767 | 0.069867 | 0.125809 | 0.187488 | 0.002151 | 0.00621 | 0 | 0 | 0.066464 | 0 | 0.06236 | 0.093262 |
| ICM3 | 0 | 0.107437 | 0.014758 | 0.18877 | 0 | 0.24825 | 0 | 0 | 0.048216 | 0 | 0.003428 | 0 | 0.09616 | 0.020231 | 0.062688 | 0.059084 | 0.009267 | 0 | 0.141712 | 0 | 0 | 0 |
| ICM4 | 0 | 0.107117 | 0.026663 | 0.106005 | 0.036679 | 0 | 0 | 0.017871 | 0 | 0 | 0.114958 | 0 | 0.081319 | 0 | 0.011476 | 0.408303 | 0 | 0 | 0.089609 | 0 | 0 | 0 |
| ICM5 | 0 | 0.003604 | 0.001142 | 0.009535 | 0 | 0.197534 | 0 | 0.014248 | 0 | 0.013569 | 0 | 0.049722 | 0.072699 | 0 | 0.061136 | 0.443626 | 0 | 0.000869 | 0.112506 | 0 | 0 | 0.01981 |
| ICM6 | 0 | 0.082864 | 0.022219 | 0.069123 | 0 | 0.227518 | 0 | 0.002902 | 0 | 0 | 0 | 0 | 0.055856 | 0.118208 | 0.032758 | 0.111671 | 0 | 0 | 0.276882 | 0 | 0 | 0 |
| ICM7 | 0 | 0.037381 | 0.009997 | 0.024234 | 0 | 0.168805 | 0 | 0.039753 | 0.000282 | 0 | 0.057387 | 0.031799 | 0.063208 | 0.002731 | 0.02514 | 0.280272 | 0.002017 | 0 | 0.256996 | 0 | 0 | 0 |
| ICM8 | 0 | 0 | 0.00044 | 0.085177 | 0 | 0.140853 | 0 | 0.032477 | 0 | 0 | 0.030652 | 0 | 0.105956 | 0.08324 | 0.015438 | 0.304809 | 0 | 0 | 0.147777 | 0 | 0.053181 | 0 |
| NICM1 | 0 | 0.027066 | 0.0147 | 0.066105 | 0 | 0.117821 | 0 | 0 | 0 | 0 | 0.038519 | 0 | 0.031058 | 0 | 0.000965 | 0.415785 | 0 | 0 | 0.190076 | 0 | 0.028126 | 0.069778 |
| NICM2 | 0 | 0.003753 | 0.006923 | 0.006985 | 0 | 0.167234 | 0 | 0 | 0 | 0 | 0.070906 | 0 | 0.040122 | 0 | 0.002298 | 0.466404 | 0 | 0 | 0.214718 | 0 | 0.005122 | 0.015535 |
| NICM3 | 0.00882 | 0 | 0 | 0.040542 | 0 | 0.138596 | 0 | 0.001225 | 0.000163 | 0 | 0.087137 | 0 | 0.025692 | 0 | 0 | 0.413908 | 0 | 0.00362 | 0.249241 | 0 | 0 | 0.031056 |
| NICM4 | 0 | 0.021851 | 0 | 0.03185 | 0 | 0.219591 | 0 | 0.005874 | 0.014379 | 0 | 0.065433 | 0.014623 | 0.078139 | 0.047579 | 0.021056 | 0.220861 | 0.002899 | 0 | 0.215338 | 0 | 0.040527 | 0 |
| NICM5 | 0 | 0.046214 | 0.011751 | 0 | 0 | 0.068767 | 0 | 0.027651 | 0 | 0 | 0.082225 | 0 | 0.051674 | 0 | 0.016851 | 0.529105 | 0 | 0 | 0.144878 | 0 | 0 | 0.020884 |
| NICM6 | 0 | 0.049031 | 0.024028 | 0.133151 | 0 | 0.239235 | 0 | 0 | 0.027954 | 0 | 0 | 0.047447 | 0.072104 | 0.112089 | 0 | 0 | 0.000882 | 0 | 0.291562 | 0 | 0.002517 | 0 |
| NICM7 | 0 | 0.010749 | 0.020497 | 0.205292 | 0 | 0.120588 | 0 | 0.005771 | 0.008641 | 0 | 0.018441 | 0.092734 | 0.028846 | 0 | 0.038921 | 0.217084 | 0.003459 | 0 | 0.192162 | 0 | 0.031947 | 0.004867 |
| NICM8 | 0 | 0 | 0.007463 | 0.099315 | 0 | 0.140443 | 0 | 0.028643 | 0 | 0.007236 | 0.075011 | 0.053228 | 0.033598 | 0 | 0.103404 | 0.278831 | 0.004396 | 0 | 0.129735 | 0 | 0 | 0.038698 |

**Supplementary Table 2 Fibrosis scores and diagnosis of the selected patients**

| ID | Dataset | Subtype | Fibrosis_score | ICM/NICM | Etiology |
| --- | --- | --- | --- | --- | --- |
| DCM17 | GSE116250 | 1 | 0.458941659 | NICM | Dilated cardiomyopathy |
| DCM19 | GSE116250 | 1 | 0.492348143 | NICM | Dilated cardiomyopathy |
| DCM22 | GSE116250 | 1 | 0.476009275 | NICM | Dilated cardiomyopathy |
| DCM33 | GSE116250 | 1 | 0.467082108 | NICM | Dilated cardiomyopathy |
| DCM4 | GSE116250 | 1 | 0.460573061 | NICM | Dilated cardiomyopathy |
| ICM50 | GSE116250 | 1 | 0.470866631 | ICM | Ischemic cardiomyopathy |
| DCM72 | GSE116250 | 1 | 0.476058962 | NICM | Dilated cardiomyopathy |
| DCM78 | GSE116250 | 1 | 0.459686969 | NICM | Dilated cardiomyopathy |
| ICM1 | GSE46224 | 1 | 0.467305702 | ICM | Ischemic cardiomyopathy |
| ICM6 | GSE46224 | 1 | 0.480166453 | ICM | Ischemic cardiomyopathy |
| ICM.LVAD5 | GSE46224 | 1 | 0.488671277 | ICM | Ischemic cardiomyopathy |
| ICM.LVAD8 | GSE46224 | 1 | 0.473558031 | ICM | Ischemic cardiomyopathy |
| NICM4 | GSE46224 | 1 | 0.484240818 | NICM | Dilated cardiomyopathy |
| NICM.LVAD1 | GSE46224 | 1 | 0.490294398 | NICM | Dilated cardiomyopathy |
| SRR9856187 | GSE135055 | 1 | 0.467885388 | NICM | Dilated cardiomyopathy |
| SRR9856191 | GSE135055 | 1 | 0.469848039 | NICM | Dilated cardiomyopathy |
| SRR9856194 | GSE135055 | 1 | 0.463695085 | NICM | Dilated cardiomyopathy |
| SRR9856198 | GSE135055 | 1 | 0.473177094 | NICM | Dilated cardiomyopathy |
| SRR9856205 | GSE135055 | 1 | 0.460274937 | NICM | Myocarditis |
| SRR9856206 | GSE135055 | 1 | 0.479851766 | NICM | Myocarditis |
| DCM16 | GSE116250 | 1 | 0.455968697 | NICM | Dilated cardiomyopathy |
| DCM32 | GSE116250 | 1 | 0.401196638 | NICM | Dilated cardiomyopathy |
| DCM39 | GSE116250 | 1 | 0.427547514 | NICM | Dilated cardiomyopathy |
| ICM54 | GSE116250 | 1 | 0.458569003 | ICM | Ischemic cardiomyopathy |
| ICM63 | GSE116250 | 1 | 0.456763695 | ICM | Ischemic cardiomyopathy |
| DCM73 | GSE116250 | 1 | 0.442370916 | NICM | Dilated cardiomyopathy |
| ICM2 | GSE46224 | 1 | 0.454047451 | ICM | Ischemic cardiomyopathy |
| ICM3 | GSE46224 | 1 | 0.455645729 | ICM | Ischemic cardiomyopathy |
| ICM.LVAD1 | GSE46224 | 1 | 0.428897354 | ICM | Ischemic cardiomyopathy |
| ICM.LVAD7 | GSE46224 | 1 | 0.38001325 | ICM | Ischemic cardiomyopathy |
| NICM6 | GSE46224 | 1 | 0.453683077 | NICM | Dilated cardiomyopathy |
| SRR9856199 | GSE135055 | 1 | 0.454411826 | NICM | Dilated cardiomyopathy |
| SRR9856200 | GSE135055 | 1 | 0.431712973 | NICM | Dilated cardiomyopathy |
| DCM14 | GSE116250 | 2 | 0.475131465 | NICM | Dilated cardiomyopathy |
| DCM11 | GSE116250 | 2 | 0.467984763 | NICM | Dilated cardiomyopathy |
| DCM18 | GSE116250 | 2 | 0.461194153 | NICM | Dilated cardiomyopathy |
| DCM20 | GSE116250 | 2 | 0.474510372 | NICM | Dilated cardiomyopathy |
| DCM24 | GSE116250 | 2 | 0.470535382 | NICM | Dilated cardiomyopathy |
| DCM28 | GSE116250 | 2 | 0.462568838 | NICM | Dilated cardiomyopathy |
| DCM34 | GSE116250 | 2 | 0.470071633 | NICM | Dilated cardiomyopathy |
| DCM35 | GSE116250 | 2 | 0.460233531 | NICM | Dilated cardiomyopathy |
| DCM3 | GSE116250 | 2 | 0.478162395 | NICM | Dilated cardiomyopathy |
| DCM43 | GSE116250 | 2 | 0.473880999 | NICM | Dilated cardiomyopathy |
| DCM46 | GSE116250 | 2 | 0.466933046 | NICM | Dilated cardiomyopathy |
| ICM55 | GSE116250 | 2 | 0.465632893 | ICM | Ischemic cardiomyopathy |
| ICM56 | GSE116250 | 2 | 0.490948615 | ICM | Ischemic cardiomyopathy |
| ICM58 | GSE116250 | 2 | 0.485002691 | ICM | Ischemic cardiomyopathy |
| ICM64 | GSE116250 | 2 | 0.461301809 | ICM | Ischemic cardiomyopathy |
| DCM6 | GSE116250 | 2 | 0.471769285 | NICM | Dilated cardiomyopathy |
| DCM75 | GSE116250 | 2 | 0.468581011 | NICM | Dilated cardiomyopathy |
| ICM5 | GSE46224 | 2 | 0.474063186 | ICM | Ischemic cardiomyopathy |
| ICM7 | GSE46224 | 2 | 0.489698149 | ICM | Ischemic cardiomyopathy |
| ICM.LVAD2 | GSE46224 | 2 | 0.48189723 | ICM | Ischemic cardiomyopathy |
| NICM1 | GSE46224 | 2 | 0.477350834 | NICM | dilated cardiomyopathy/ hypertrophic cardiomyopathy |
| NICM5 | GSE46224 | 2 | 0.473367562 | NICM | Dilated cardiomyopathy |
| NICM.LVAD2 | GSE46224 | 2 | 0.481913792 | NICM | Dilated cardiomyopathy |
| NICM.LVAD5 | GSE46224 | 2 | 0.472001159 | NICM | Dilated cardiomyopathy |
| NICM.LVAD6 | GSE46224 | 2 | 0.468970229 | NICM | Dilated cardiomyopathy |
| SRR9856188 | GSE135055 | 2 | 0.462543994 | NICM | Dilated cardiomyopathy |
| SRR9856189 | GSE135055 | 2 | 0.469748665 | NICM | Dilated cardiomyopathy |
| SRR9856196 | GSE135055 | 2 | 0.464531489 | NICM | Dilated cardiomyopathy |
| SRR9856202 | GSE135055 | 2 | 0.465152582 | NICM | Dilated cardiomyopathy |
| SRR9856204 | GSE135055 | 2 | 0.468423668 | NICM | Dilated cardiomyopathy |
| DCM8 | GSE116250 | 2 | 0.41534098 | NICM | Dilated cardiomyopathy |
| DCM12 | GSE116250 | 2 | 0.43129063 | NICM | Dilated cardiomyopathy |
| DCM13 | GSE116250 | 2 | 0.386530578 | NICM | Dilated cardiomyopathy |
| DCM21 | GSE116250 | 2 | 0.37530123 | NICM | Dilated cardiomyopathy |
| DCM26 | GSE116250 | 2 | 0.45215105 | NICM | Dilated cardiomyopathy |
| DCM29 | GSE116250 | 2 | 0.428218293 | NICM | Dilated cardiomyopathy |
| DCM2 | GSE116250 | 2 | 0.444259037 | NICM | Dilated cardiomyopathy |
| DCM31 | GSE116250 | 2 | 0.402298041 | NICM | Dilated cardiomyopathy |
| DCM38 | GSE116250 | 2 | 0.426843609 | NICM | Dilated cardiomyopathy |
| DCM44 | GSE116250 | 2 | 0.422239245 | NICM | Dilated cardiomyopathy |
| ICM47 | GSE116250 | 2 | 0.434561716 | ICM | Ischemic cardiomyopathy |
| ICM49 | GSE116250 | 2 | 0.454792762 | ICM | Ischemic cardiomyopathy |
| ICM52 | GSE116250 | 2 | 0.387814169 | ICM | Ischemic cardiomyopathy |
| ICM53 | GSE116250 | 2 | 0.430744069 | ICM | Ischemic cardiomyopathy |
| ICM59 | GSE116250 | 2 | 0.407854747 | ICM | Ischemic cardiomyopathy |
| ICM61 | GSE116250 | 2 | 0.432715001 | ICM | Ischemic cardiomyopathy |
| DCM74 | GSE116250 | 2 | 0.402091011 | NICM | Dilated cardiomyopathy |
| DCM76 | GSE116250 | 2 | 0.419357377 | NICM | Dilated cardiomyopathy |
| DCM77 | GSE116250 | 2 | 0.445078879 | NICM | Dilated cardiomyopathy |
| ICM4 | GSE46224 | 2 | 0.44367107 | ICM | Ischemic cardiomyopathy |
| ICM8 | GSE46224 | 2 | 0.429344541 | ICM | Ischemic cardiomyopathy |
| ICM.LVAD3 | GSE46224 | 2 | 0.381992464 | ICM | Ischemic cardiomyopathy |
| ICM.LVAD4 | GSE46224 | 2 | 0.452879798 | ICM | Ischemic cardiomyopathy |
| ICM.LVAD6 | GSE46224 | 2 | 0.43923233 | ICM | Ischemic cardiomyopathy |
| NICM2 | GSE46224 | 2 | 0.448159497 | NICM | Dilated cardiomyopathy |
| NICM3 | GSE46224 | 2 | 0.440689827 | NICM | Dilated cardiomyopathy |
| NICM7 | GSE46224 | 2 | 0.442047948 | NICM | Dilated cardiomyopathy |
| NICM8 | GSE46224 | 2 | 0.432342346 | NICM | Dilated cardiomyopathy |
| NICM.LVAD3 | GSE46224 | 2 | 0.367458904 | NICM | Dilated cardiomyopathy |
| NICM.LVAD4 | GSE46224 | 2 | 0.458014161 | NICM | Dilated cardiomyopathy |
| NICM.LVAD7 | GSE46224 | 2 | 0.425725643 | NICM | Dilated cardiomyopathy |
| NICM.LVAD8 | GSE46224 | 2 | 0.390936193 | NICM | Dilated cardiomyopathy |
| SRR9856190 | GSE135055 | 2 | 0.442097636 | NICM | Dilated cardiomyopathy |
| SRR9856192 | GSE135055 | 2 | 0.447058093 | NICM | Dilated cardiomyopathy |
| SRR9856193 | GSE135055 | 2 | 0.433601093 | NICM | Dilated cardiomyopathy |
| SRR9856195 | GSE135055 | 2 | 0.402952259 | NICM | Dilated cardiomyopathy |
| SRR9856197 | GSE135055 | 2 | 0.422595338 | NICM | Dilated cardiomyopathy |
| SRR9856203 | GSE135055 | 2 | 0.4547762 | NICM | Dilated cardiomyopathy |

**Supplementary Table 3 Genes more up-regulated in subtype 1**

|  | Cluster1_log2FoldChange | Cluster1_pvalue | Cluster1_change (UP/NOT/DOWN) | Cluster2_log2FoldChange | Cluster2_pvalue | Cluster2_change (UP/NOT/DOWN) |
| --- | --- | --- | --- | --- | --- | --- |
| IGKC | 4.016653 | 1.97E-14 | UP | 0.572556 | 0.066318 | NOT |
| IGHG2 | 5.957875 | 1.46E-13 | UP | 0.782636 | 0.088613 | NOT |
| IGLC2 | 3.406395 | 2.65E-11 | UP | 0.370309 | 0.231934 | NOT |
| CD79A | 4.255659 | 3.24E-11 | UP | 0.99309 | 0.004219 | NOT |
| IGLL5 | 3.497251 | 5.72E-11 | UP | 0.853987 | 0.022979 | NOT |
| IGKV3-15 | 4.391153 | 6.92E-11 | UP | 0.941403 | 0.028899 | NOT |
| SLAMF7 | 2.885813 | 1.22E-10 | UP | 0.948719 | 7.91E-05 | NOT |
| IL7R | 2.287271 | 1.27E-10 | UP | 1.086113 | 2.59E-06 | UP |
| CD5 | 2.230092 | 1.78E-10 | UP | 1.080943 | 7.88E-07 | UP |
| LAMP3 | 3.430443 | 2.22E-10 | UP | 1.435192 | 0.000169 | UP |
| ITK | 2.048681 | 4.39E-10 | UP | 0.947833 | 6.58E-06 | NOT |
| IGLV2-14 | 3.576298 | 9.14E-10 | UP | 0.568339 | 0.130824 | NOT |
| CD48 | 1.677289 | 2.30E-09 | UP | 0.756961 | 1.61E-06 | NOT |
| IGLV2-11 | 3.778936 | 2.83E-09 | UP | 0.705223 | 0.077172 | NOT |
| IGLC3 | 2.975195 | 2.84E-09 | UP | 0.106141 | 0.746657 | NOT |
| BTLA | 2.51848 | 4.85E-09 | UP | 1.192889 | 0.000107 | UP |
| SDC1 | 2.361565 | 5.61E-09 | UP | 0.584435 | 0.007893 | NOT |
| FOXP3 | 2.146289 | 5.88E-09 | UP | 0.855222 | 4.49E-05 | NOT |
| IGKV3-11 | 3.871001 | 6.11E-09 | UP | 0.41406 | 0.293288 | NOT |
| IGKV2-30 | 4.339743 | 8.06E-09 | UP | 0.697344 | 0.171372 | NOT |
| TIGIT | 2.958715 | 9.11E-09 | UP | 1.084044 | 6.23E-05 | UP |
| RHOH | 2.238455 | 9.66E-09 | UP | 1.014727 | 6.97E-06 | UP |
| IGHG3 | 4.373362 | 1.11E-08 | UP | 0.231811 | 0.60671 | NOT |
| BCL2A1 | 2.258956 | 1.18E-08 | UP | 1.081862 | 4.59E-05 | UP |
| IGLV3-19 | 3.636725 | 1.39E-08 | UP | 0.578745 | 0.152477 | NOT |
| IGLV2-23 | 3.135808 | 1.50E-08 | UP | 0.182348 | 0.654748 | NOT |
| TNC | 2.022838 | 1.91E-08 | UP | 0.833911 | 0.005344 | NOT |
| MMP9 | 2.717267 | 3.08E-08 | UP | 0.799157 | 0.006997 | NOT |
| JCHAIN | 2.642531 | 3.40E-08 | UP | 0.069427 | 0.809612 | NOT |
| CD96 | 1.779164 | 4.78E-08 | UP | 0.792783 | 0.000288 | NOT |
| GZMK | 1.906788 | 5.09E-08 | UP | 0.921347 | 8.24E-05 | NOT |
| SIRPG | 2.710737 | 5.38E-08 | UP | 1.028287 | 0.008812 | UP |
| FCRL3 | 2.474623 | 5.40E-08 | UP | 1.149905 | 0.000121 | UP |
| JAK3 | 1.387036 | 6.74E-08 | UP | 0.651261 | 0.000319 | NOT |
| CILP2 | 2.007792 | 7.35E-08 | UP | 0.933268 | 0.005095 | NOT |
| CTLA4 | 1.662719 | 8.60E-08 | UP | 0.600031 | 0.003461 | NOT |
| ITGAX | 1.764099 | 9.60E-08 | UP | 0.850575 | 2.38E-05 | NOT |
| ZNF831 | 2.03316 | 1.01E-07 | UP | 1.011096 | 9.42E-06 | UP |
| TNFRSF9 | 2.572527 | 1.13E-07 | UP | 0.605918 | 0.059476 | NOT |
| CXCL8 | 1.663126 | 1.14E-07 | UP | 0.636972 | 0.06954 | NOT |
| FCRL5 | 3.936242 | 1.27E-07 | UP | 0.817973 | 0.055092 | NOT |
| TNFRSF18 | 2.98167 | 1.33E-07 | UP | 1.084691 | 0.006669 | UP |
| TNFAIP3 | 1.185039 | 1.39E-07 | UP | 0.472204 | 0.00013 | NOT |
| CXCL10 | 2.48183 | 1.54E-07 | UP | 1.067194 | 0.001508 | UP |
| IGKV4-1 | 2.65408 | 1.61E-07 | UP | 0.511672 | 0.129078 | NOT |
| PTPRN | 2.38448 | 2.14E-07 | UP | 0.535891 | 0.233539 | NOT |
| IGKV3-20 | 2.740757 | 2.44E-07 | UP | 0.545328 | 0.145753 | NOT |
| KLHDC7B | 2.389433 | 2.73E-07 | UP | 1.185887 | 0.0022 | UP |
| CARD11 | 1.498918 | 2.81E-07 | UP | 0.673955 | 4.39E-05 | NOT |
| LY9 | 2.219621 | 2.85E-07 | UP | 0.897319 | 0.000876 | NOT |
| MZB1 | 2.513221 | 2.86E-07 | UP | -0.25248 | 0.390826 | NOT |
| ANO9 | 1.420343 | 2.90E-07 | UP | 0.469041 | 0.006528 | NOT |
| IL2RB | 1.559828 | 3.57E-07 | UP | 0.764676 | 4.25E-05 | NOT |
| PLA2G7 | 2.26551 | 3.67E-07 | UP | 1.043832 | 0.00061 | UP |
| RASAL3 | 1.269515 | 3.94E-07 | UP | 0.575845 | 4.67E-05 | NOT |
| MYC | 1.306622 | 4.59E-07 | UP | 0.647491 | 0.000523 | NOT |
| CXCR3 | 2.101691 | 4.93E-07 | UP | 0.873711 | 0.001882 | NOT |
| GBP5 | 1.821323 | 5.28E-07 | UP | 0.672991 | 0.000498 | NOT |
| RTKN2 | 2.152226 | 5.52E-07 | UP | 0.464724 | 0.047115 | NOT |
| MS4A1 | 2.244208 | 6.19E-07 | UP | 0.422413 | 0.144749 | NOT |
| CSF3 | 3.157887 | 6.25E-07 | UP | 0.632747 | 0.240321 | NOT |
| CARMIL2 | 1.654571 | 6.81E-07 | UP | 0.606553 | 0.000653 | NOT |
| UBASH3A | 1.994287 | 7.64E-07 | UP | 0.65826 | 0.009027 | NOT |
| NELL2 | 1.892395 | 8.77E-07 | UP | 0.816369 | 0.000594 | NOT |
| CCL19 | 2.720784 | 9.87E-07 | UP | -0.36164 | 0.384613 | NOT |
| OLR1 | 1.776035 | 1.09E-06 | UP | 0.635472 | 0.023551 | NOT |
| P2RY10 | 2.274438 | 1.10E-06 | UP | 0.739272 | 0.002549 | NOT |
| PTGS2 | 1.542781 | 1.20E-06 | UP | 0.767073 | 0.001228 | NOT |
| SOCS3 | 1.82956 | 1.22E-06 | UP | 0.634489 | 0.039594 | NOT |
| BLK | 2.879013 | 1.25E-06 | UP | 1.354486 | 0.00084 | UP |
| TMC8 | 1.242933 | 1.32E-06 | UP | 0.553939 | 8.73E-06 | NOT |
| IL2RG | 1.467685 | 1.53E-06 | UP | 0.449073 | 0.004765 | NOT |
| TNFRSF13B | 3.451683 | 1.91E-06 | UP | 0.474847 | 0.392798 | NOT |
| MIR155HG | 1.591919 | 1.96E-06 | UP | 0.544028 | 0.011647 | NOT |
| CD8A | 1.518108 | 2.07E-06 | UP | 0.608925 | 0.002532 | NOT |
| CXCL11 | 2.62417 | 2.10E-06 | UP | 0.912518 | 0.006407 | NOT |
| IGLC5 | 2.953046 | 2.23E-06 | UP | 0.501337 | 0.278777 | NOT |
| KLHL6 | 1.036875 | 2.38E-06 | UP | 0.430636 | 0.000443 | NOT |
| IL6 | 1.708852 | 2.72E-06 | UP | 0.668969 | 0.064038 | NOT |
| C11orf96 | 1.25001 | 2.90E-06 | UP | 0.564304 | 0.003576 | NOT |
| OLFML2B | 1.063151 | 2.98E-06 | UP | 0.494829 | 0.01338 | NOT |
| CD27 | 1.405279 | 3.16E-06 | UP | 0.402262 | 0.00514 | NOT |
| IGKV1-16 | 3.619348 | 3.19E-06 | UP | 0.219567 | 0.71467 | NOT |
| TMEM156 | 2.356936 | 3.27E-06 | UP | 0.715244 | 0.027241 | NOT |
| CXCR2 | 1.528808 | 3.75E-06 | UP | 0.603934 | 0.023095 | NOT |
| CD22 | 1.881305 | 3.87E-06 | UP | 0.647109 | 0.004401 | NOT |
| GRAMD2A | 1.399187 | 3.91E-06 | UP | 0.64045 | 0.02159 | NOT |
| PTPN7 | 1.465901 | 3.98E-06 | UP | 0.575833 | 0.001243 | NOT |
| DCANP1 | 2.918387 | 4.14E-06 | UP | 1.041629 | 0.02297 | UP |
| ELAPOR1 | 1.652461 | 4.17E-06 | UP | 0.369857 | 0.104717 | NOT |
| SPIB | 2.30976 | 4.25E-06 | UP | 0.684843 | 0.026348 | NOT |
| FCHO1 | 1.157493 | 4.37E-06 | UP | 0.436337 | 0.004561 | NOT |
| IGKV1-27 | 3.997586 | 4.59E-06 | UP | 0.413297 | 0.466394 | NOT |
| CCR6 | 1.632078 | 5.43E-06 | UP | 0.398066 | 0.090567 | NOT |
| SKAP1 | 1.516515 | 5.57E-06 | UP | 0.660602 | 0.000841 | NOT |
| SH2D1A | 1.901689 | 5.88E-06 | UP | 0.651749 | 0.010145 | NOT |
| IL21R | 1.944608 | 6.80E-06 | UP | 0.875999 | 0.000299 | NOT |
| CD8B | 1.983542 | 7.26E-06 | UP | 0.927945 | 0.000658 | NOT |
| LINC00607 | 1.23024 | 7.98E-06 | UP | 0.59439 | 0.007137 | NOT |
| LINC00996 | 2.418432 | 8.06E-06 | UP | 1.150063 | 0.001113 | UP |
| CD7 | 1.596039 | 8.23E-06 | UP | 0.675708 | 0.001156 | NOT |
| CCR4 | 2.474688 | 9.57E-06 | UP | 1.054238 | 0.006872 | UP |
| VDR | 1.28724 | 1.01E-05 | UP | 0.336898 | 0.139767 | NOT |
| KCNA3 | 1.239943 | 1.15E-05 | UP | 0.602892 | 0.000858 | NOT |
| IGLV4-69 | 2.839121 | 1.39E-05 | UP | -0.32568 | 0.506214 | NOT |
| ANGPTL6 | 1.636281 | 1.44E-05 | UP | 0.673409 | 0.049371 | NOT |
| IGFBP2 | 1.060031 | 1.61E-05 | UP | 0.514841 | 0.006838 | NOT |
| IL4I1 | 2.026549 | 1.64E-05 | UP | 0.615627 | 0.041568 | NOT |
| LAX1 | 1.515484 | 1.69E-05 | UP | 0.361738 | 0.040411 | NOT |
| SFRP2 | 2.04354 | 1.74E-05 | UP | 0.778841 | 0.044397 | NOT |
| TRAT1 | 1.969611 | 1.80E-05 | UP | 0.763522 | 0.022217 | NOT |
| CD3G | 1.723997 | 1.84E-05 | UP | 0.857212 | 0.001294 | NOT |
| SIT1 | 1.86551 | 1.89E-05 | UP | 0.814535 | 0.003551 | NOT |
| POU2AF1 | 1.984584 | 1.91E-05 | UP | -0.32006 | 0.219219 | NOT |
| MIAT | 1.139982 | 1.99E-05 | UP | 0.517718 | 0.001031 | NOT |
| IGKV2D-28 | 3.637922 | 2.06E-05 | UP | 0.496162 | 0.405393 | NOT |
| ZNF80 | 2.365808 | 2.32E-05 | UP | 0.931436 | 0.013352 | NOT |
| WAKMAR2 | 1.149581 | 2.35E-05 | UP | 0.442476 | 0.03024 | NOT |
| TNFRSF17 | 2.137465 | 2.65E-05 | UP | 0.206267 | 0.629514 | NOT |
| GADD45B | 1.101649 | 2.78E-05 | UP | 0.399776 | 0.028118 | NOT |
| CYP27B1 | 1.210494 | 3.46E-05 | UP | 0.524738 | 0.011393 | NOT |
| LINC01215 | 2.881271 | 3.85E-05 | UP | 1.028084 | 0.024522 | UP |
| C15orf48 | 2.363332 | 4.05E-05 | UP | 0.454417 | 0.215661 | NOT |
| RND1 | 1.136678 | 4.27E-05 | UP | 0.532819 | 0.025331 | NOT |
| DENND1C | 1.040216 | 4.78E-05 | UP | 0.317128 | 0.03679 | NOT |
| ADAM12 | 1.325746 | 4.84E-05 | UP | 0.597418 | 0.032866 | NOT |
| IGKV2-24 | 2.608894 | 4.89E-05 | UP | 0.197484 | 0.686406 | NOT |
| TLR10 | 2.173691 | 5.21E-05 | UP | 0.846916 | 0.008598 | NOT |
| IGKV3D-20 | 3.44343 | 6.03E-05 | UP | 0.282193 | 0.607413 | NOT |
| ZBTB32 | 1.21779 | 6.33E-05 | UP | 0.384329 | 0.078352 | NOT |
| IGHG4 | 4.37538 | 6.62E-05 | UP | 0.81921 | 0.241621 | NOT |
| IRF4 | 1.539549 | 6.86E-05 | UP | -0.1754 | 0.343426 | NOT |
| GPR171 | 1.315832 | 7.48E-05 | UP | 0.320151 | 0.136893 | NOT |
| IGKV1-6 | 2.772666 | 7.60E-05 | UP | 0.479113 | 0.348908 | NOT |
| RGS16 | 1.03369 | 7.92E-05 | UP | 0.438057 | 0.021243 | NOT |
| PAX5 | 2.627382 | 8.00E-05 | UP | 1.046382 | 0.121338 | NOT |
| EPHA1 | 1.560668 | 8.13E-05 | UP | 0.566991 | 0.038264 | NOT |
| DNAJC5B | 2.649613 | 8.32E-05 | UP | 0.985917 | 0.055733 | NOT |
| IGKV1-39 | 2.8547 | 8.57E-05 | UP | 0.079683 | 0.88649 | NOT |
| STEAP1 | 1.074101 | 8.91E-05 | UP | 0.386891 | 0.084373 | NOT |
| PRSS46P | 1.490439 | 8.99E-05 | UP | 0.634724 | 0.069069 | NOT |
| IGKV2-28 | 3.116783 | 9.02E-05 | UP | 0.404883 | 0.483619 | NOT |
| IGKV1-33 | 3.382235 | 9.08E-05 | UP | 0.330966 | 0.565517 | NOT |
| MYO1G | 1.073791 | 0.000101 | UP | 0.463741 | 0.007175 | NOT |
| PRND | 2.664275 | 0.000107 | UP | 1.235693 | 0.01103 | UP |
| IGKV1D-39 | 2.818477 | 0.000109 | UP | 0.963881 | 0.10873 | NOT |
| CST7 | 1.073257 | 0.00012 | UP | 0.311644 | 0.122862 | NOT |
| DPEP1 | 2.573795 | 0.00012 | UP | 0.6477 | 0.257314 | NOT |
| IGLV1-47 | 2.237343 | 0.00012 | UP | 0.189303 | 0.701205 | NOT |
| CACNA1I | 1.954529 | 0.000122 | UP | 0.533863 | 0.134486 | NOT |
| FCRLA | 1.688615 | 0.000126 | UP | 0.574898 | 0.036154 | NOT |
| JSRP1 | 2.839485 | 0.000133 | UP | -0.10411 | 0.864965 | NOT |
| ASTL | 1.469124 | 0.000157 | UP | 0.680688 | 0.048631 | NOT |
| KCNN4 | 1.111391 | 0.000159 | UP | 0.191593 | 0.254677 | NOT |
| IGLV3-25 | 2.272276 | 0.000179 | UP | 0.592345 | 0.217825 | NOT |
| IGLV7-46 | 2.020587 | 0.000209 | UP | 0.289395 | 0.558455 | NOT |
| TRBC1 | 1.697737 | 0.000209 | UP | 0.558209 | 0.061855 | NOT |
| LIPG | 1.71762 | 0.000225 | UP | -0.01792 | 0.959948 | NOT |
| DTHD1 | 1.521897 | 0.000225 | UP | 0.687268 | 0.020457 | NOT |
| CLEC17A | 2.213419 | 0.000237 | UP | 0.956437 | 0.028707 | NOT |
| KMO | 1.438714 | 0.000242 | UP | 0.131393 | 0.577809 | NOT |
| CXCR5 | 1.657198 | 0.000244 | UP | 0.183875 | 0.55758 | NOT |
| PIEZO2 | 1.210606 | 0.000245 | UP | 0.570781 | 0.019181 | NOT |
| IGKV1-12 | 3.069424 | 0.000258 | UP | 0.61245 | 0.354464 | NOT |
| IGHV3-7 | 3.140644 | 0.000263 | UP | 0.279294 | 0.688916 | NOT |
| IGHG1 | 3.364332 | 0.00027 | UP | 0.871615 | 0.195098 | NOT |
| IGLV2-8 | 2.251549 | 0.000307 | UP | 0.76948 | 0.108234 | NOT |
| ETV7 | 1.489492 | 0.000312 | UP | 0.548284 | 0.085304 | NOT |
| ZBED2 | 2.444598 | 0.000323 | UP | 1.153398 | 0.019594 | UP |
| IGHA2 | 3.050138 | 0.00035 | UP | -0.13535 | 0.81173 | NOT |
| LGR5 | 1.240415 | 0.000387 | UP | 0.607735 | 0.03156 | NOT |
| NUGGC | 1.991387 | 0.000423 | UP | 0.648453 | 0.021066 | NOT |
| ANO3 | 1.484801 | 0.000449 | UP | 0.727914 | 0.038442 | NOT |
| FCRL1 | 1.710387 | 0.000456 | UP | 0.566675 | 0.16682 | NOT |
| KLRC2 | 1.611549 | 0.000466 | UP | 0.45617 | 0.362322 | NOT |
| AIM2 | 1.513378 | 0.000491 | UP | 0.32256 | 0.233068 | NOT |
| CNR2 | 2.529933 | 0.000515 | UP | 0.640212 | 0.283559 | NOT |
| IGLV10-54 | 3.074269 | 0.00061 | UP | 0.888635 | 0.245425 | NOT |
| PRSS50 | 1.183287 | 0.000615 | UP | -0.09103 | 0.759407 | NOT |
| CD19 | 1.779208 | 0.000623 | UP | 0.427657 | 0.255484 | NOT |
| FCRL2 | 2.949668 | 0.00072 | UP | 0.617379 | 0.141109 | NOT |
| MLNR | 1.188631 | 0.00075 | UP | 0.351796 | 0.265295 | NOT |
| CLNK | 2.574395 | 0.000751 | UP | 1.188204 | 0.046042 | UP |
| CD24 | 1.622705 | 0.000787 | UP | 0.589516 | 0.116899 | NOT |
| ANKRD36BP2 | 1.70299 | 0.000805 | UP | 0.028874 | 0.91182 | NOT |
| TNFSF15 | 1.621588 | 0.000867 | UP | -0.19123 | 0.598318 | NOT |
| VPREB3 | 1.033592 | 0.000953 | UP | 0.291144 | 0.213619 | NOT |
| CCR5 | 1.007643 | 0.001024 | UP | 0.140343 | 0.528584 | NOT |
| JAKMIP1 | 1.49414 | 0.001063 | UP | 0.664417 | 0.040683 | NOT |
| OSM | 1.343805 | 0.001168 | UP | 0.615212 | 0.087404 | NOT |
| LRG1 | 1.158431 | 0.001496 | UP | 0.358684 | 0.180415 | NOT |
| LGALS2 | 1.047306 | 0.001505 | UP | 0.193395 | 0.387551 | NOT |
| TEX41 | 1.481245 | 0.001564 | UP | 0.694535 | 0.130245 | NOT |
| FSCN3 | 1.18369 | 0.001657 | UP | 0.571873 | 0.156805 | NOT |
| CRTAM | 1.191618 | 0.001878 | UP | 0.449638 | 0.06459 | NOT |
| LINC01239 | 1.31501 | 0.001959 | UP | 0.506423 | 0.166653 | NOT |
| RAB3B | 1.735233 | 0.001973 | UP | 0.818766 | 0.243884 | NOT |
| GDF3 | 1.053573 | 0.001976 | UP | 0.187976 | 0.586481 | NOT |
| TRGC2 | 1.396351 | 0.002039 | UP | 0.633589 | 0.061955 | NOT |
| CAMK4 | 1.387371 | 0.002107 | UP | 0.515268 | 0.059353 | NOT |
| RASGRF1 | 1.379065 | 0.002241 | UP | 0.26398 | 0.444125 | NOT |
| FOSL1 | 1.205508 | 0.002254 | UP | 0.300023 | 0.386678 | NOT |
| ADTRP | 1.275724 | 0.002345 | UP | 0.219237 | 0.476184 | NOT |
| IGHA1 | 2.618816 | 0.002418 | UP | 0.375136 | 0.46936 | NOT |
| PNOC | 2.499672 | 0.002496 | UP | -0.016 | 0.977814 | NOT |
| FASLG | 1.541484 | 0.002516 | UP | 0.745531 | 0.022491 | NOT |
| CCL8 | 1.221602 | 0.002612 | UP | 0.529641 | 0.076596 | NOT |
| XCL2 | 1.532587 | 0.002728 | UP | 0.573388 | 0.088217 | NOT |
| AKT3 | 1.397455 | 0.002796 | UP | 0.050334 | 0.872363 | NOT |
| BATF | 1.02377 | 0.002971 | UP | -0.09115 | 0.666281 | NOT |
| LINC01013 | 1.714516 | 0.002989 | UP | 0.817828 | 0.116128 | NOT |
| IGLON5 | 1.512731 | 0.003244 | UP | 0.194068 | 0.633831 | NOT |
| BCL2L14 | 1.636594 | 0.003354 | UP | 0.522771 | 0.253722 | NOT |
| STAP1 | 1.419034 | 0.003473 | UP | -0.09653 | 0.756452 | NOT |
| HCAR2 | 1.549059 | 0.003607 | UP | 0.608044 | 0.181758 | NOT |
| TEX35 | 1.235916 | 0.003617 | UP | 0.409024 | 0.205592 | NOT |
| P4HA3 | 1.109098 | 0.003902 | UP | 0.311805 | 0.302603 | NOT |
| SFN | 1.23922 | 0.003907 | UP | 0.598714 | 0.12229 | NOT |
| SELE | 1.271389 | 0.0051 | UP | 0.231595 | 0.520605 | NOT |
| SLC12A3 | 2.02175 | 0.005112 | UP | 0.819844 | 0.175178 | NOT |
| CD1B | 1.514555 | 0.005189 | UP | 0.510285 | 0.307152 | NOT |
| CYP4F35P | 1.630663 | 0.005334 | UP | 0.425881 | 0.398854 | NOT |
| LINC01711 | 1.99939 | 0.005514 | UP | 0.540668 | 0.337072 | NOT |
| PDE6G | 1.406055 | 0.005774 | UP | 0.423117 | 0.227685 | NOT |
| IGKV1-5 | 1.048561 | 0.006735 | UP | 0.283448 | 0.456686 | NOT |
| TNFSF18 | 1.330135 | 0.006871 | UP | -0.02235 | 0.970722 | NOT |
| IGHV2-70 | 2.117178 | 0.00748 | UP | 0.196023 | 0.773754 | NOT |
| GLDC | 1.204535 | 0.007805 | UP | 0.264709 | 0.511833 | NOT |
| DKK4 | 1.038992 | 0.007942 | UP | -0.09792 | 0.801749 | NOT |
| IGKV1-9 | 1.778667 | 0.008105 | UP | -0.12196 | 0.809159 | NOT |
| WNT10A | 1.792218 | 0.008164 | UP | -0.78728 | 0.11 | NOT |
| IGLV3-10 | 1.689318 | 0.008453 | UP | -0.2133 | 0.703741 | NOT |
| AMPD1 | 1.29623 | 0.008499 | UP | 0.183814 | 0.636678 | NOT |
| IGLV3-27 | 2.480247 | 0.009248 | UP | 0.390506 | 0.670293 | NOT |
| ADAMDEC1 | 1.687512 | 0.00945 | UP | -0.57639 | 0.305388 | NOT |
| IGKV2D-29 | 2.536047 | 0.009955 | UP | 0.451189 | 0.558416 | NOT |
| ALOX15B | 1.522921 | 0.010554 | UP | 0.36215 | 0.405752 | NOT |
| IGKV3D-11 | 1.89236 | 0.010564 | UP | -0.10677 | 0.885622 | NOT |
| LOC643802 | 1.263485 | 0.01117 | UP | 0.357861 | 0.434247 | NOT |
| GAD1 | 1.122303 | 0.011235 | UP | 0.553056 | 0.191216 | NOT |
| IDO2 | 1.72079 | 0.011306 | UP | 0.572922 | 0.227431 | NOT |
| TMEM88B | 1.362038 | 0.011351 | UP | 0.391871 | 0.554642 | NOT |
| IGKV2D-30 | 2.193224 | 0.011908 | UP | -0.28101 | 0.742342 | NOT |
| DCSTAMP | 1.971069 | 0.012109 | UP | 0.759505 | 0.226296 | NOT |
| MIR21 | 1.14631 | 0.012338 | UP | 0.434619 | 0.248226 | NOT |
| SLC27A2 | 1.289638 | 0.012452 | UP | 0.520351 | 0.188766 | NOT |
| HES2 | 1.80863 | 0.01263 | UP | 0.518366 | 0.480977 | NOT |
| IGLV8-61 | 1.630415 | 0.013283 | UP | 0.410623 | 0.405578 | NOT |
| PRKCG | 1.19378 | 0.013353 | UP | 0.183901 | 0.727671 | NOT |
| LRAT | 1.372593 | 0.013512 | UP | 0.642084 | 0.167104 | NOT |
| TRAV19 | 1.543637 | 0.014146 | UP | 0.545331 | 0.288244 | NOT |
| IGKV3D-15 | 2.097838 | 0.01421 | UP | 0.714071 | 0.314142 | NOT |
| IGLV9-49 | 2.311737 | 0.014402 | UP | 0.711718 | 0.373478 | NOT |
| IGHV3-23 | 1.709002 | 0.014593 | UP | -0.44707 | 0.398784 | NOT |
| FNDC7 | 1.273205 | 0.014623 | UP | 0.568727 | 0.232646 | NOT |
| BANK1 | 1.014431 | 0.014758 | UP | -0.31024 | 0.227785 | NOT |
| DRD1 | 1.426935 | 0.015162 | UP | 0.524176 | 0.31537 | NOT |
| ADM2 | 1.339871 | 0.015465 | UP | 0.141532 | 0.793889 | NOT |
| LHFPL4 | 1.088864 | 0.015652 | UP | 0.507434 | 0.221725 | NOT |
| HOXD9 | 1.033973 | 0.016143 | UP | -0.04658 | 0.889782 | NOT |
| ISL2 | 1.395282 | 0.017866 | UP | 0.406864 | 0.449751 | NOT |
| TC2N | 1.516174 | 0.018978 | UP | 0.475384 | 0.370443 | NOT |
| EGR4 | 2.251393 | 0.022419 | UP | 0.944276 | 0.28054 | NOT |
| LINC01700 | 1.335415 | 0.024579 | UP | 0.642717 | 0.227366 | NOT |
| MAFA-AS1 | 1.216879 | 0.025284 | UP | 0.568643 | 0.200673 | NOT |
| DEFB132 | 1.419455 | 0.027691 | UP | 0.249626 | 0.62583 | NOT |
| CLLU1 | 1.640719 | 0.028695 | UP | 0.732016 | 0.259497 | NOT |
| TRAV39 | 1.471829 | 0.029037 | UP | 0.615355 | 0.402657 | NOT |
| PLK5 | 1.487998 | 0.029742 | UP | 0.448552 | 0.454233 | NOT |
| LARGE2 | 1.250128 | 0.030157 | UP | 0.195013 | 0.716401 | NOT |
| CDH1 | 1.67225 | 0.031109 | UP | -0.43065 | 0.501636 | NOT |
| ADAMTS18 | 1.388228 | 0.031988 | UP | 0.365072 | 0.54421 | NOT |
| IGHV3-21 | 3.361734 | 0.032135 | UP | 0.352374 | 0.663838 | NOT |
| TBC1D21 | 1.019666 | 0.033064 | UP | 0.344002 | 0.390386 | NOT |
| ADIPOQ | 1.136413 | 0.037625 | UP | 0.272014 | 0.556089 | NOT |
| TAC1 | 1.121297 | 0.03785 | UP | -0.39311 | 0.429971 | NOT |
| ISM2 | 1.501825 | 0.038265 | UP | 0.627452 | 0.314607 | NOT |
| EN2 | 1.016851 | 0.038313 | UP | 0.233921 | 0.605977 | NOT |
| HCAR3 | 1.068714 | 0.038367 | UP | 0.327825 | 0.494528 | NOT |
| DMP1 | 1.481452 | 0.0397 | UP | 0.181049 | 0.803384 | NOT |
| RASSF6 | 1.591134 | 0.040618 | UP | -0.2958 | 0.677932 | NOT |
| BOLA2B | 1.305051 | 0.041021 | UP | -0.61163 | 0.13888 | NOT |
| VSIG1 | 1.158368 | 0.044145 | UP | 0.411674 | 0.307618 | NOT |
| SLC6A7 | 1.197328 | 0.045087 | UP | 0.430203 | 0.354827 | NOT |
| CWC25 | 1.340035 | 0.045565 | UP | 0.115732 | 0.836963 | NOT |

**Supplementary Table 4 Common up regulated in high-fibrotic samples**

|  | Subtype 1 high-fibrotic vs low-fibrotic | | | | | | | Subtype 2 high-fibrotic vs low-fibrotic | | | | | | |
| --- | --- | --- | --- | --- | --- | --- | --- | --- | --- | --- | --- | --- | --- | --- |
| Gene Symbols | baseMean | log2FoldChange | lfcSE | stat | pvalue | padj | change (UP/NOT/DOWN) | baseMean | log2FoldChange | lfcSE | stat | pvalue | padj | change (UP/NOT/DOWN) |
| POSTN | 6851.747498 | 2.082103 | 0.306522 | 6.792679 | 1.10E-11 | 1.71E-07 | UP | 4155.302 | 2.001999 | 0.193172 | 10.36383 | 3.62E-25 | 2.32E-21 | UP |
| SCRG1 | 59.82521011 | 1.486913 | 0.22178 | 6.704447 | 2.02E-11 | 1.71E-07 | UP | 47.27076 | 1.311488 | 0.169174 | 7.752303 | 9.02E-15 | 3.43E-12 | UP |
| CDH11 | 520.973334 | 1.296928 | 0.197041 | 6.582017 | 4.64E-11 | 2.09E-07 | UP | 377.597 | 1.001144 | 0.096609 | 10.36284 | 3.66E-25 | 2.32E-21 | UP |
| FAP | 235.3358324 | 1.650647 | 0.262015 | 6.299822 | 2.98E-10 | 1.01E-06 | UP | 177.2661 | 1.216367 | 0.162799 | 7.471571 | 7.92E-14 | 2.21E-11 | UP |
| SFRP4 | 1066.039043 | 2.089389 | 0.348604 | 5.993592 | 2.05E-09 | 4.97E-06 | UP | 623.082 | 1.856393 | 0.214854 | 8.640249 | 5.61E-18 | 4.63E-15 | UP |
| MGP | 10147.40422 | 1.109765 | 0.186036 | 5.965324 | 2.44E-09 | 5.18E-06 | UP | 8204.012 | 1.074793 | 0.134542 | 7.988561 | 1.37E-15 | 6.82E-13 | UP |
| AEBP1 | 6119.850494 | 1.560288 | 0.269396 | 5.791806 | 6.96E-09 | 1.07E-05 | UP | 4093.22 | 1.643895 | 0.168126 | 9.777771 | 1.40E-22 | 3.80E-19 | UP |
| TMEM119 | 229.5799287 | 1.747358 | 0.308668 | 5.660963 | 1.51E-08 | 1.96E-05 | UP | 136.3029 | 1.783342 | 0.167632 | 10.63842 | 1.97E-26 | 3.75E-22 | UP |
| ITGBL1 | 692.44269 | 1.904573 | 0.340916 | 5.586644 | 2.32E-08 | 2.52E-05 | UP | 422.5344 | 1.469911 | 0.155333 | 9.462944 | 2.99E-21 | 5.17E-18 | UP |
| SPON1 | 901.4864861 | 1.284112 | 0.230056 | 5.581745 | 2.38E-08 | 2.52E-05 | UP | 663.5538 | 1.100975 | 0.134352 | 8.194703 | 2.51E-16 | 1.64E-13 | UP |
| F2RL2 | 121.3674702 | 1.478516 | 0.266076 | 5.556744 | 2.75E-08 | 2.74E-05 | UP | 88.6777 | 1.551015 | 0.232161 | 6.680785 | 2.38E-11 | 3.79E-09 | UP |
| COL10A1 | 42.85989887 | 2.970512 | 0.537692 | 5.524556 | 3.30E-08 | 2.95E-05 | UP | 16.24794 | 1.563898 | 0.249881 | 6.258558 | 3.89E-10 | 4.50E-08 | UP |
| CTHRC1 | 123.1863858 | 1.639166 | 0.30416 | 5.38915 | 7.08E-08 | 5.22E-05 | UP | 76.83062 | 1.187382 | 0.206884 | 5.739351 | 9.50E-09 | 6.33E-07 | UP |
| SERPINE2 | 2115.79684 | 1.414669 | 0.26487 | 5.340984 | 9.24E-08 | 6.20E-05 | UP | 1436.508 | 1.168969 | 0.145006 | 8.061508 | 7.54E-16 | 4.42E-13 | UP |
| SULF1 | 1070.182669 | 1.144233 | 0.214 | 5.346878 | 8.95E-08 | 6.20E-05 | UP | 890.7027 | 1.012511 | 0.129867 | 7.796505 | 6.36E-15 | 2.55E-12 | UP |
| COL3A1 | 14838.91517 | 1.192984 | 0.228427 | 5.222617 | 1.76E-07 | 8.80E-05 | UP | 10539.39 | 1.32755 | 0.137288 | 9.66983 | 4.05E-22 | 9.61E-19 | UP |
| CRISPLD1 | 138.3659736 | 1.517702 | 0.303967 | 4.992977 | 5.95E-07 | 0.000203 | UP | 133.9917 | 1.860781 | 0.185635 | 10.02384 | 1.20E-23 | 3.79E-20 | UP |
| PODNL1 | 64.85686953 | 1.42892 | 0.287233 | 4.97477 | 6.53E-07 | 0.000213 | UP | 47.96895 | 1.116978 | 0.196932 | 5.671884 | 1.41E-08 | 8.76E-07 | UP |
| ASPN | 3066.159726 | 1.369037 | 0.276153 | 4.957533 | 7.14E-07 | 0.000224 | UP | 2245.059 | 1.172647 | 0.133023 | 8.815341 | 1.19E-18 | 1.19E-15 | UP |
| BGN | 8515.124996 | 1.066682 | 0.216734 | 4.921611 | 8.58E-07 | 0.000239 | UP | 6384.978 | 1.129114 | 0.121336 | 9.305676 | 1.33E-20 | 1.84E-17 | UP |
| COL11A1 | 11.05603162 | 3.143055 | 0.637149 | 4.932998 | 8.10E-07 | 0.000239 | UP | 6.141625 | 2.052172 | 0.371116 | 5.529728 | 3.21E-08 | 1.73E-06 | UP |
| COL1A1 | 10055.44883 | 1.325715 | 0.270433 | 4.902192 | 9.48E-07 | 0.000251 | UP | 6201.809 | 1.474492 | 0.144027 | 10.23761 | 1.35E-24 | 5.16E-21 | UP |
| ESM1 | 28.38308528 | 1.795018 | 0.366403 | 4.89902 | 9.63E-07 | 0.000251 | UP | 22.9901 | 1.277088 | 0.312147 | 4.091305 | 4.29E-05 | 0.000681 | UP |
| FMOD | 3735.940556 | 1.817168 | 0.37081 | 4.900542 | 9.56E-07 | 0.000251 | UP | 2474.514 | 1.779465 | 0.200957 | 8.854942 | 8.37E-19 | 8.83E-16 | UP |
| ADAMTS2 | 392.5441413 | 1.131341 | 0.231621 | 4.884448 | 1.04E-06 | 0.000256 | UP | 306.1243 | 1.138869 | 0.139857 | 8.143087 | 3.85E-16 | 2.36E-13 | UP |
| MFAP2 | 65.46367902 | 1.159451 | 0.243328 | 4.764963 | 1.89E-06 | 0.000376 | UP | 48.92545 | 1.096888 | 0.140381 | 7.813666 | 5.55E-15 | 2.29E-12 | UP |
| VCAN | 2511.72855 | 1.145739 | 0.241963 | 4.735175 | 2.19E-06 | 0.0004 | UP | 2092.602 | 1.223649 | 0.141516 | 8.6467 | 5.30E-18 | 4.57E-15 | UP |
| CFH | 3580.480577 | 1.138865 | 0.242041 | 4.705259 | 2.54E-06 | 0.000444 | UP | 2839.279 | 1.139348 | 0.149692 | 7.611295 | 2.71E-14 | 8.45E-12 | UP |
| COL1A2 | 15268.07349 | 1.046667 | 0.224677 | 4.658542 | 3.18E-06 | 0.000516 | UP | 10908.43 | 1.197785 | 0.11701 | 10.23665 | 1.36E-24 | 5.16E-21 | UP |
| ZNF365 | 40.74043096 | 1.534008 | 0.330116 | 4.646882 | 3.37E-06 | 0.000529 | UP | 35.15203 | 1.521965 | 0.16312 | 9.330322 | 1.06E-20 | 1.67E-17 | UP |
| CRACD | 63.25530362 | 1.243625 | 0.268148 | 4.637834 | 3.52E-06 | 0.000538 | UP | 48.88873 | 1.191346 | 0.169482 | 7.029321 | 2.08E-12 | 4.28E-10 | UP |
| COMP | 764.5999508 | 2.86883 | 0.619464 | 4.631148 | 3.64E-06 | 0.000546 | UP | 360.0664 | 2.565924 | 0.364045 | 7.04836 | 1.81E-12 | 3.78E-10 | UP |
| GDF6 | 87.0956336 | 1.566792 | 0.34016 | 4.606039 | 4.10E-06 | 0.000571 | UP | 51.08192 | 1.179281 | 0.207329 | 5.68798 | 1.29E-08 | 8.11E-07 | UP |
| NGFR | 447.5318458 | 1.284142 | 0.278732 | 4.607078 | 4.08E-06 | 0.000571 | UP | 335.1372 | 1.211002 | 0.15152 | 7.992361 | 1.32E-15 | 6.82E-13 | UP |
| LOX | 427.2604302 | 1.067163 | 0.232542 | 4.589124 | 4.45E-06 | 0.000604 | UP | 327.8415 | 1.08496 | 0.144461 | 7.510398 | 5.89E-14 | 1.70E-11 | UP |
| CPXM2 | 652.1006821 | 1.317159 | 0.290764 | 4.52999 | 5.90E-06 | 0.000739 | UP | 468.7872 | 1.453979 | 0.192269 | 7.562205 | 3.96E-14 | 1.18E-11 | UP |
| RIMS4 | 12.22138975 | 2.031184 | 0.449923 | 4.514518 | 6.35E-06 | 0.000769 | UP | 8.192627 | 1.805258 | 0.268355 | 6.727115 | 1.73E-11 | 2.91E-09 | UP |
| GPC6 | 249.3228569 | 1.410459 | 0.31315 | 4.5041 | 6.67E-06 | 0.000797 | UP | 162.6978 | 1.212369 | 0.206914 | 5.859295 | 4.65E-09 | 3.63E-07 | UP |
| MXRA5 | 2228.074583 | 1.157507 | 0.257086 | 4.502417 | 6.72E-06 | 0.000797 | UP | 1497.574 | 1.029659 | 0.16628 | 6.192304 | 5.93E-10 | 6.42E-08 | UP |
| KCNN1 | 3.354041337 | 3.072213 | 0.684795 | 4.486326 | 7.25E-06 | 0.000842 | UP | 2.012524 | 1.522496 | 0.335945 | 4.531981 | 5.84E-06 | 0.000133 | UP |
| SEZ6L | 155.1501779 | 1.603193 | 0.35987 | 4.454923 | 8.39E-06 | 0.000937 | UP | 106.9241 | 1.390903 | 0.226016 | 6.153994 | 7.56E-10 | 7.92E-08 | UP |
| PENK | 295.006174 | 1.805442 | 0.405811 | 4.448972 | 8.63E-06 | 0.000957 | UP | 196.5693 | 1.651972 | 0.26588 | 6.213235 | 5.19E-10 | 5.76E-08 | UP |
| EFEMP1 | 2361.231099 | 1.183602 | 0.266225 | 4.445864 | 8.75E-06 | 0.000957 | UP | 1770.403 | 1.049925 | 0.162771 | 6.450303 | 1.12E-10 | 1.55E-08 | UP |
| LINC02593 | 28.22141833 | 1.462503 | 0.329398 | 4.439932 | 9.00E-06 | 0.000966 | UP | 21.34836 | 1.690222 | 0.239741 | 7.050199 | 1.79E-12 | 3.77E-10 | UP |
| PIEZO2 | 17.35359406 | 1.754552 | 0.397475 | 4.414247 | 1.01E-05 | 0.001049 | UP | 9.748095 | 1.138806 | 0.213432 | 5.335698 | 9.52E-08 | 4.40E-06 | UP |
| SYT12 | 19.58111839 | 1.734129 | 0.393302 | 4.409152 | 1.04E-05 | 0.001065 | UP | 13.54281 | 1.40989 | 0.252713 | 5.579019 | 2.42E-08 | 1.37E-06 | UP |
| FRZB | 743.4093745 | 1.239166 | 0.283757 | 4.366997 | 1.26E-05 | 0.001194 | UP | 571.2542 | 1.146234 | 0.169953 | 6.744407 | 1.54E-11 | 2.65E-09 | UP |
| RUNX1 | 405.3047775 | 1.495482 | 0.344931 | 4.335603 | 1.45E-05 | 0.001298 | UP | 245.2072 | 1.078576 | 0.19279 | 5.594566 | 2.21E-08 | 1.30E-06 | UP |
| MDFI | 36.43026244 | 1.346272 | 0.313188 | 4.298606 | 1.72E-05 | 0.001445 | UP | 27.8728 | 1.634139 | 0.294826 | 5.542722 | 2.98E-08 | 1.63E-06 | UP |
| SCG2 | 62.83613708 | 2.255287 | 0.525735 | 4.28978 | 1.79E-05 | 0.001456 | UP | 28.64892 | 1.775561 | 0.317013 | 5.600908 | 2.13E-08 | 1.25E-06 | UP |
| PRND | 5.214802307 | 4.025396 | 0.940616 | 4.279533 | 1.87E-05 | 0.001492 | UP | 1.778401 | 1.560643 | 0.472307 | 3.304295 | 0.000952 | 0.008347 | UP |
| CILP2 | 20.09805689 | 2.101085 | 0.491468 | 4.275119 | 1.91E-05 | 0.001502 | UP | 11.12095 | 1.606841 | 0.333325 | 4.820639 | 1.43E-06 | 4.14E-05 | UP |
| COL14A1 | 2757.236326 | 1.158642 | 0.272492 | 4.252014 | 2.12E-05 | 0.001597 | UP | 2020.302 | 1.356168 | 0.142502 | 9.516865 | 1.78E-21 | 3.76E-18 | UP |
| CCDC80 | 8533.22483 | 1.069636 | 0.254171 | 4.20833 | 2.57E-05 | 0.001881 | UP | 6991.805 | 1.167644 | 0.148067 | 7.885907 | 3.12E-15 | 1.35E-12 | UP |
| OGN | 1786.066189 | 1.137924 | 0.270399 | 4.208313 | 2.57E-05 | 0.001881 | UP | 1562.676 | 1.08204 | 0.155954 | 6.93818 | 3.97E-12 | 8.02E-10 | UP |
| KCNMA1 | 170.0775226 | 1.045792 | 0.24882 | 4.203005 | 2.63E-05 | 0.00191 | UP | 134.2647 | 1.174385 | 0.15491 | 7.581064 | 3.43E-14 | 1.03E-11 | UP |
| ANGPTL5 | 72.23249188 | 1.233859 | 0.294146 | 4.194722 | 2.73E-05 | 0.001943 | UP | 69.80346 | 1.032035 | 0.213911 | 4.824605 | 1.40E-06 | 4.08E-05 | UP |
| STEAP1 | 32.17664629 | 1.594102 | 0.381221 | 4.181574 | 2.89E-05 | 0.00199 | UP | 19.22283 | 1.128607 | 0.22861 | 4.936825 | 7.94E-07 | 2.56E-05 | UP |
| LTBP2 | 8593.645312 | 1.122845 | 0.272359 | 4.122671 | 3.75E-05 | 0.002344 | UP | 6923.372 | 1.072827 | 0.155709 | 6.889954 | 5.58E-12 | 1.09E-09 | UP |
| CST2 | 11.60881463 | 2.580173 | 0.626052 | 4.121337 | 3.77E-05 | 0.002348 | UP | 5.072804 | 1.361285 | 0.392852 | 3.465132 | 0.00053 | 0.005254 | UP |
| P4HA3 | 12.44574834 | 1.602617 | 0.389543 | 4.114098 | 3.89E-05 | 0.002383 | UP | 7.199524 | 1.293904 | 0.233233 | 5.547687 | 2.89E-08 | 1.59E-06 | UP |
| DUXAP8 | 11.72365539 | 1.197202 | 0.294073 | 4.071112 | 4.68E-05 | 0.00271 | UP | 8.122274 | 1.031429 | 0.279092 | 3.695659 | 0.000219 | 0.002569 | UP |
| PRUNE2 | 152.6712314 | 1.352099 | 0.336292 | 4.020607 | 5.80E-05 | 0.003068 | UP | 114.2204 | 1.218082 | 0.247386 | 4.92382 | 8.49E-07 | 2.67E-05 | UP |
| THY1 | 281.6951133 | 1.124506 | 0.28385 | 3.961621 | 7.44E-05 | 0.003736 | UP | 192.2649 | 1.075951 | 0.242658 | 4.434027 | 9.25E-06 | 0.000193 | UP |
| PRRX2 | 26.49622493 | 1.338922 | 0.338036 | 3.960884 | 7.47E-05 | 0.003737 | UP | 19.19931 | 1.509998 | 0.232527 | 6.493861 | 8.37E-11 | 1.19E-08 | UP |
| TMEM130 | 16.54629656 | 2.075312 | 0.527542 | 3.93393 | 8.36E-05 | 0.004062 | UP | 15.58581 | 2.837549 | 0.388113 | 7.311138 | 2.65E-13 | 6.29E-11 | UP |
| CTSK | 707.9122647 | 1.109071 | 0.283097 | 3.917636 | 8.94E-05 | 0.004226 | UP | 474.0791 | 1.007961 | 0.128763 | 7.828035 | 4.96E-15 | 2.09E-12 | UP |
| NR2F1-AS1 | 22.67068868 | 1.318618 | 0.341696 | 3.859038 | 0.000114 | 0.004877 | UP | 15.89312 | 1.182252 | 0.213091 | 5.548114 | 2.89E-08 | 1.59E-06 | UP |
| PCDH10 | 15.73311511 | 1.655089 | 0.431162 | 3.838674 | 0.000124 | 0.005182 | UP | 10.98135 | 2.110487 | 0.353052 | 5.977836 | 2.26E-09 | 1.95E-07 | UP |
| ADAM12 | 50.24920008 | 1.393107 | 0.364289 | 3.824182 | 0.000131 | 0.005351 | UP | 33.2619 | 1.439398 | 0.238695 | 6.030272 | 1.64E-09 | 1.52E-07 | UP |
| IL11 | 3.379090506 | 2.101349 | 0.550689 | 3.815854 | 0.000136 | 0.005469 | UP | 2.398341 | 1.169548 | 0.353259 | 3.310736 | 0.000931 | 0.008198 | UP |
| CHST6 | 91.28746057 | 1.377634 | 0.365054 | 3.773784 | 0.000161 | 0.006048 | UP | 56.20873 | 1.612973 | 0.201208 | 8.016462 | 1.09E-15 | 5.90E-13 | UP |
| ID4 | 419.1949354 | 1.205047 | 0.319401 | 3.772832 | 0.000161 | 0.006058 | UP | 297.3833 | 1.5809 | 0.202886 | 7.79207 | 6.59E-15 | 2.55E-12 | UP |
| SCUBE2 | 292.5436956 | 1.101741 | 0.294955 | 3.735285 | 0.000188 | 0.006602 | UP | 226.8421 | 1.268906 | 0.166236 | 7.63314 | 2.29E-14 | 7.46E-12 | UP |
| THBS4 | 15018.2249 | 1.099881 | 0.295052 | 3.727753 | 0.000193 | 0.006716 | UP | 12004.51 | 1.23916 | 0.204446 | 6.061074 | 1.35E-09 | 1.28E-07 | UP |
| CYP1B1 | 1874.188106 | 1.110377 | 0.301495 | 3.68291 | 0.000231 | 0.007655 | UP | 1546.633 | 1.036032 | 0.175822 | 5.892494 | 3.80E-09 | 3.09E-07 | UP |
| P2RX1 | 95.54027475 | 1.530684 | 0.417736 | 3.664235 | 0.000248 | 0.008001 | UP | 47.0461 | 1.192337 | 0.240003 | 4.968001 | 6.76E-07 | 2.27E-05 | UP |
| TMEM30B | 106.9652663 | 1.167513 | 0.32117 | 3.635189 | 0.000278 | 0.008568 | UP | 83.11532 | 1.446632 | 0.189218 | 7.645332 | 2.08E-14 | 7.19E-12 | UP |
| DHRS9 | 161.0781797 | 1.399241 | 0.386217 | 3.622938 | 0.000291 | 0.00882 | UP | 90.45333 | 1.083944 | 0.24393 | 4.443665 | 8.84E-06 | 0.000187 | UP |
| CHRDL1 | 410.2439724 | 1.315746 | 0.365659 | 3.598284 | 0.00032 | 0.009274 | UP | 359.9085 | 1.652561 | 0.215812 | 7.657417 | 1.90E-14 | 6.80E-12 | UP |
| UCMA | 10.85894524 | 2.86604 | 0.80021 | 3.581609 | 0.000341 | 0.009753 | UP | 4.4064 | 1.637342 | 0.508823 | 3.217903 | 0.001291 | 0.010477 | UP |
| SHISA2 | 27.28630439 | 1.302516 | 0.365154 | 3.567031 | 0.000361 | 0.010124 | UP | 21.37378 | 1.482263 | 0.230498 | 6.430696 | 1.27E-10 | 1.69E-08 | UP |
| COL22A1 | 116.9180116 | 1.824386 | 0.514025 | 3.549218 | 0.000386 | 0.010624 | UP | 73.78213 | 2.088191 | 0.34598 | 6.035579 | 1.58E-09 | 1.48E-07 | UP |
| GDNF | 14.7967242 | 1.0688 | 0.301997 | 3.539103 | 0.000401 | 0.010829 | UP | 14.75739 | 1.115102 | 0.22004 | 5.067718 | 4.03E-07 | 1.46E-05 | UP |
| LINC00968 | 26.65881157 | 1.18099 | 0.333653 | 3.539575 | 0.000401 | 0.010829 | UP | 24.36622 | 1.033175 | 0.172824 | 5.978179 | 2.26E-09 | 1.95E-07 | UP |
| AIRE | 3.060403145 | 1.961503 | 0.555007 | 3.534195 | 0.000409 | 0.010945 | UP | 1.946778 | 1.106622 | 0.375204 | 2.949388 | 0.003184 | 0.020859 | UP |
| GDF10 | 64.46692466 | 1.141338 | 0.326475 | 3.495944 | 0.000472 | 0.012033 | UP | 59.86902 | 1.46878 | 0.230736 | 6.365629 | 1.94E-10 | 2.48E-08 | UP |
| GALNT5 | 12.88153313 | 1.199492 | 0.344261 | 3.484256 | 0.000494 | 0.012367 | UP | 11.30065 | 1.166038 | 0.192348 | 6.06212 | 1.34E-09 | 1.28E-07 | UP |
| THBS4-AS1 | 41.60427127 | 1.110985 | 0.319182 | 3.48073 | 0.0005 | 0.012457 | UP | 30.81211 | 1.088537 | 0.195895 | 5.556734 | 2.75E-08 | 1.53E-06 | UP |
| CP | 179.5257714 | 1.209167 | 0.34834 | 3.471225 | 0.000518 | 0.012738 | UP | 159.427 | 1.284369 | 0.285169 | 4.503884 | 6.67E-06 | 0.000149 | UP |
| SYNPR | 9.468977606 | 2.307721 | 0.665809 | 3.466039 | 0.000528 | 0.012875 | UP | 6.348829 | 1.138663 | 0.403656 | 2.820872 | 0.004789 | 0.028647 | UP |
| NPY1R | 60.41512336 | 1.085942 | 0.313572 | 3.463139 | 0.000534 | 0.012921 | UP | 48.26471 | 1.034118 | 0.20112 | 5.141796 | 2.72E-07 | 1.05E-05 | UP |
| SAMD11 | 59.38734349 | 1.346502 | 0.39196 | 3.435305 | 0.000592 | 0.013774 | UP | 43.16931 | 1.742318 | 0.216992 | 8.02941 | 9.79E-16 | 5.47E-13 | UP |
| CCN2 | 6676.765435 | 1.061065 | 0.31375 | 3.381877 | 0.00072 | 0.015499 | UP | 5362.033 | 1.253393 | 0.213912 | 5.859386 | 4.65E-09 | 3.63E-07 | UP |
| RASEF | 10.79975263 | 1.401143 | 0.414889 | 3.377155 | 0.000732 | 0.015583 | UP | 8.231349 | 1.022386 | 0.249888 | 4.091378 | 4.29E-05 | 0.000681 | UP |
| CRABP2 | 46.40419453 | 1.036897 | 0.308416 | 3.362004 | 0.000774 | 0.016188 | UP | 32.98127 | 1.537876 | 0.200928 | 7.653871 | 1.95E-14 | 6.86E-12 | UP |
| MYL1 | 22.86560632 | 1.533555 | 0.457313 | 3.353402 | 0.000798 | 0.016462 | UP | 20.85583 | 1.337091 | 0.343984 | 3.887068 | 0.000101 | 0.001395 | UP |
| PTPRN | 6.241078097 | 1.768182 | 0.528268 | 3.347129 | 0.000817 | 0.01669 | UP | 4.586174 | 1.275505 | 0.350538 | 3.638705 | 0.000274 | 0.003085 | UP |
| NRG1 | 61.42034367 | 1.818389 | 0.545042 | 3.336238 | 0.000849 | 0.017182 | UP | 40.63256 | 1.189735 | 0.305551 | 3.893738 | 9.87E-05 | 0.001362 | UP |
| MSTN | 25.3752459 | 1.644007 | 0.497949 | 3.301556 | 0.000962 | 0.018473 | UP | 19.87293 | 1.40571 | 0.250938 | 5.601817 | 2.12E-08 | 1.25E-06 | UP |
| LINC01426 | 15.23928247 | 1.074056 | 0.326989 | 3.284683 | 0.001021 | 0.019034 | UP | 10.83383 | 1.074573 | 0.221046 | 4.861321 | 1.17E-06 | 3.48E-05 | UP |
| PRSS35 | 24.42033871 | 1.243333 | 0.381653 | 3.257761 | 0.001123 | 0.020181 | UP | 17.32223 | 1.516078 | 0.295032 | 5.138693 | 2.77E-07 | 1.06E-05 | UP |
| GRIA3 | 133.1654205 | 1.061204 | 0.327477 | 3.24055 | 0.001193 | 0.021083 | UP | 116.5124 | 1.089721 | 0.25322 | 4.303447 | 1.68E-05 | 0.000316 | UP |
| IGHG1 | 542.6453231 | 4.838033 | 1.494424 | 3.23739 | 0.001206 | 0.021105 | UP | 23.86667 | 1.881293 | 0.822509 | 2.287263 | 0.022181 | 0.08899 | UP |
| KCNQ5 | 4.110580304 | 1.867667 | 0.579949 | 3.220399 | 0.00128 | 0.021739 | UP | 2.96121 | 1.391032 | 0.356841 | 3.898182 | 9.69E-05 | 0.001343 | UP |
| FBLN7 | 43.7614692 | 1.072806 | 0.333227 | 3.219445 | 0.001284 | 0.021748 | UP | 26.67164 | 1.092763 | 0.165292 | 6.611117 | 3.81E-11 | 5.89E-09 | UP |
| TNMD | 118.6680804 | 2.091787 | 0.650451 | 3.215904 | 0.0013 | 0.021899 | UP | 75.23308 | 1.956039 | 0.4149 | 4.714478 | 2.42E-06 | 6.46E-05 | UP |
| STAC2 | 10.62718414 | 1.186939 | 0.371786 | 3.192535 | 0.00141 | 0.023181 | UP | 7.636296 | 1.018782 | 0.275317 | 3.700399 | 0.000215 | 0.002527 | UP |
| SFRP2 | 98.3679555 | 2.136647 | 0.675361 | 3.163711 | 0.001558 | 0.024665 | UP | 38.06804 | 1.341592 | 0.414478 | 3.236825 | 0.001209 | 0.009951 | UP |
| HTR2A | 13.53383458 | 1.378571 | 0.43619 | 3.160479 | 0.001575 | 0.024857 | UP | 9.326497 | 1.697565 | 0.286261 | 5.930137 | 3.03E-09 | 2.54E-07 | UP |
| NR2F1 | 40.0363445 | 1.088414 | 0.350011 | 3.109652 | 0.001873 | 0.027728 | UP | 25.25218 | 1.011958 | 0.226567 | 4.466483 | 7.95E-06 | 0.000171 | UP |
| LYPD1 | 9.556564519 | 1.581971 | 0.510307 | 3.100042 | 0.001935 | 0.028339 | UP | 7.173638 | 1.995048 | 0.359279 | 5.552918 | 2.81E-08 | 1.56E-06 | UP |
| ANO3 | 9.093075986 | 1.609531 | 0.519839 | 3.096207 | 0.00196 | 0.028551 | UP | 4.644299 | 1.664987 | 0.325887 | 5.109097 | 3.24E-07 | 1.21E-05 | UP |
| HTR2B | 68.7580188 | 1.177974 | 0.383747 | 3.069661 | 0.002143 | 0.030488 | UP | 54.26667 | 1.388137 | 0.263081 | 5.276458 | 1.32E-07 | 5.73E-06 | UP |
| PPP2R2B | 42.71802331 | 1.220497 | 0.398332 | 3.064018 | 0.002184 | 0.0309 | UP | 38.53667 | 1.484756 | 0.275082 | 5.397512 | 6.76E-08 | 3.31E-06 | UP |
| XG | 63.75796274 | 1.329592 | 0.436377 | 3.046886 | 0.002312 | 0.03184 | UP | 56.14399 | 1.422718 | 0.267697 | 5.314658 | 1.07E-07 | 4.86E-06 | UP |
| KCNK17 | 24.10357428 | 1.315489 | 0.43332 | 3.035839 | 0.002399 | 0.032698 | UP | 15.21007 | 1.367458 | 0.34747 | 3.935464 | 8.30E-05 | 0.001175 | UP |
| C3orf80 | 8.291738651 | 1.287142 | 0.425476 | 3.025182 | 0.002485 | 0.033456 | UP | 7.041547 | 1.358508 | 0.228793 | 5.937708 | 2.89E-09 | 2.45E-07 | UP |
| KCNS1 | 5.213535191 | 1.979243 | 0.655066 | 3.02144 | 0.002516 | 0.033656 | UP | 3.153055 | 1.607693 | 0.321916 | 4.994136 | 5.91E-07 | 2.01E-05 | UP |
| IGHM | 5.042120988 | 3.183968 | 1.063718 | 2.993245 | 0.00276 | 0.035765 | UP | 1.481042 | 1.994667 | 0.893597 | 2.232178 | 0.025603 | 0.098576 | UP |
| SLITRK4 | 160.8757397 | 1.0277 | 0.345776 | 2.972156 | 0.002957 | 0.037551 | UP | 166.5555 | 1.045277 | 0.313306 | 3.336283 | 0.000849 | 0.007643 | UP |
| LINC01235 | 6.1084881 | 1.670196 | 0.566053 | 2.950598 | 0.003172 | 0.039476 | UP | 2.319567 | 1.223332 | 0.420994 | 2.905817 | 0.003663 | 0.023227 | UP |
| C8orf34 | 14.11512279 | 1.047871 | 0.357648 | 2.929893 | 0.003391 | 0.041148 | UP | 12.67139 | 1.133937 | 0.229064 | 4.9503 | 7.41E-07 | 2.43E-05 | UP |
| LINC00673 | 7.003862304 | 1.104796 | 0.378948 | 2.91543 | 0.003552 | 0.042436 | UP | 6.125564 | 1.279768 | 0.242485 | 5.277711 | 1.31E-07 | 5.72E-06 | UP |
| RGS4 | 243.7057258 | 1.331092 | 0.458583 | 2.902623 | 0.003701 | 0.043747 | UP | 149.6296 | 1.151142 | 0.283894 | 4.054833 | 5.02E-05 | 0.000772 | UP |
| GLIS1 | 31.54746855 | 1.044316 | 0.365089 | 2.860446 | 0.00423 | 0.047467 | UP | 24.08015 | 1.311769 | 0.230035 | 5.702485 | 1.18E-08 | 7.60E-07 | UP |
| BDKRB2 | 44.3786588 | 1.170052 | 0.411208 | 2.845403 | 0.004436 | 0.049086 | UP | 28.24239 | 1.311998 | 0.261205 | 5.02286 | 5.09E-07 | 1.78E-05 | UP |
| TEX41 | 2.563454207 | 1.671896 | 0.600504 | 2.784154 | 0.005367 | 0.055857 | UP | 1.35374 | 1.248886 | 0.457427 | 2.730241 | 0.006329 | 0.035349 | UP |
| CA12 | 14.41121026 | 1.519588 | 0.549533 | 2.765232 | 0.005688 | 0.058098 | UP | 5.132034 | 1.238102 | 0.373639 | 3.31363 | 0.000921 | 0.008132 | UP |
| RSPO3 | 29.48436218 | 1.062747 | 0.384523 | 2.76381 | 0.005713 | 0.058247 | UP | 20.66896 | 1.137645 | 0.219886 | 5.173796 | 2.29E-07 | 9.05E-06 | UP |
| TMEM200A | 21.17819436 | 1.231486 | 0.450438 | 2.733974 | 0.006257 | 0.061398 | UP | 12.85394 | 1.256876 | 0.296914 | 4.233134 | 2.30E-05 | 0.000411 | UP |
| CALCA | 5.727981122 | 1.657195 | 0.612993 | 2.70345 | 0.006862 | 0.064534 | UP | 3.576877 | 1.974525 | 0.494253 | 3.99497 | 6.47E-05 | 0.000955 | UP |
| TAF7L | 3.158086824 | 1.123414 | 0.421624 | 2.664491 | 0.007711 | 0.069284 | UP | 2.814052 | 1.009038 | 0.335317 | 3.00921 | 0.002619 | 0.017978 | UP |
| CHRNE | 30.3016649 | 1.069529 | 0.402487 | 2.657301 | 0.007877 | 0.070174 | UP | 27.6596 | 1.002619 | 0.257583 | 3.892413 | 9.93E-05 | 0.001367 | UP |
| LAMP5 | 13.65642945 | 1.256745 | 0.473493 | 2.654203 | 0.00795 | 0.070533 | UP | 9.385548 | 1.633962 | 0.259567 | 6.294953 | 3.07E-10 | 3.69E-08 | UP |
| LINC02544 | 4.679149526 | 1.810835 | 0.686048 | 2.639515 | 0.008302 | 0.072058 | UP | 2.444573 | 1.87249 | 0.517161 | 3.620713 | 0.000294 | 0.003262 | UP |
| MYF6 | 6.748401889 | 1.696095 | 0.642901 | 2.638192 | 0.008335 | 0.072119 | UP | 4.221804 | 1.108911 | 0.482829 | 2.296694 | 0.021636 | 0.087567 | UP |
| CCNO | 4.705503353 | 1.499539 | 0.577563 | 2.596321 | 0.009423 | 0.077865 | UP | 3.942272 | 1.145344 | 0.354982 | 3.226487 | 0.001253 | 0.010246 | UP |
| DPEP1 | 4.479778339 | 1.946711 | 0.751921 | 2.588985 | 0.009626 | 0.078711 | UP | 1.083752 | 1.740495 | 0.504738 | 3.448316 | 0.000564 | 0.005493 | UP |
| DRD1 | 2.694935826 | 1.589139 | 0.615249 | 2.582918 | 0.009797 | 0.079258 | UP | 1.2976 | 1.152886 | 0.457551 | 2.519689 | 0.011746 | 0.055885 | UP |
| ATP1B4 | 139.9686041 | 1.23638 | 0.48238 | 2.563082 | 0.010375 | 0.082017 | UP | 89.40002 | 1.691103 | 0.309072 | 5.471556 | 4.46E-08 | 2.33E-06 | UP |
| LEF1-AS1 | 2.609217295 | 1.935801 | 0.756711 | 2.558178 | 0.010522 | 0.082614 | UP | 1.453594 | 1.58981 | 0.416791 | 3.814404 | 0.000137 | 0.00178 | UP |
| LINC01711 | 3.246421545 | 1.884025 | 0.76247 | 2.470952 | 0.013475 | 0.09717 | UP | 1.458769 | 1.488572 | 0.450854 | 3.30167 | 0.000961 | 0.008409 | UP |
| LOC149684 | 2.75538332 | 1.478297 | 0.600166 | 2.463147 | 0.013772 | 0.098461 | UP | 1.924439 | 1.190221 | 0.430302 | 2.76601 | 0.005675 | 0.032577 | UP |
| RSPO2 | 4.748995769 | 1.255779 | 0.51089 | 2.458019 | 0.013971 | 0.099334 | UP | 4.256727 | 1.615573 | 0.392069 | 4.120639 | 3.78E-05 | 0.000611 | UP |
| LUZP2 | 6.905188717 | 1.994233 | 0.817118 | 2.44057 | 0.014664 | 0.1022 | UP | 4.112566 | 1.801195 | 0.352169 | 5.114577 | 3.14E-07 | 1.18E-05 | UP |
| PTGFR | 83.75074705 | 1.035803 | 0.428015 | 2.420013 | 0.01552 | 0.106263 | UP | 59.55499 | 1.260265 | 0.229217 | 5.498123 | 3.84E-08 | 2.01E-06 | UP |
| LINC01563 | 3.2168511 | 1.22123 | 0.50498 | 2.418375 | 0.01559 | 0.106604 | UP | 2.880964 | 1.083951 | 0.325661 | 3.328467 | 0.000873 | 0.007791 | UP |
| CA3 | 752.8725387 | 1.202809 | 0.503751 | 2.387706 | 0.016954 | 0.112222 | UP | 665.5529 | 1.617879 | 0.369485 | 4.378738 | 1.19E-05 | 0.000238 | UP |
| TMEM114 | 4.688671352 | 2.36699 | 0.999462 | 2.368264 | 0.017872 | 0.115856 | UP | 2.799246 | 2.494906 | 0.560858 | 4.44837 | 8.65E-06 | 0.000184 | UP |
| ACVR1C | 20.4001797 | 1.259392 | 0.535361 | 2.352418 | 0.018652 | 0.118869 | UP | 18.03632 | 1.010228 | 0.362381 | 2.787755 | 0.005307 | 0.031052 | UP |
| HEPACAM | 4.507238648 | 1.585431 | 0.685302 | 2.31348 | 0.020696 | 0.126511 | UP | 4.924589 | 1.17485 | 0.445746 | 2.635695 | 0.008397 | 0.043626 | UP |
| PLPP4 | 4.457105154 | 1.491146 | 0.645418 | 2.310359 | 0.020868 | 0.127052 | UP | 2.560384 | 1.669038 | 0.381094 | 4.379601 | 1.19E-05 | 0.000238 | UP |
| IGFN1 | 11.27947629 | 1.215235 | 0.539093 | 2.254224 | 0.024182 | 0.139162 | UP | 6.507317 | 1.042111 | 0.293213 | 3.554113 | 0.000379 | 0.003985 | UP |
| SIX1 | 6.044723484 | 1.525571 | 0.690597 | 2.209059 | 0.027171 | 0.148406 | UP | 3.814158 | 1.718057 | 0.451544 | 3.804849 | 0.000142 | 0.001836 | UP |
| AFF2 | 14.38863782 | 1.053219 | 0.480752 | 2.190775 | 0.028468 | 0.152643 | UP | 15.55654 | 1.15925 | 0.246495 | 4.702936 | 2.56E-06 | 6.79E-05 | UP |
| PENK-AS1 | 3.73867989 | 1.090125 | 0.504431 | 2.161099 | 0.030688 | 0.159405 | UP | 2.879986 | 1.028655 | 0.305217 | 3.370237 | 0.000751 | 0.006928 | UP |
| SNAP25 | 9.524156259 | 1.218067 | 0.565539 | 2.153818 | 0.031254 | 0.161018 | UP | 7.017338 | 1.465878 | 0.360118 | 4.070549 | 4.69E-05 | 0.000734 | UP |
| CCL19 | 78.65248804 | 1.904078 | 0.888305 | 2.143496 | 0.032073 | 0.163449 | UP | 8.186652 | 1.204326 | 0.460632 | 2.614507 | 0.008936 | 0.045714 | UP |
| PCK1 | 38.88049653 | 1.958573 | 0.931281 | 2.103095 | 0.035457 | 0.173904 | UP | 51.96441 | 1.494594 | 0.590763 | 2.529939 | 0.011408 | 0.054684 | UP |
| PLIN1 | 290.6625591 | 1.479704 | 0.713424 | 2.074088 | 0.038071 | 0.181464 | UP | 312.5702 | 1.184477 | 0.451684 | 2.622356 | 0.008732 | 0.04494 | UP |
| SLC7A4 | 4.523384419 | 1.069654 | 0.519658 | 2.058379 | 0.039554 | 0.185419 | UP | 4.330126 | 1.263786 | 0.368295 | 3.431452 | 0.0006 | 0.005795 | UP |
| TACR1 | 11.35606084 | 1.036022 | 0.509731 | 2.032487 | 0.042104 | 0.19212 | UP | 7.659655 | 1.344205 | 0.296358 | 4.535746 | 5.74E-06 | 0.000131 | UP |
| KRT17 | 3.792765785 | 1.360993 | 0.670207 | 2.030704 | 0.042285 | 0.192685 | UP | 2.197961 | 1.473386 | 0.469375 | 3.139037 | 0.001695 | 0.012872 | UP |
| MMP1 | 3.491341271 | 1.085029 | 0.544738 | 1.991838 | 0.046389 | 0.20383 | UP | 2.226728 | 1.442046 | 0.396692 | 3.635174 | 0.000278 | 0.003122 | UP |

**Supplementary Table 5 EMT-related Transcription factor correlations with fibrosis**

|  | Coefficients | P-value | Significant |
| --- | --- | --- | --- |
| PAWR | 0.249822 | 0.010929046 | YES |
| HDAC4 | -0.03065 | 0.758557185 | NO |
| TAF6L | 0.194181 | 0.049365673 | YES |
| KLF8 | 0.345207 | 0.000355423 | YES |
| IRF2BP2 | 0.22273 | 0.023736585 | YES |
| MAML2 | 0.450538 | 1.79E-06 | YES |
| SMAD4 | -0.01528 | 0.878216636 | NO |
| CTBP1 | 0.171134 | 0.083916658 | NO |
| TRANK1 | 0.406932 | 1.99E-05 | YES |
| CNBP | -0.16578 | 0.094213638 | NO |
| FOSB | 0.148405 | 0.134636797 | NO |
| FOSL1 | 0.163535 | 0.098825363 | NO |
| FOSL2 | 0.139375 | 0.160289615 | NO |
| GTF2I | 0.066956 | 0.501587672 | NO |
| HOXB6 | 0.094061 | 0.344626473 | NO |
| IRF1 | 0.096764 | 0.330877846 | NO |
| IRF9 | 0.237844 | 0.015554079 | YES |
| JUNB | 0.260923 | 0.007766881 | YES |
| ZBTB7A | 0.296533 | 0.002354298 | YES |
| SMAD7 | 0.430178 | 5.76E-06 | YES |
| MEF2B | -0.04856 | 0.626192515 | NO |
| MNT | 0.385002 | 5.93E-05 | YES |
| NCOA2 | -0.03891 | 0.696370528 | NO |
| NFKBIB | 0.352455 | 0.000260942 | YES |
| PBX3 | 0.2441 | 0.012961876 | YES |
| PML | 0.309457 | 0.001468823 | YES |
| POU2F1 | 0.250493 | 0.010709978 | YES |
| SUB1 | 0.055692 | 0.576333644 | NO |
| RXRB | 0.135205 | 0.17329384 | NO |
| SOX4 | 0.21352 | 0.030342792 | YES |
| SP1 | 0.357888 | 0.000205982 | YES |
| ZEB1 | 0.442144 | 2.93E-06 | YES |
| TCF3 | 0.490448 | 1.45E-07 | YES |
| NFAT5 | 0.359643 | 0.000190662 | YES |
| RYBP | 0.31298 | 0.001286801 | YES |
| TRIP4 | 0.306975 | 0.00161076 | YES |
| ELF4 | 0.353969 | 0.000244404 | YES |
| SMARCE1 | 0.384656 | 6.03E-05 | YES |
| NRIP2 | 0.327712 | 0.000727127 | YES |
| REXO1 | 0.084062 | 0.398546246 | NO |
| TAF5L | 0.139419 | 0.160154738 | NO |
| SNAI1 | 0.286758 | 0.003317284 | YES |
| ZEB2 | 0.473008 | 4.53E-07 | YES |
| FOXC1 | 0.487119 | 1.81E-07 | YES |
| FOXF2 | 0.329458 | 0.000678269 | YES |

**Supplementary Table 6 ImmunCard30 genes’ Functional relationships with heart failure and pathological correlations of protein levels in liquid biopsy samples.**

| **Gene** | **Relevance to heart failure** | **Effects on heart failure progression (Promote/ Inhibit/ Not confirmed)** | **Pathological correlations of protein levels in blood** |
| --- | --- | --- | --- |
| TNFRSF17 | TNFRSF17 has been identified as a key driver of coronary artery disease (1). | **Promote** | TNFRSF17 is important for B-cell development, with especially high expression level in plasma cells (2, 3). Its expression level in whole blood positively correlates with coronary artery disease (1). |
| MEP1B | Participate in fibrillar collagen deposition, thereby promoting fibrosis and cardiac remodeling (4, 5). Apart from that, it also promotes inflammation (6) | **Promote** | Blood concentration of Meprin A (protein encoded by MEP1B) is correlated with fibrosis (7). Expression of MEP1B in blood cells is important for regulating pro-inflammatory pathways and maintaining homeostasis of immune cells (8, 9). |
| PLXNC1 | Plexin C1 (protein expressed by PLXCN1) is important in promoting acute inflammation in organs including heart, due to its ability to promote transmigration of leukocytes (10, 11). | **Promote** | Expression level of PLXCN1 in circulating blood cells is a key feature of abnormal inflammation in cardiovascular diseases (11). |
| TNFRSF9 | TNFRSF9 promotes activity of inflammatory cytokines in CD4^+^ T cells, thereby promote inflammation and plaque instability (12). However, its effects on prognosis of heart failure in general is beneficial, with hazard ratio < 1 (P-value 0.0488) according to a clinical study (13). | **Not confirmed** | TNFRSF9 in blood plasma was identified as a positive prognostic biomarker to heart failure (13). Clinical study on patients at risk for heart failure identified an improvement in plasma level of TNFRSF9 after treatment of mineralocorticoid receptor antagonist (14). |
| DUOX2 | DUOX2 is up-regulated in myocardium of both ischemic and dilated cardiomyopathy, which leads to oxidative stress that result in death of cardiomyocytes and cardiac remodeling (15). | **Promote** | Expression level of DUOX2 in blood of patients with cardiovascular disease positively correlated with inflammatory genes, especially those related to the regulatory system of NF-κB (16). |
| MADCAM1 | MADCAM1 was positively correlated with cardiac hypertrophy on mice models of cardiovascular remodeling (17). | **Promote** | MADCAM1 promotes adhesion of immune cells to vascular endothelium. Concentration of MADCAM1 in blood serum and urine is a good parameter for monitoring chronic inflammation (18). |
| SLITRK3 | This gene was found to be up-regulated in vascular smooth muscle of patients with myocardial infarction (19). | **Not confirmed** | Blood and tissue levels of SLITRK3 seems to be correlated with oxidative stress and immune activation (20). |
| CST6 | No proven effects on heart failure, but expresses in immune cells and performs as an inhibitor of cytotoxic T cells’ killing effect (21). | **Not confirmed** | Circulating level of CST6 was up-regulated in heart failure patients during treatment with empagliflozin (21). |
| IGFBP3 | IGFBP3 can promote the functions of IGF, thereby improve cardiac haemodynamics and support myocardial energy metabolism (22, 23). | **Inhibit** | Circulating level of IGFBP3 in blood is a biomarker for positive prognosis of heart failure (higher concentration of IGFBP3 corresponds to better prognosis) (22, 23). |
| ST6GAL2 | As a sialyltransferase, Promoting fibrosis of organs (24). | **Promote** | Concentration in peripheral blood mononuclear cells positively correlated with levels of sialic acids (25), while plasma level of sialic acid is a biomarker of systemic inflammation in cardiovascular diseases and a biomarker of severity for heart failure (26). |
| F11 | F11 suppresses inflammation and fibrosis, meanwhile prevents diastolic dysfunction, thereby protecting against heart failure (27). However, the up-regulation of F11 is also related to thrombosis and ischemic cardiomyopathy due to its coagulation effect (28). | **Promote/ Inhibit** | Circulating F11 is a biomarker for thromboembolic complications of heart failure (28). Meanwhile, its high level in blood correlates with active anti-fibrotic and anti-inflammatory effects (27). |
| MYBPH | MYBPH is important for contractility of myocardium (29). It’s also important for differentiating myocytes into Purkinje fibers that regulate cardiac rhythm (30). However, it is also responsively up-regulated by vascular and muscular injury (31, 32). | **Inhibit** | Expression level of MYBPH quantified based on whole blood demonstrated that people with higher arterial stiffness were having reduced expression level of MYBPH in blood (33). |
| CANT1 | CANT1 promotes signaling of NF-κB and inflammatory cytokines (34). It’s also playing a crucial role in ossification (35). | **Promote** | Expression level of CANT1 in peripheral blood from patients of ischemic stroke was elevated compared with healthy donors, and positively correlated with inflammatory immune cells in the blood (36). |
| IGF2BP1 | IGF2BP1 in cardiomyocyte was up-regulated by inflammation (37). It also promote the generation of inflammatory factors (38). | **Promote** | IGF2BP1 in blood cells is positively correlated with generation of inflammatory cytokines and activation of NF-κB in immune cells (38). |
| IL13 | IL13 is a pro-inflammatory cytokine that promotes fibrosis in heart failure and was typically expressed in CD4^+^ T cells (39). | **Promote** | Concentration of IL13 in blood serum correlates well with onset of heart failure in patients with coronary artery disease (40). |
| LRP8 | LRP8 prevents myocardial ischemia and suppresses the expression of pro-inflammatory cytokines IL-1β and IL-6 (41). | **Inhibit** | LRP8 on circulating blood cells like platelet and monocytes can reduce responses to inflammatory stimulus and against thrombosis (42). |
| DKK4 | DKK4 is related to cardiac fibrosis, heart failure, and myocardial infarction, due to its role in WNT signaling (43, 44). | **Promote** | Patients with spontaneous myocardial infarction have increased circulating DKK4 levels (45). |
| AMY1B | Blood level of amylase was elevated with myocardial infarction and advanced heart failure (46, 47). | **Not confirmed** | Plasma level of amylase positively correlated with pro-inflammatory cytokines in heart failure patients, especially those end-stage patients (47). |
| LIMD1 | LIMD1 suppresses Hippo signaling and thereby promote activation of fibroblast to a fibrotic phenotype (48). | **Promote** | Expression of LIMD1 in peripheral blood mononuclear cells negatively correlated with infectious responses (49). |
| VSIG4 | VSIG4 negatively regulate T cell proliferation, IL-2 generation, and wound healing processes (50). | **Inhibit** | VSIG4 in blood serum of heart failure patients was significantly higher than in healthy people, with good performance in classification (51). |
| ADAMTS5 | ADAMTS5 has cardioprotective effects during development of heart failure and cardiac remodeling, by controlling fibrosis and inflammation (52). | **Inhibit** | ADAMTS5 level in plasma is reduced in patients of coronary artery disease, meanwhile negatively correlated with inflammation biomarkers like CRP (53). |
| KLK12 | KLK12 promotes blood vessel formation (54), and was up-regulated in diabetes-associated cardiomyopathy (55). | **Not confirmed** | Plasma level of KLK12 is a biomarker of bad prognosis for heart failure (Hazard ratio 1.34, P-value 0.0256) (13). |
| FAH | Reduced expression of FAH is correlated with myocardial hypertrophy and heart failure induced by overexpression of myotrophin (56). | **Not confirmed** | No current recordings related to its liquid biopsy. However, it was recorded to be detectable in blood plasma according to the Human Protein Atlas(57). |
| H4C4 | It was expressed significantly higher in blood of heart failure with preserved ejection fraction (HFpEF), compared with those with heart failure with reduced ejection fraction (HFrEF) (58), meanwhile being down-regulated in blood of coronary heart disease compared with healthy people (59). | **Not confirmed** | Expression level of H4C4 in blood cells was different between HFpEF and HFrEF (58). Meanwhile, its expression in blood cells was also down-regulated in coronary heart disease (59). |
| RPS17 | Ribosomal proteins were up-regulated in chronic heart failure and early left ventricular dysfunction (60). | **Not confirmed** | No current recordings related to its liquid biopsy. However, it was recorded to be detectable in blood plasma according to the Human Protein Atlas(57). |
| VEGFD | VEGFD promotes blood vessel formation, meanwhile is a bad prognostic factor of heart failure (61, 62). | **Promote** | Plasma level of VEGFD is up-regulated in heart failure patients, and down-regulated by heart transplantation (63). |
| IDI2 | Related to the functions of muscles. Reduction in expression leads to amyotrophic diseases (64). Genotypic features of IDI2 is also related to left ventricular ejection fractions in patients with hypertension (65), indicating its capability of regulating cardiac muscle contraction. | **Not confirmed** | No current recordings related to its liquid biopsy. However, it was recorded to be detectable in blood plasma according to the Human Protein Atlas(57). |
| RLN2 | Relaxin 2 (expressed by RLN2 gene) has been seen as a drug for curing heart failure, due to its capability in reducing symptoms and promote multiple cardioprotective processes (66). | **Inhibit** | Plasma level of relaxin 2 is up-regulated in patients with congestive heart failure (67, 68), |
| VTCN1 | Downregulate immune responses by suppressing T cell activation and proliferation, meanwhile reducing cytokine production (69). | **Not confirmed** | Expression level of B7-H4 (protein expressed by VTCN1) and its concentration in serum are negatively correlated with cancer prognosis, which is possibly related to their suppressive effects on T-cell activities and inflammatory cytokines (70, 71). |
| ANGPTL3 | High ANGPTL3 leads to improved LDL and increased risks in coronary artery diseases (72). Meanwhile, it promotes angiogenesis in organs (73). | **Promote** | Higher plasma level of ANGPTL3 is associated with higher risks in coronary artery diseases (74). |

**References:**

1. Huan T, Zhang B, Wang Z, Joehanes R, Zhu J, Johnson AD, et al. A Systems Biology Framework Identifies Molecular Underpinnings of Coronary Heart Disease. *Arteriosclerosis, Thrombosis, and Vascular Biology* (2013) 33(6):1427-34. doi: doi:10.1161/ATVBAHA.112.300112.

2. Saunders CN, Chattopadhyay S, Huhn S, Weinhold N, Hoffmann P, Nöthen MM, et al. Search for Al Amyloidosis Risk Factors Using Mendelian Randomization. *Blood Advances* (2021) 5(13):2725-31. doi: 10.1182/bloodadvances.2021004423.

3. Frigyesi I, Adolfsson J, Ali M, Kronborg Christophersen M, Johnsson E, Turesson I, et al. Robust Isolation of Malignant Plasma Cells in Multiple Myeloma. *Blood* (2014) 123(9):1336-40. doi: 10.1182/blood-2013-09-529800.

4. Prox J, Arnold P, Becker-Pauly C. Meprin Α and Meprin Β: Procollagen Proteinases in Health and Disease. *Matrix Biology* (2015) 44-46:7-13. doi: <https://doi.org/10.1016/j.matbio.2015.01.010>.

5. Biasin V, Marsh LM, Egemnazarov B, Wilhelm J, Ghanim B, Klepetko W, et al. Meprin Β, a Novel Mediator of Vascular Remodelling Underlying Pulmonary Hypertension. *The Journal of Pathology* (2014) 233(1):7-17. doi: <https://doi.org/10.1002/path.4303>.

6. Rahn S, Becker-Pauly C. Meprin and Adam Proteases as Triggers of Systemic Inflammation in Sepsis. *FEBS Letters* (2022) 596(5):534-56. doi: <https://doi.org/10.1002/1873-3468.14225>.

7. Kocak A, Avsar AK, Harmanci D, Akdogan G, Birlik AM. A Preliminary Study of Possible Fibrotic Role of Meprin Metalloproteases in Scleroderma Patients. *Arch Rheumatol* (2021) 36(4):510-7. doi: 10.46497/ArchRheumatol.2021.8581.

8. Sun Q, Jin HJ, Bond JS. Disruption of the Meprin Α and Β Genes in Mice Alters Homeostasis of Monocytes and Natural Killer Cells. *Experimental Hematology* (2009) 37(3):346-56. doi: <https://doi.org/10.1016/j.exphem.2008.10.016>.

9. Li Y-J, Fan Y-H, Tang J, Li J-B, Yu C-H. Meprin-Β Regulates Production of Pro-Inflammatory Factors Via a Disintegrin and Metalloproteinase-10 (Adam-10) Dependent Pathway in Macrophages. *International Immunopharmacology* (2014) 18(1):77-84. doi: <https://doi.org/10.1016/j.intimp.2013.11.004>.

10. König K, Marth L, Roissant J, Granja T, Jennewein C, Devanathan V, et al. The Plexin C1 Receptor Promotes Acute Inflammation. *European Journal of Immunology* (2014) 44(9):2648-58. doi: <https://doi.org/10.1002/eji.201343968>.

11. Zhang H, Bredewold EOW, Vreeken D, Duijs JMGJ, de Boer HC, Kraaijeveld AO, et al. Prediction Power on Cardiovascular Disease of Neuroimmune Guidance Cues Expression by Peripheral Blood Monocytes Determined by Machine-Learning Methods. *International Journal of Molecular Sciences* (2020) 21(17):6364.

12. Olofsson PS. Targeting T Cell Costimulation to Prevent Atherothrombosis. *Circ Res* (2012) 110(6):800-1. doi: doi:10.1161/CIRCRESAHA.112.265108.

13. Egerstedt A, Berntsson J, Smith ML, Gidlöf O, Nilsson R, Benson M, et al. Profiling of the Plasma Proteome across Different Stages of Human Heart Failure. *Nature communications* (2019) 10(1):1-13.

14. Ferreira JP, Verdonschot J, Wang P, Pizard A, Collier T, Ahmed FZ, et al. Proteomic and Mechanistic Analysis of Spironolactone in Patients at Risk for Hf. *JACC: Heart Failure* (2021) 9(4):268-77. doi: doi:10.1016/j.jchf.2020.11.010.

15. Gil-Cayuela C, Ortega A, Tarazón E, Martínez-Dolz L, Cinca J, González-Juanatey JR, et al. Myocardium of Patients with Dilated Cardiomyopathy Presents Altered Expression of Genes Involved in Thyroid Hormone Biosynthesis. *Plos One* (2018) 13(1):e0190987. Epub 2018/01/11. doi: 10.1371/journal.pone.0190987.

16. Milanesi E, Manda G, Dobre M, Codrici E, Neagoe IV, Popescu BO, et al. Distinctive under-Expression Profile of Inflammatory and Redox Genes in the Blood of Elderly Patients with Cardiovascular Disease. *J Inflamm Res* (2021) 14:429-42. Epub 2021/03/05. doi: 10.2147/jir.S280328.

17. Young D, Popovic ZB, Jones WK, Gupta S. Blockade of Nf-Κb Using Iκbα Dominant-Negative Mice Ameliorates Cardiac Hypertrophy in Myotrophin-Overexpressed Transgenic Mice. *Journal of Molecular Biology* (2008) 381(3):559-68. doi: <https://doi.org/10.1016/j.jmb.2008.05.076>.

18. Leung E, Lehnert KB, Kanwar JR, Yang Y, Mon Y, McNeil HP, et al. Bioassay Detects Soluble Madcam-1 in Body Fluids. *Immunology & Cell Biology* (2004) 82(4):400-9. doi: <https://doi.org/10.1111/j.0818-9641.2004.01247.x>.

19. Wongsurawat T, Woo CC, Giannakakis A, Lin XY, Cheow ESH, Lee CN, et al. Transcriptome Alterations of Vascular Smooth Muscle Cells in Aortic Wall of Myocardial Infarction Patients. *Data Brief* (2018) 17:1112-35. Epub 2018/06/08. doi: 10.1016/j.dib.2018.01.108.

20. Afshari P, Yao WD, Middleton FA. Reduced Slc1a1 Expression Is Associated with Neuroinflammation and Impaired Sensorimotor Gating and Cognitive Performance in Mice: Implications for Schizophrenia. *Plos One* (2017) 12(9):e0183854. Epub 2017/09/09. doi: 10.1371/journal.pone.0183854.

21. Zannad F, Ferreira JP, Butler J, Filippatos G, Januzzi JL, Sumin M, et al. Effect of Empagliflozin on Circulating Proteomics in Heart Failure: Mechanistic Insights into the Emperor Programme. *European Heart Journal* (2022) 43(48):4991-5002. doi: 10.1093/eurheartj/ehac495.

22. Hassfeld S, Eichhorn C, Stehr K, Naegele H, Geier C, Steeg M, et al. Insulin-Like Growth Factor-Binding Proteins 2 and 3 Are Independent Predictors of a Poor Prognosis in Patients with Dilated Cardiomyopathy. *Heart* (2007) 93(3):359-60. Epub 2007/02/27. doi: 10.1136/hrt.2006.090092.

23. Anwar A, Gaspoz J-M, Pampallona S, Zahid AA, Sigaud P, Pichard C, et al. Effect of Congestive Heart Failure on the Insulin-Like Growth Factor-1 System. *American Journal of Cardiology* (2002) 90(12):1402-5. doi: 10.1016/S0002-9149(02)02885-0.

24. Karhadkar TR, Pilling D, Cox N, Gomer RH. Sialidase Inhibitors Attenuate Pulmonary Fibrosis in a Mouse Model. *Scientific Reports* (2017) 7(1):15069. doi: 10.1038/s41598-017-15198-8.

25. Ghiroldi A, Ciconte G, Creo P, Tarantino A, Melgari D, D’Imperio S, et al. Alterations of the Sialylation Machinery in Brugada Syndrome. *International Journal of Molecular Sciences* (2022) 23(21):13154.

26. Verdonschot JAJ, Wang P, Van Bilsen M, Hazebroek MR, Merken JJ, Vanhoutte EK, et al. Metabolic Profiling Associates with Disease Severity in Nonischemic Dilated Cardiomyopathy. *Journal of Cardiac Failure* (2020) 26(3):212-22. doi: <https://doi.org/10.1016/j.cardfail.2019.09.004>.

27. Cao Y, Wang Y, Zhou Z, Pan C, Jiang L, Zhou Z, et al. Liver-Heart Cross-Talk Mediated by Coagulation Factor Xi Protects against Heart Failure. *Science* (2022) 377(6613):1399-406. Epub 2022/09/23. doi: 10.1126/science.abn0910.

28. Zabczyk M, Butenas S, Palka I, Nessler J, Undas A. Active Tissue Factor and Activated Factor Xi in Circulating Blood of Patients with Systolic Heart Failure Due to Ischemic Cardiomyopathy. *Pol Arch Med Wewn* (2010) 120(9):334-40. Epub 2010/09/25.

29. Mouton J, Loos B, Moolman-Smook JC, Kinnear CJ. Ascribing Novel Functions to the Sarcomeric Protein, Myosin Binding Protein H (Mybph) in Cardiac Sarcomere Contraction. *Experimental Cell Research* (2015) 331(2):338-51. doi: <https://doi.org/10.1016/j.yexcr.2014.11.006>.

30. Alyonycheva T, Cohen-Gould L, Siewert C, Fischman DA, Mikawa T. Skeletal Muscle–Specific Myosin Binding Protein-H Is Expressed in Purkinje Fibers of the Cardiac Conduction System. *Circ Res* (1997) 80(5):665-72. doi: doi:10.1161/01.RES.80.5.665.

31. Zhu T, He Y, Yang J, Fu W, Xu X, Si Y. Mybph Inhibits Vascular Smooth Muscle Cell Migration and Attenuates Neointimal Hyperplasia in a Rat Carotid Balloon-Injury Model. *Experimental Cell Research* (2017) 359(1):154-62. doi: <https://doi.org/10.1016/j.yexcr.2017.07.036>.

32. Ning YL, Yang ZQ, Xian SX, Lin JZ, Lin XF, Chen WT. Bioinformatics Analysis Identifies Hub Genes and Molecular Pathways Involved in Sepsis-Induced Myopathy. *Med Sci Monit* (2020) 26:e919665. Epub 2020/02/03. doi: 10.12659/msm.919665.

33. Logan JG, Yun S, Bao Y, Farber E, Farber CR. Rna-Sequencing Analysis of Differential Gene Expression Associated with Arterial Stiffness. *Vascular* (2020) 28(5):655-63. doi: 10.1177/1708538120922650.

34. Gao F, Hu X, Liu W, Wu H, Mu Y, Zhao Y. Calcium-Activated Nucleotides 1 (Cant1)-Driven Nuclear Factor-K-Gene Binding (Nf-ĸb) Signaling Pathway Facilitates the Lung Cancer Progression. *Bioengineered* (2022) 13(2):3183-93. Epub 2022/01/25. doi: 10.1080/21655979.2021.2003131.

35. Paganini C, Monti L, Costantini R, Besio R, Lecci S, Biggiogera M, et al. Calcium Activated Nucleotidase 1 (Cant1) Is Critical for Glycosaminoglycan Biosynthesis in Cartilage and Endochondral Ossification. *Matrix Biology* (2019) 81:70-90. doi: <https://doi.org/10.1016/j.matbio.2018.11.002>.

36. Li Z, Cui Y, Feng J, Guo Y. Identifying the Pattern of Immune Related Cells and Genes in the Peripheral Blood of Ischemic Stroke. *Journal of Translational Medicine* (2020) 18(1):296. doi: 10.1186/s12967-020-02463-0.

37. Shen H, Xie K, Li M, Yang Q, Wang X. N6-Methyladenosine (M6a) Methyltransferase Mettl3 Regulates Sepsis-Induced Myocardial Injury through Igf2bp1/Hdac4 Dependent Manner. *Cell Death Discovery* (2022) 8(1):322. doi: 10.1038/s41420-022-01099-x.

38. Xie J, Li Q, Zhu X-h, Gao Y, Zhao W-h. Igf2bp1 Promotes Lps-Induced Nfκb Activation and Pro-Inflammatory Cytokines Production in Human Macrophages and monocytes. *Biochemical and Biophysical Research Communications* (2019) 513(4):820-6. doi: <https://doi.org/10.1016/j.bbrc.2019.03.206>.

39. Qian N, Gao Y, Wang J, Wang Y. Emerging Role of Interleukin-13 in Cardiovascular Diseases: A Ray of Hope. *J Cell Mol Med* (2021) 25(12):5351-7. Epub 2021/05/05. doi: 10.1111/jcmm.16566.

40. Nishimura Y, Inoue T, Nitto T, Morooka T, Node K. Increased Interleukin-13 Levels in Patients with Chronic Heart Failure. *International Journal of Cardiology* (2009) 131(3):421-3. doi: 10.1016/j.ijcard.2007.07.128.

41. Wei J, Cui J. Mir-1322 Protects against the Myocardial Ischemia Via Lrp8/Pi3k/Akt Pathway. *Biochemical and Biophysical Research Communications* (2023) 638:120-6. doi: <https://doi.org/10.1016/j.bbrc.2022.10.101>.

42. Yang XV, Banerjee Y, Fernández JA, Deguchi H, Xu X, Mosnier LO, et al. Activated Protein C Ligation of Apoer2 (Lrp8) Causes Dab1-Dependent Signaling in U937 Cells. *Proc Natl Acad Sci U S A* (2009) 106(1):274-9. Epub 2009/01/01. doi: 10.1073/pnas.0807594106.

43. Bachmann JC, Baumgart SJ, Uryga AK, Bosteen MH, Borghetti G, Nyberg M, et al. Fibrotic Signaling in Cardiac Fibroblasts and Vascular Smooth Muscle Cells: The Dual Roles of Fibrosis in Hfpef and Cad. *Cells* (2022) 11(10):1657.

44. Foulquier S, Daskalopoulos EP, Lluri G, Hermans KCM, Deb A, Blankesteijn WM. Wnt Signaling in Cardiac and Vascular Disease. *Pharmacological Reviews* (2018) 70(1):68-. doi: 10.1124/pr.117.013896.

45. Ngo D, Sinha S, Shen D, Kuhn EW, Keyes MJ, Shi X, et al. Aptamer-Based Proteomic Profiling Reveals Novel Candidate Biomarkers and Pathways in Cardiovascular Disease. *Circulation* (2016) 134(4):270-85. Epub 2016/07/23. doi: 10.1161/circulationaha.116.021803.

46. Yin X, Subramanian S, Hwang S-J, O’Donnell CJ, Fox CS, Courchesne P, et al. Protein Biomarkers of New-Onset Cardiovascular Disease. *Arteriosclerosis, Thrombosis, and Vascular Biology* (2014) 34(4):939-45. doi: doi:10.1161/ATVBAHA.113.302918.

47. Parissis JT, Adamopoulos SN, Venetsanou KF, Karas SM, Kremastinos DT. Elevated Plasma Amylase Levels in Advanced Chronic Heart Failure Secondary to Ischemic or Idiopathic Dilated Cardiomyopathy: Correlation with Circulating Interleukin-6 Activity. *Journal of Interferon & Cytokine Research* (2003) 23(6):329-33. doi: 10.1089/107999003766628179.

48. Landry NM, Rattan SG, Filomeno KL, Meier TW, Meier SC, Foran SJ, et al. Ski Activates the Hippo Pathway Via Limd1 to Inhibit Cardiac Fibroblast Activation. *Basic research in cardiology* (2021) 116(1):25. doi: 10.1007/s00395-021-00865-9.

49. Garcia-Campos A, Correia CN, Naranjo-Lucena A, Garza-Cuartero L, Farries G, Browne JA, et al. Fasciola Hepatica Infection in Cattle: Analyzing Responses of Peripheral Blood Mononuclear Cells (Pbmc) Using a Transcriptomics Approach. *Front Immunol* (2019) 10:2081-. doi: 10.3389/fimmu.2019.02081.

50. di Salvo TG, Yang K-C, Brittain E, Absi T, Maltais S, Hemnes A. Right Ventricular Myocardial Biomarkers in Human Heart Failure. *Journal of Cardiac Failure* (2015) 21(5):398-411. doi: <https://doi.org/10.1016/j.cardfail.2015.02.005>.

51. Xie Z, Shen Y, Huang S, Shen W, Liu J. Abnormal Adamts2 and Vsig4 in Serum of Hf Patients and Their Relationship with Crp, Ua, and Hcy. *Clin Lab* (2022) 68(5). Epub 2022/05/11. doi: 10.7754/Clin.Lab.2021.210811.

52. Barallobre-Barreiro J, Radovits T, Fava M, Mayr U, Lin W-Y, Ermolaeva E, et al. Extracellular Matrix in Heart Failure: Role of Adamts5 in Proteoglycan Remodeling. *Circulation* (2021) 144(25):2021-34. doi: doi:10.1161/CIRCULATIONAHA.121.055732.

53. Wang Z, Ye D, Ye J, Wang M, Liu J, Jiang H, et al. Adamts-5 Decreases in Coronary Arteries and Plasma from Patients with Coronary Artery Disease. *Disease Markers* (2019) 2019:6129748. doi: 10.1155/2019/6129748.

54. Kryza T, Parent C, Pardessus J, Petit A, Burlaud-Gaillard J, Reverdiau P, et al. Human Kallikrein-Related Peptidase 12 Stimulates Endothelial Cell Migration by Remodeling the Fibronectin Matrix. *Scientific Reports* (2018) 8(1):6331. doi: 10.1038/s41598-018-24576-9.

55. Du J-K, Yu Q, Liu Y-J, Du S-F, Huang L-Y, Xu D-H, et al. A Novel Role of Kallikrein-Related Peptidase 8 in the Pathogenesis of Diabetic Cardiac Fibrosis. *Theranostics* (2021) 11(9):4207-31. doi: 10.7150/thno.48530.

56. Sarkar S, Leaman DW, Gupta S, Sil P, Young D, Morehead A, et al. Cardiac Overexpression of Myotrophin Triggers Myocardial Hypertrophy and Heart Failure in Transgenic Mice *. *Journal of Biological Chemistry* (2004) 279(19):20422-34. doi: 10.1074/jbc.M308488200.

57. Uhlén M, Fagerberg L, Hallström BM, Lindskog C, Oksvold P, Mardinoglu A, et al. Tissue-Based Map of the Human Proteome. *Science* (2015) 347(6220):1260419. doi: doi:10.1126/science.1260419.

58. Toma M, Mak GJ, Chen V, Hollander Z, Shannon CP, Lam KKY, et al. Differentiating Heart Failure Phenotypes Using Sex-Specific Transcriptomic and Proteomic Biomarker Panels. *ESC Heart Fail* (2017) 4(3):301-11. Epub 2017/08/05. doi: 10.1002/ehf2.12136.

59. Fu Y, Ge Y, Cao J, Su Z, Yu D. Identification of Key Exosome Gene Signature in Mediating Coronary Heart Disease by Weighted Gene Correlation Network Analysis. *Biomed Res Int* (2021) 2021:3440498. Epub 2021/10/26. doi: 10.1155/2021/3440498.

60. Grois L, Hupf J, Reinders J, Schröder J, Dietl A, Schmid PM, et al. Combined Inhibition of the Renin-Angiotensin System and Neprilysin Positively Influences Complex Mitochondrial Adaptations in Progressive Experimental Heart Failure. *Plos One* (2017) 12(1):e0169743. doi: 10.1371/journal.pone.0169743.

61. Zhou Y, Zhu X, Cui H, Shi J, Yuan G, Shi S, et al. The Role of the Vegf Family in Coronary Heart Disease. *Frontiers in Cardiovascular Medicine* (2021) 8. doi: 10.3389/fcvm.2021.738325.

62. Iguchi M, Wada H, Shinozaki T, Suzuki M, Ajiro Y, Matsuda M, et al. Distinct Association of Vegf-C and Vegf-D with Prognosis in Patients with Chronic Heart Failure: The Prehosp-Chf Study. *European Heart Journal* (2021) 42(Supplement_1). doi: 10.1093/eurheartj/ehab724.0868.

63. Ahmed S, Ahmed A, Säleby J, Bouzina H, Lundgren J, Rådegran G. Elevated Plasma Tyrosine Kinases Vegf-D and Her4 in Heart Failure Patients Decrease after Heart Transplantation in Association with Improved Haemodynamics. *Heart Vessels* (2020) 35(6):786-99. Epub 2020/01/22. doi: 10.1007/s00380-019-01548-1.

64. Kato T, Emi M, Sato H, Arawaka S, Wada M, Kawanami T, et al. Segmental Copy-Number Gain within the Region of Isopentenyl Diphosphate Isomerase Genes in Sporadic Amyotrophic Lateral Sclerosis. *Biochemical and Biophysical Research Communications* (2010) 402(2):438-42. doi: <https://doi.org/10.1016/j.bbrc.2010.10.056>.

65. Luo Y, Mao C, Yang Y, Wang F, Ahmad FS, Arnett D, et al. Integrating Hypertension Phenotype and Genotype with Hybrid Non-Negative Matrix Factorization. *Bioinformatics* (2018) 35(8):1395-403. doi: 10.1093/bioinformatics/bty804.

66. Ponikowski P, Metra M, Teerlink JR, Unemori E, Felker GM, Voors AA, et al. Design of the Relaxin in Acute Heart Failure Study. *American Heart Journal* (2012) 163(2):149-55.e1. doi: <https://doi.org/10.1016/j.ahj.2011.10.009>.

67. Dschietzig T, Richter C, Bartsch C, Laule M, Armbruster FP, Baumann G, et al. The Pregnancy Hormone Relaxin Is a Player in Human Heart Failure. *The FASEB Journal* (2001) 15(12):2187-95. doi: <https://doi.org/10.1096/fj.01-0070com>.

68. Fisher C, Berry C, Blue L, Morton JJ, McMurray J. N-Terminal Pro B Type Natriuretic Peptide, but Not the New Putative Cardiac Hormone Relaxin, Predicts Prognosis in Patients with Chronic Heart Failure. *Heart* (2003) 89(8):879-81. doi: 10.1136/heart.89.8.879.

69. Vaishnav J, Khan F, Yadav M, Parmar N, Buch H, Jadeja SD, et al. V-Set Domain Containing T-Cell Activation Inhibitor-1 (Vtcn1): A Potential Target for the Treatment of Autoimmune Diseases. *Immunobiology* (2022) 227(6):152274. doi: <https://doi.org/10.1016/j.imbio.2022.152274>.

70. Shi H, Ji M, Wu J, Zhou Q, Li X, Li Z, et al. Serum B7-H4 Expression Is a Significant Prognostic Indicator for Patients with Gastric Cancer. *World Journal of Surgical Oncology* (2014) 12(1):188. doi: 10.1186/1477-7819-12-188.

71. Arigami T, Uenosono Y, Hirata M, Hagihara T, Yanagita S, Ishigami S, et al. Expression of B7-H4 in Blood of Patients with Gastric Cancer Predicts Tumor Progression and Prognosis. *Journal of Surgical Oncology* (2010) 102(7):748-52. doi: <https://doi.org/10.1002/jso.21722>.

72. Dewey FE, Gusarova V, Dunbar RL, O’Dushlaine C, Schurmann C, Gottesman O, et al. Genetic and Pharmacologic Inactivation of Angptl3 and Cardiovascular Disease. *New England Journal of Medicine* (2017) 377(3):211-21. doi: 10.1056/NEJMoa1612790.

73. Camenisch G, Pisabarro MT, Sherman D, Kowalski J, Nagel M, Hass P, et al. Angptl3 Stimulates Endothelial Cell Adhesion and Migration Via Integrin Migration Via Integrin Αvβ3 and Induces Blood Vessel Formation in Vivo. *Journal of Biological Chemistry* (2002) 277(19):17281-90. doi: 10.1074/jbc.M109768200.

74. Lv Q, Jiao X, Yu H, Sun Q, Li F, Wang Y, et al. Angptl3 and Cardiovascular Outcomes in Patients with Acute Coronary Syndrome and Obstructive Sleep Apnea. *Journal of the American Heart Association* (2022) 11(18):e025955. doi: doi:10.1161/JAHA.122.025955.
